# Synthesis and biological profile of 2,3-dihydro[1,3]thiazolo[4,5-*b*]pyridines, a novel class of acyl-ACP thioesterase inhibitors

**DOI:** 10.3762/bjoc.20.46

**Published:** 2024-03-01

**Authors:** Jens Frackenpohl, David M Barber, Guido Bojack, Birgit Bollenbach-Wahl, Ralf Braun, Rahel Getachew, Sabine Hohmann, Kwang-Yoon Ko, Karoline Kurowski, Bernd Laber, Rebecca L Mattison, Thomas Müller, Anna M Reingruber, Dirk Schmutzler, Andrea Svejda

**Affiliations:** 1 Research & Development, Weed Control, Crop Science Division, Bayer AG, Industriepark Höchst, 65926 Frankfurt am Main, Germany

**Keywords:** 2,3-dihydro[1,3]thiazolo[4,5-*b*]pyridine, acyl-ACP thioesterase, bioisostere, herbicide, heterocycle

## Abstract

The present work covers novel herbicidal lead structures that contain a 2,3-dihydro[1,3]thiazolo[4,5-*b*]pyridine scaffold as structural key feature carrying a substituted phenyl side chain. These new compounds show good acyl-ACP thioesterase inhibition in line with strong herbicidal activity against commercially important weeds in broadacre crops, e.g., wheat and corn. The desired substituted 2,3-dihydro[1,3]thiazolo[4,5-*b*]pyridines were prepared via an optimized BH_3_-mediated reduction involving tris(pentafluorophenyl)borane as a strong Lewis acid. Remarkably, greenhouse trials showed that some of the target compounds outlined herein display promising control of grass weed species in preemergence application, combined with a dose response window that enables partial selectivity in certain crops.

## Introduction

**Scheme 1:**
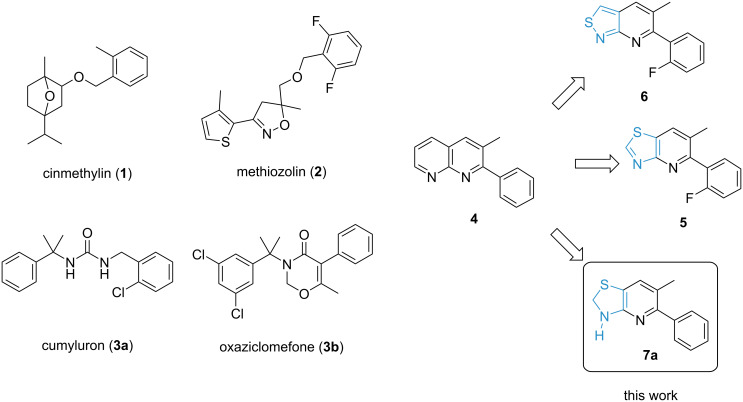
Selected known inhibitors **1**–**3** of acyl-ACP thioesterases (belonging to the protein family of FATs) and new lead structures **4**–**7a**.

The presence of weed infestations exerts a high strain on food production around the globe by depleting resources for the crops and facilitating the transmission of diseases [1]. Although herbicides remain the most effective solution for weed control due to the associated efficiency and simplicity, they face multiple challenges, such as the emergence and growth of resistant weed populations. It is therefore essential that crop protection research acts rapidly to provide farmers with new solutions that enable them to fight back against resistant weed species [2]. Nevertheless, discovering novel and commercially viable modes of action within the timeframe needed to significantly impact the control of resistant weeds is a demanding task. Thus, we analyzed several herbicidal modes of action with emphasis on the structural diversity of small-molecule ligands. In this context, acyl-acyl carrier protein (acyl-ACP) thioesterase inhibitors have shown a remarkable variability. Fatty acid thioesterase (FAT) enzymes represent a family of proteins exclusively found in higher plants. They mediate the release of fatty acids from the plastids to the endoplasmic reticulum, where they are utilized for the synthesis of acyl lipids that are essential components for various physiological and defensive processes [3–6]. As this enzyme target does not exist in other kingdoms, structure–activity relationship (SAR) studies on selective inhibitors reduce the prevalence of undesired effects, such as toxicity in mammals [4]. Despite being employed in the field for over three decades, the mode of action of preemergence herbicide cinmethylin (**1**, Scheme 1) has remained unknown until 2018. At that time, the binding affinity to enzyme targets, e.g., acyl-ACP thioesterases, belonging to the protein family of FATs, was demonstrated by using co-crystallization, fluorescence-based thermal shift assays, and chemoproteomics techniques [3]. Likewise, methiozolin (**2**) is a recently assigned FAT inhibitor that has shown good results in selectively controlling grass weeds in both cool and warm seasons [7–9]. Recently, it has been shown that several herbicides bearing a *gem*-dimethylbenzylamide motif, e.g., cumyluron (**3a**) and oxaziclomefone (**3b**), previously exhibiting an unknown mode of action, control weeds due to the inhibition of FAT [10]. In search for further chemical entities that can control resistant weed species via the inhibition of FAT, we were interested in exploring a compound class containing a 1,8-naphthyridine core that was first reported by BASF, e.g., compound **4** [11].

In contrast to bicyclic cinmethylin (**1**) and methiozolin (**2**), substituted 1,8-naphthyridine **4** does not contain any stereocenters but still displays promising efficacy against grass weeds. Further considering the rather low molecular weight (220 g/mol) and structural simplicity, compound **4** is a highly attractive initial lead structure with ample space for structural variations. By formally replacing one pyridine moiety of 1,8-naphthyridine **4** by a five-membered thiazole unit, we have identified thiazolo[4,5-*b*]pyridine **5** as a strong inhibitor of acyl-ACP thioesterase, which has further been confirmed via an X-ray co-crystal structure [12]. Additionally, greenhouse trials have shown that thiazolopyridine **5** and a large number of closely related analogues display excellent control of grass weed species in preemergence applications [13–14]. Independently, researchers at Syngenta have shown that the pyridine unit in the 1,8-naphthyridine scaffold can also be formally substituted by an isothiazole group, as can be seen in isothiazolo[3,4-*b*]pyridine **6** [15].

Thus, several bicyclic heteroaromatic motifs containing two nitrogen atoms serve as structural surrogates of cinmethylin (**1**), bearing a substituted 7-oxabicyclo[2.2.1]heptane scaffold [16]. Based on the findings outlined above and based on other plant-specific modes of action, it is plausible that FAT inhibitors encompass a broader range of structural motifs [16–17]. Herein, we present our approach to complement heteroaromatic lead structures **4**–**6** by introducing a nonaromatic motif via preparation of the novel 2,3-dihydro[1,3]thiazolo[4,5-*b*]pyridines **7**.

## Results and Discussion

**Scheme 2:**
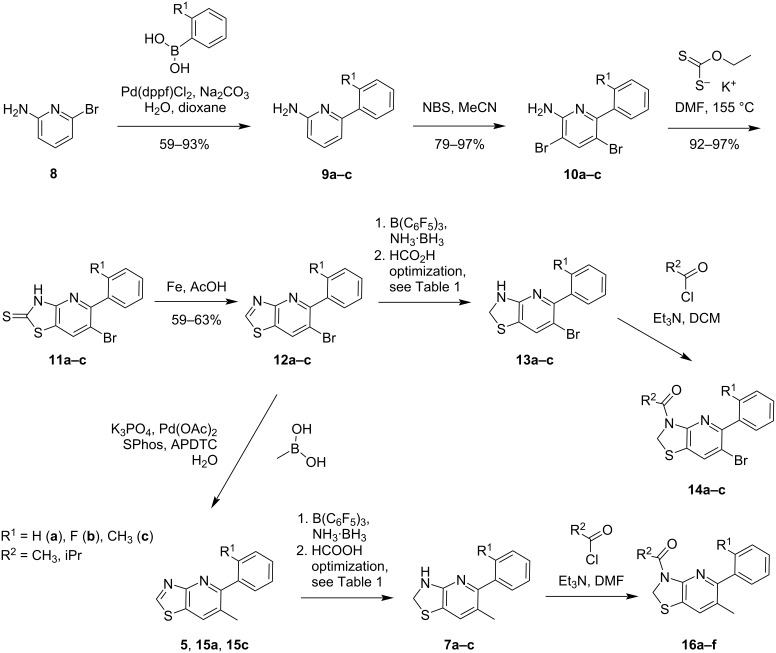
Preparation of 2,3-dihydro[1,3]thiazolo[4,5-*b*]pyridines **7a**–**c** and **13a**–**c** via iron-mediated sulfur removal and subsequent reduction. dppf = 1,1'-bis(diphenylphosphino)ferrocene, SPhos = 2-dicyclohexylphosphino-2',6'-dimethoxybiphenyl, APDTC = ammonium pyrrolidinedithiocarbamate.

Although the 2,3-dihydro[1,3]thiazolo[4,5-*b*]pyridine scaffold looks relatively simple at a first glance, it displays a very different reactivity compared to the parent naphthyridine series. Likewise, 1,8-naphthyridines are easily accessed in high yield and on a multigram scale via Friedländer synthesis [18]. This was in clear contrast to the intermediate thiazolo[4,5-*b*]pyridines and the desired 2,3-dihydro[1,3]thiazolo[4,5-*b*]pyridine that we wanted to access, with approaches to prepare the thiazolo[4,5-*b*]pyridines using the Friedländer synthesis often being met with failure or disappointingly low product yield [12]. We thus particularly emphasized on upscaling, facile workup, and a robust yield for each step. This was due to the potential need for the preparation of multigram quantities of the most active compounds for advanced biological testing. Pleasingly, our four-step approach using a potassium *O*-ethyl dithiocarbonate-mediated formation of thio intermediates **11a**–**c** (thiol–thione tautomers) with subsequent sulfur removal using iron powder in acetic acid [19] proceeded smoothly to afford thiazolopyridines **12a**–**c** in good yield. This allowed us to circumvent the previously employed alkylation–oxidation–reduction sequences (Scheme 2) [12]. Thereupon, we recognized that we could introduce two halogen atoms in the halogenation step and carry one through to the end of the synthetic route, which enabled us to introduce a methyl substituent in this position. Likewise, methyl-substituted thiazolo[4,5-*b*]pyridines **5**, **15a**, and **15c** were synthesized using an optimized Suzuki coupling and served together with compounds **12a**–**c** as key intermediates to explore different reagents and conditions to prepare 2,3-dihydro[1,3]thiazolo[4,5-*b*]pyridines **7a**–**c** and **13a**–**c** via a late-stage reduction (Scheme 2 and Table 1).

**Table 1:** Preparation of 2,3-dihydro[1,3]thiazolo[4,5-*b*]pyridines **7a**–**c** via reduction of the thiazole moiety: optimization of the reaction conditions.^a^

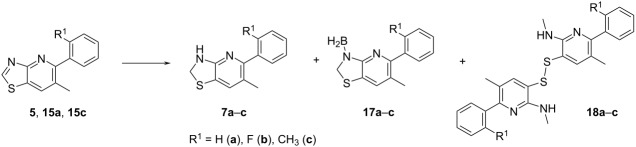								

entry	R^1^	reagents	solvent	*T*, °C	*T*, h	**7a**–**c**, %^b^	**17a**–**c**, %^b^	**18a**–**c**, %^b^

1	CH_3_	H_2_, Pd/C, 2–20 bar	MeOH	20	4	—	—	—
2	F	H_2_, Pd/C, 4–35 bar	MeOH	20	6	—	—	—
3	F	N_2_H_4_, Pd/C	EtOH	80	6	—	—	—
4	F	B_2_(OH)_4_	H_2_O	80	6	—	—	—
5	F	Bu_4_NBH_4_	THF	20	5	—	—	—
6	F	Bu_4_NBH_4_	THF	70	5	5	—	48
7	H	Bu_4_NBH_4_	dioxane	80	5	8	—	59
8	F	NaCNBH_3_, AcOH	MeOH	60	5	4	—	15
9	F	SiCl_3_H	toluene	110	3	—	—	35
10	CH_3_	SiEt_3_H	dioxane	80	3	—	—	43
11	F	NH_3_-BH_3_	toluene	80	4	5	4	12
12	F	NH_3_⋅BH_3_, B(C_6_F_5_)_3_	toluene	80	4	16	33	18
13	CH_3_	NH_3_⋅BH_3_, B(C_6_F_5_)_3_	toluene	80	7	8	29	30
14	CH_3_	NH_3_⋅BH_3_, B(C_6_F_5_)_3_	toluene	45	6	4	51	3
15	H	NH_3_⋅BH_3_, B(C_6_F_5_)_3_, HCO_2_H	toluene	45	7	64^c^	—	2
16	F	NH_3_⋅BH_3_, B(C_6_F_5_)_3_, HCO_2_H	toluene	45	5	66^c^	—	5
17	CH_3_	NH_3_⋅BH_3_, B(C_6_F_5_)_3_, HCO_2_H	toluene	45	7	59^c^	—	4

^a^All reactions in the optimization phase were carried out using 0.2 mmol of **5**, **15a**, and **15c**, respectively. ^b^Determined by analytical HPLC. ^c^Isolated yield after silica gel column chromatography.

Whilst several synthetic approaches towards 2,3-dihydro-1,3-benzothiazoles involving the hydrogenation of 1,3-benzothiazoles have been described [20], the corresponding preparation of 2,3-dihydro[1,3]thiazolo[4,5-*b*]pyridines remained unexplored to our great surprise. Thus, we investigated the conversion of [1,3]thiazolo[4,5-*b*]pyridines **5** (R^1^ = F), **15a** (R^1^ = H), and **15c** (R^1^ = CH_3_) into 2,3-dihydro[1,3]thiazolo[4,5-*b*]pyridines **7a**–**c** thoroughly, with the aim to establish a practicable and robust synthetic route enabling us to carry out a broad SAR study. Initial attempts to prepare 2,3-dihydro[1,3]thiazolo[4,5-*b*]pyridines **7a** and **7b** using hydrogen and palladium on charcoal under elevated pressure did not show any conversion of the starting material (Table 1, entries 1 and 2). Correspondingly, [1,3]thiazolo[4,5-*b*]pyridine **5** remained unchanged upon application of methods that had been successfully utilized in the hydrogenation of 1,3-benzothiazoles, involving diboronic acid or hydrazine hydrate as key reagents [21] in protic solvents at an elevated temperature (Table 1, entries 3 and 4). Whilst tetrabutylammonium borohydride [22] at room temperature did not lead to a conversion of the starting material **5** either, a trace amount of desired 2,3-dihydro[1,3]thiazolo[4,5-*b*]pyridine **7b** was formed at elevated temperature, accompanied by disulfide **18b** as the main product (Table 1, entries 5 and 6). This result indicated that borohydride reagents were able to activate the thiazole moiety in [1,3]thiazolo[4,5-*b*]pyridines, leaving the pyridine unit unchanged. While sodium cyanoborohydride afforded a comparable result, albeit with lower conversion, the use of silane reagents at elevated temperature [23] led to the cleavage of the thiazole ring, furnishing disulfides **18b** and **18c** exclusively (Table 1, entries 7–10). Interestingly, the reaction of **5** with ammonia borane at elevated temperature in toluene [20] furnished three reaction products with a low yield since 2,3-dihydro[1,3]thiazolo[4,5-*b*]pyridine **7b** was formed together with disulfide **18b** and aminoborane **17b** (Table 1, entry 11). We thus evaluated B(C_6_F_5_)_3_ as a nonmetallic catalyst to activate ammonia borane in the reductive hydrogenation of the C=N-bond in [1,3]thiazolo[4,5-*b*]pyridines **5** and **15c**. In line with reports on the hydrogenation of quinolines and indoles [24], pyridines [25], and imines [26], the reactions of **5** and **15c** with ammonia borane (3 equiv) in the presence of a catalytic amount of B(C_6_F_5_)_3_ in toluene as an aprotic solvent at a temperature of 80 °C afforded aminoboranes **17b** (R^1^ = F) and **17c** (R^1^ = CH_3_) as main products along with desired target compounds **7b** and **7c** (Table 1, entries 12 and 13). However, disulfides were still formed as side products in a significant amount. Pleasingly, the undesired thiazole cleavage could successfully be minimized by reducing the reaction temperature to 45 °C, furnishing aminoborane **17c** in 51% yield (Table 1, entry 14). The borane group could be cleaved off easily by the subsequent treatment of **17c** with formic acid in acetonitrile, affording 2,3-dihydro[1,3]thiazolo[4,5-*b*]pyridine **7c** as the only reaction product. By applying this optimized two-step procedure to [1,3]thiazolo[4,5-*b*]pyridines **5**, **15a**, and **15c**, the desired 2,3-dihydro[1,3]thiazolo[4,5-*b*]pyridines **7a**–**c** were prepared in good yield (Table 1, entries 15–17, 59–66% isolated yield), enabling us to investigate the biological profiles as well as the reactions with acyl chlorides to form amides **16a**–**f** (Scheme 2) [27]. These acylations proceeded cleanly under mild conditions, using the corresponding acyl chloride together with triethylamine as a suitable base in DCM.

**Scheme 3:**
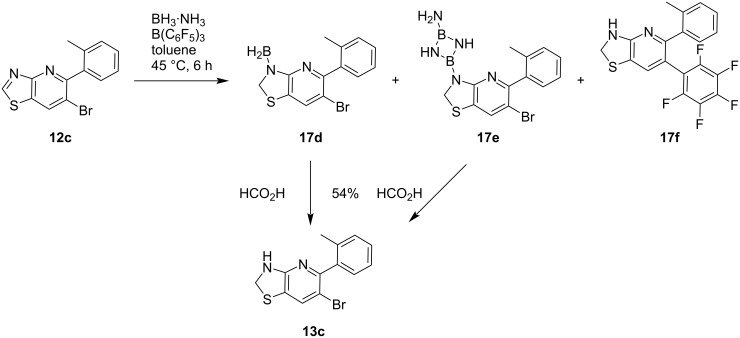
Evaluation of potential side reactions in the borane-mediated preparation of 2,3-dihydro[1,3]thiazolo[4,5-*b*]pyridine **13c**.

Furthermore, we evaluated the tolerance of [1,3]thiazolo[4,5-*b*]pyridines **12a**–**c**, containing a bromine atom, towards the optimized B(C_6_F_5_)_3_-mediated reduction. In good accordance with the results obtained for dimethylated [1,3]thiazolo[4,5-*b*]pyridine **15c**, the corresponding aminoboranes **17d** and **17e** were formed when 6-bromo[1,3]thiazolo[4,5-*b*]pyridine **12c** was treated with ammonia borane in toluene at 45 °C in the presence of a catalytic amount of B(C_6_F_5_)_3_ (Scheme 3). Whilst diphenyl analogue **17f** was isolated as a side product upon arylation with B(C_6_F_5_)_3_, debromination was only observed in traces. Both aminoboranes **17d** and **17e** were then cleaved separately in clean conversions using formic acid to afford the desired substituted 6-bromo-5-(2-tolyl)-2,3-dihydrothiazolo[4,5-b]pyridine (**13c**, 54% combined isolated yield). As shown for N-acylated target compounds **16a**–**f**, the acylation of 6-bromo-2,3-dihydro[1,3]thiazolo[4,5-*b*]pyridines **13a**–**c**, affording target compounds **14a**–**c**, proceeded under mild conditions with a suitable acyl chloride reagent and triethylamine as base in DCM. It was not necessary to add a further base to activate the thiazoline nitrogen atom. Following the aforementioned two-step procedure, more than 15 unprecedented 2,3-dihydro[1,3]thiazolo[4,5-*b*]pyridines bearing different substituents were obtained for biological and biochemical tests.

Converting the thiazole moiety into a thiazoline unit had a measurable impact on several physicochemical parameters, such as LogP and water solubility. Whilst thiazolo[4,5-*b*]pyridine **5** afforded a moderate water solubility of 49 mg/L, paired with a LogP of 2.28 (pH 2.3), the corresponding 2,3-dihydro[1,3]thiazolo[4,5-*b*]pyridine **7b** had a higher water solubility of 173 mg/L and a lower LogP of 1.59 (pH 2.3). However, the lipophilicity of the new 2,3-dihydro[1,3]thiazolo[4,5-*b*]pyridines was highly dependent on the substituents. For example, the brominated analogs **13b** and **13c** showed considerably higher LogP values of 2.88 (i.e., **13b**) and 3.17 (i.e., **13c**). We were thus curious to see how the structural change from a heteroaromatic thiazole unit to a partially saturated thiazoline moiety affected the in vitro and in vivo efficacy of the target compounds.

All compounds that were prepared to explore the SAR of substituted 2,3-dihydro[1,3]thiazolo[4,5-*b*]pyridines, i.e., **13a**–**c**, and **7a**–**c**, the acylated analogues **14a**–**c** and **16a**–**f**, as well as selected aminoboranes **17d** and **17e**, were tested for target affinity in dedicated in vitro tests, as well as for herbicidal effects in vivo upon preemergence application to plants. Based on our experience with thiazolopyridine-based FAT inhibitors [12–13], five representative grass weeds (ALOMY, ECHCG, LOLRI, POAAN, and SETVI) were chosen as model plants to assess initial preemergence activity using a dose rate of 320 g/ha, whereas in vitro tests were carried out using FAT A, isolated from duckweed (*Lemna paucicostata*, LEMPA, *Lp*). As outlined in Table 2, entries 19 and 20, cinmethylin (**1**) and methiozolin (**2**) proved to be suitable commercial reference compounds. They showed good and broad control of grass weeds in various test systems, albeit with incomplete control of the commercially important grass weed LOLRI, paired with insufficient control of ECHCG by methiozolin (**2**) in our greenhouse tests. Furthermore, we used thiazolo[4,5-*b*]pyridine **5** as a strong internal standard to assess how modification of the thiazole moiety would affected the biological activity. It is worth noting that we emphasized investigating the preemergence efficacy as this application type is still important for cereals.

**Table 2:** Preemergence in vivo efficacy screening of 2,3-dihydro[1,3]thiazolo[4,5-*b*]pyridines **7a**–**c** and **13a**–**c** as well as of N-acylated analogs **14a**–**c** and **16a**–**f** against selected monocotyledon weeds, and binding affinity to FAT A from LEMPA.

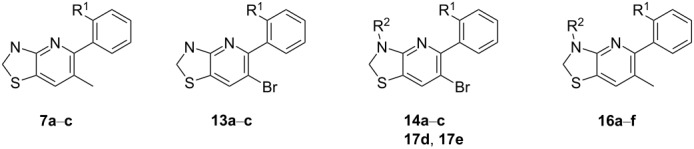									

entry	compound	R^1^	R^2^	pI_50_^a^	ALOMY^b,c^	ECHCG^b,c^	POAAN^b,c^	SETVI^b,c^	LOLRI^b,c^

1	**7a**	H	H	5.9	5	5	5	5	5
2	**7b**	F	H	6.1	5	5	5	5	5
3	**7c**	CH_3_	H	6.3	5	5	5	5	5
4	**13a**	H	H	5.7	4	5	5	5	3
5	**13b**	F	H	7.3	5	5	5	5	5
6	**13c**	CH_3_	H	4.5	1	5	1	5	1
7	**14a**	H	Ac	5.5	4	5	5	5	5
8	**14b**	F	iBtr^d^	4.7	3	3	3	5	2
9	**14c**	CH_3_	Ac	4.3	3	3	3	5	1
10	**16a**	H	Ac	5.5	4	5	5	5	5
11	**16b**	H	iBtr	4.7	3	3	5	5	4
12	**16c**	F	Ac	5.3	4	4	5	5	4
13	**16d**	F	iBtr	4.7	3	5	5	5	4
14	**16e**	CH_3_	Ac	4.1	3	3	5	4	3
15	**16f**	CH_3_	iBtr	4.8	3	5	5	5	3
16	**17d**	CH_3_	BH_2_	4.8	2	5	3	5	3
17	**17e**	CH_3_	BX_2_^e^	<4.0	1	3	3	5	1
18	**5**	F	—	7.2	5	5	5	5	5
19	**1**			6.8	5	5	4	4	1
20	**2**			7.1	4	1	5	5	1

^a^In vitro inhibition of FAT A (from LEMPA). ^b^*n* = 10, i.e., 10 monocotyledonous weed seeds were grown per pot. ^c^Dose rate 320 g/ha. Efficacy values are given based on a rating scale by final visual experts’ assessment of green mass, i.e., 5 ≥ 90% inhibition, 4 = 70–89% inhibition, 3 = 50–69% inhibition, 2 = 40–49% inhibition, 1 = 21–39% inhibition, and — < 20% inhibition. Cinmethylin (**1**), methiozolin (**2**), and thiazolo[4,5-*b*]pyridine **5** were used as comparative internal standards. ^d^Isobutyryl. ^e^BX_2_ = 1,3,2,4-diazadiboretidin-2-amine.

Firstly, we investigated 6-methyl-2,3-dihydro[1,3]thiazolo[4,5-*b*]pyridines **7a**–**c** containing a variously substituted phenyl moiety. Moderate receptor affinity towards FAT A isolated from LEMPA could be observed for all three target compounds **7a**–**c** (pI_50_ 5.9–6.3, Table 2, entries 1–3). However, this was paired with strong in vivo efficacy on the level of internal standard **5** and exceeding the results obtained for cinmethylin (**1**) and methiozolin (**2**). Whilst compounds **7a**–**c** afforded complete control of all tested weeds at an application rate of 320 g/ha, the corresponding 6-bromo analogues **13a**–**c** showed higher sensitivity towards changes in the phenyl moiety. 6-Bromo-5-phenyl-2,3-dihydrothiazolo[4,5-*b*]pyridine (**13a**) showed only partial control of ALOMY and LOLRI, combined with moderate target affinity (pI_50_ 5.7), whereas compound **13c**, bearing an *o*-tolyl substituent, provided insufficient control of three weeds, i.e., ALOMY, POAAN, and LOLRI (Table 2, entry 6), in line with a surprisingly weak target affinity (pI_50_ 4.5). Remarkably, 6-bromo-5-(2-fluorophenyl)-2,3-dihydro[1,3]thiazolo[4,5-*b*]pyridine (**13b**) exhibited the highest affinity towards FAT A of all new analogues (pI_50_ 7.3), paired with full control of all tested weeds (Table 2, entry 5). As shown by the results outlined for 2,3-dihydro[1,3]thiazolo[4,5-*b*]pyridines **7a**–**c** and **13a**–**c** (Table 2, entries 1–6) our approach to introduce a thiazoline moiety via hydrogenation was well tolerated with respect to in vivo efficacy, albeit paired with reduced target affinity except for **13b**. Likewise, the N-acylated 2,3-dihydro[1,3]thiazolo[4,5-*b*]pyridines **14a**–**c** and **16a**–**f** afforded a moderate target affinity in line with moderate to good control of the tested weeds (Table 2, entries 7–15). Interestingly, N-acylated analogues **14a** and **16a**, both bearing an unsubstituted phenyl substituent, delivered the best in vivo results, fully controlling four grass weeds, including commercially important LOLRI, and only showing partial control of ALOMY (Table 2, entries 7 and 10). Thus, both N-acylated compounds afforded nearly the same level of in vivo efficacy as the parent 2,3-dihydro[1,3]thiazolo[4,5-*b*]pyridines **13a** and **7a**. Aminoboranes **17d** and **17e** also showed partial control of the tested grass weeds, albeit on a significantly lower level. Accordingly, the target binding affinities were considerably lower than those measured for the strongest analogues **7b**, **7c**, and **13b**.

**Figure 1:**
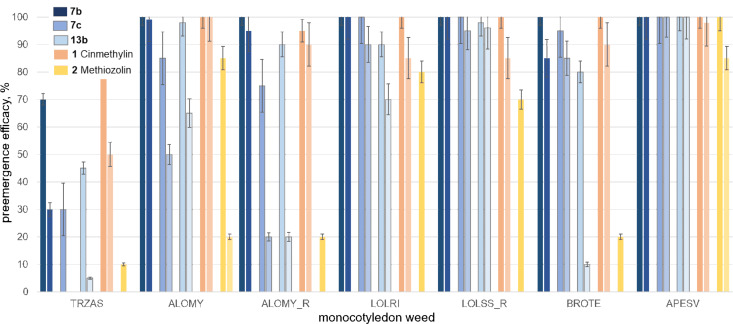
Preemergence efficacy of 2,3-dihydro[1,3]thiazolo[4,5-*b*]pyridine-based FAT inhibitors **7b**, **7c**, and **13b** as well as internal standards cinmethylin (**1**) and methiozolin (**2**) against selected resistant and nonresistant monocotyledon weeds in wheat at application rates of 200 and 50 g/ha in advanced greenhouse trials, e.g., LOLSS_R and ALOMY_R (replicates: 10 plants per pot).

To gain further insights into the biological profile, we chose compounds **7b**, **7c**, and **13b** with promising initial in vivo activity as representatives of our new class of FAT inhibitors to assess the activity in advanced greenhouse tests (i.e., more replicates, larger pots, and lower application rate). Emphasis was put on the efficacy against resistant weeds and on crop selectivity profiles. We thus expanded our investigations to resistant monocotyledon weed species, i.e., resistant blackgrass (ALOMY_R, also known as black twitch or slender foxtail) and ryegrass (LOLSS_R), together with nonresistant ALOMY, LOLRI, APESV, and BROTE as commercially relevant target weeds, and wheat (TRZAS) as the crop (Figure 1).

**Figure 2:**
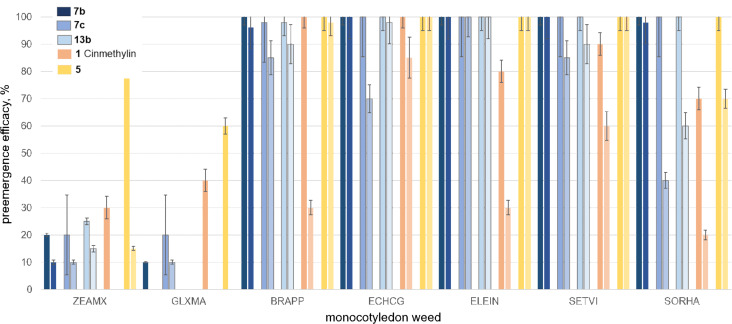
Preemergence efficacy of 2,3-dihydro[1,3]thiazolo[4,5-*b*]pyridine-based FAT inhibitors **7b**, **7c**, and **13b** as well as internal standards cinmethylin (**1**) and [1,3]thiazolo[4,5-*b*]pyridine **5** against selected warm-season monocotyledon weeds in corn and soy at application rates of 150 and 50 g/ha in advanced greenhouse trials, e.g., BRAPP and ELEIN (replicates: 10 plants per pot).

Whilst all three new 2,3-dihydro[1,3]thiazolo[4,5-*b*]pyridine-based FAT inhibitors exceeded the efficacy of commercial standard methiozolin (**2**), target compound **7b** controlled grass weeds on the same level or slightly better than cinmethylin (**1**). Accordingly, application of **7c**, **13b**, and **2** resulted in low crop damage in wheat (in particular at an application rate of 50 g/ha), whereas compounds **7b** and **1** exhibited higher crop damage in the test systems on a comparable level. Methiozolin (**2**) only showed moderate control of nonresistant ALOMY (85% at 200 g/ha and 20% at 50 g/ha vs more than 90% control at both application rates by **7b**, Figure 1). Likewise, **2** had only marginal effects against ALOMY_R and LOLSS_R. Whilst 2,3-dihydro[1,3]thiazolo[4,5-*b*]pyridines **7c** and **13b** showed weak to moderate efficacy against ALOMY_R (strongly dependent on the application rate), *o*-fluorophenyl analogue **7b** exhibited strong control of this resistant, commercially important grass weed. Remarkably, all three 2,3-dihydro[1,3]thiazolo[4,5-*b*]pyridines **7b**, **7c**, and **13b** afforded very good control of the second resistant monocotyledon weed LOLSS_R, reaching an efficacy level of above 90% at both application rates. Considering the low crop damage of **7c** and **13b**, their strong control of sensitive and resistant lolium species emphasizes their potential as lead structures to identify tailored solutions for sustainable lolium control in relevant countries (e.g., Australia). In line with good control of other grass weeds (APESV and BROTE, Figure 1), target compound **7b** showed the most promising spectrum of efficacy of all new test compounds, being on the same level or slightly better than **1**. Hence, 2,3-dihydro[1,3]thiazolo[4,5-*b*]pyridines represent a propitious class of herbicidal lead structures with the potential to control resistant grass weeds. To complement our investigations that focused on relevant monocotyledon weeds in wheat, including resistant species, we tested the three selected 2,3-dihydro[1,3]thiazolo[4,5-*b*]pyridines **7b**, **7c**, and **13b** against commercially relevant weeds in corn, e.g., crabgrass or red fingergrass (DIGSA) and goosegrass or crowsfoot grass (ELEIN), together with Johnson grass (SORHA) and broad-leaved signal grass (BRAPP). We used **1** and **5** as standards to assess the potential of the new lead structures. Both standards showed sufficient crop selectivity only at the lower application rate of 50 g/ha. [1,3]Thiazolo[4,5-*b*]pyridine **5** showed good control of all tested warm-season weeds, whilst **1** showed only insufficient control of BRAPP, ELEIN, and SORHA at the lower application rate of 50 g/ha. 2,3-Dihydro[1,3]thiazolo[4,5-*b*]pyridines **7b**, **7c**, and **13b** showed good crop selectivity at both application rates in our advanced preemergence greenhouse test, affording low damage in corn and only marginal damage in soy (Figure 2). However, **7c** showed a moderate damage of 20% in both crops at the higher application rate of 150 g/ha. Remarkably, 2,3-dihydro[1,3]thiazolo[4,5-*b*]pyridine **7b** afforded good efficacy at both dose rates against all five monocotyledon weeds, including BRAPP, ELEIN, and SORHA, which were not sufficiently controlled by **1**. Whilst the structurally closely related compounds **7b** and **5** showed comparable efficacy against all tested weeds, **7b** afforded considerably lower crop damage at the higher application rate in corn and at both application rates in soy compared to **5** (Figure 2). Furthermore, **7b**, **7c**, and **13b** provided full control of ELEIN, one of the most difficult turfgrass weeds to control in the tropics and warmer temperate zones, emphasizing the potential of novel FAT inhibitors to contribute to integrated weed and resistance management.

## Conclusion

The agrochemical work outlined herein covers a series of novel herbicidal lead structures that contain a 2,3-dihydro[1,3]thiazolo[4,5-*b*]pyridine unit as the essential structural feature, with all of them carrying an *o-*substituted phenyl group. Inspired by earlier work in our group focusing on substituted thiazolo[4,5-*b*]pyridines that showed promising inhibition of the plant-specific enzyme FAT, we explored the selective late-stage conversion into the corresponding 2,3-dihydro[1,3]thiazolo[4,5-*b*]pyridines via different reduction methods. Noteworthy, substituted 2,3-dihydro[1,3]thiazolo[4,5-*b*]pyridines had remained almost entirely unexplored prior to our investigations. Likewise, we identified an optimized BH_3_⋅NH_3_-mediated reduction involving tris(pentafluorophenyl)borane as a strong Lewis acid and subsequent treatment with formic acid as the most suitable conditions to prepare the desired 2,3-dihydro[1,3]thiazolo[4,5-*b*]pyridines. It is worth noting that this reduction proved to be thiazole-specific and gave access to a broad range of desired target compounds. Several substituted 2,3-dihydro[1,3]thiazolo[4,5-*b*]pyridines showed promising herbicidal activity against commercially important grass weeds in preemergence greenhouse tests in line with competitive application rates and hints for crop selectivity, particularly in wheat and soy. Furthermore, the new heterocyclic lead structures have the potential to mitigate and affect weeds that have become resistant towards commercial herbicides, such as resistant blackgrass (ALOMY_R, also known as slender foxtail or black twitch) and ryegrass (LOLSS_R). Remarkably, 2,3-dihydro[1,3]thiazolo[4,5-*b*]pyridine **7b** turned out to be superior to market standards (i.e., **1** and **2** in wheat) in terms of overall efficacy and resistance breaking potential. Halogen-free target compound **7c** also showed strong efficacy against commercially important weeds, in particular resistant ryegrass (LOLSS_R), combined with promising crop safety. In our view, these results underline that chemical innovation using isostere concepts and addressing unusual structural features is a useful tool to broaden the structural scope of modern agrochemical research and to address sustainability goals, e.g., overcoming herbicide resistance and meeting demanding environmental safety goals.

## Experimental

### Synthesis

**Representative procedure for the synthesis of 6-bromo-5-(2-fluorophenyl)-2,3-dihydro[1,3]thiazolo[4,5-*b*]pyridine (13b):** To a stirred mixture of 6-bromopyridin-2-amine (**8**, 10.00 g, 56.88 mmol, 1.00 equiv), 2-fluorophenylboronic acid (9.52 g, 65.98 mmol, 1.16 equiv), and Na_2_CO_3_ (12.06 g, 113.8 mmol, 2.00 equiv) in a mixture of 1,4-dioxane (80 mL) and water (80 mL) at room temperature was added Pd(dppf)Cl_2_ (1.67 g, 2.28 mmol, 0.04 equiv), and the mixture was stirred at 80 °C for 3 h. Thereafter, the reaction mixture was cooled to room temperature, diluted with water, and extracted thoroughly with ethyl acetate. The combined organic layer was washed with brine, dried over Na_2_SO_4_, filtered, and concentrated under reduced pressure. The remaining residue was purified via column chromatography (gradient ethyl acetate/hexane) to afford 6-(2-fluorophenyl)pyridin-2-ylamine (**9b**, 9.98 g, 93%). ^1^H NMR (400 MHz, CDCl_3_, δ) 7.92–7.88 (m, 1H), 7.53–7.49 (m, 1H), 7.36–7.31 (m, 1H), 7.25–7.20 (m, 1H), 7.16–7.10 (m, 1H), 6.50–6.48 (d, 1H), 4.53–4.44 (br s, 2H, NH_2_).

6-(2-Fluorophenyl)pyridin-2-ylamine (**9b**, 9.98 g, 52.49 mmol, 1.0 equiv) was dissolved in acetonitrile (140 mL) and cooled to 0 °C. Thereafter, *N*-bromosuccinimide (20.56 g, 115.49 mmol, 2.2 equiv) was added carefully. The reaction mixture was warmed to room temperature and stirred for 4 h. Subsequently, the reaction mixture was diluted with water, and the resulting solid was filtered off. The solid was washed thoroughly with water and dried to afford 3,5-dibromo-6-(2-fluorophenyl)pyridin-2-ylamine (**10b**, 17.62 g, 97%) as an orange solid. ^1^H NMR (400 MHz, CDCl_3_, δ) 7.92 (s, 1H), 7.44–7.33 (m, 2H), 7.25–7.20 (m, 1H), 7.17–7.12 (m, 1H), 5.07–4.98 (br s, 2H, NH_2_).

To a stirred solution of 3,5-dibromo-6-(2-fluorophenyl)pyridin-2-ylamine (**10b**, 17.62 g, 50.93 mmol, 1.0 equiv) in DMF (120 mL) at room temperature was added potassium *O*-ethyl dithiocarbonate (18.52 g, 112.04 mmol, 2.2 equiv). The resulting mixture was heated at reflux for 7 h. Thereafter, the reaction mixture was cooled to room temperature, poured onto ice water, and acidified with 2 N HCl. The obtained precipitate was filtered, washed with water, collected, and dried under reduced pressure to afford **11b** as thiol–thione tautomer consisting of 6-bromo-5-(2-fluorophenyl)[1,3]thiazolo[4,5-*b*]pyridine-2-thiol and 6-bromo-5-(2-fluorophenyl)[1,3]thiazolo[4,5-*b*]pyridine-2(3*H*)thione (17.20 g, 97%). ^1^H NMR (400 MHz, CDCl_3_, δ) 9.96 (br s, 1H), 8.01 (s, 1H), 7.50–7.44 (m, 1H), 7.41–7.38 (m, 1H), 7.29–7.25 (m, 1H), 7.19–7.15 (m, 1H).

The thiol–thione tautomer **11b** (14.55 g, 42.64 mmol, 1.0 equiv) was dissolved in acetic acid (200 mL), and iron powder (35.71 g, 639.61 mmol, 15 equiv) was carefully added in portions. The resulting reaction mixture was stirred at 100 °C for 10 h. After full conversion (indicated by LC–MS), the reaction mixture was cooled to 60 °C, and the iron powder was filtered off. The remaining solution was diluted with water, and the resulting precipitate was filtered, washed with water, and dried under reduced pressure. The remaining crude residue was redissolved in DCM, then water was added, followed by thorough extraction. The combined organic layer was dried over magnesium sulfate, filtered, and dried under reduced pressure. The remaining residue was purified via column chromatography (gradient ethyl acetate/hexane) to afford 6-bromo-5-(2-fluorophenyl)[1,3]thiazolo[4,5-*b*]pyridine (**12b**, 8.75 g, 63%). ^1^H NMR (400 MHz, CDCl_3_, δ) 9.32 (s, 1H), 8.64 (s, 1H), 7.54–7.45 (m, 2H), 7.31–7.27 (m, 1H), 7.21–7.16 (m, 1H).

6-Bromo-5-(2-fluorophenyl)[1,3]thiazolo[4,5-*b*]pyridine (**12b**, 1,000 mg, 3.07 mmol, 1.0 equiv) was dissolved in absolute toluene (10 mL) in an oven-dried round-bottom flask under argon, to which ammonia borane (285 mg, 9.20 mmol, 3.0 equiv) and B(C_6_F_5_)_3_ (79 mg, 0.15 mmol, 0.05 equiv) were added. The resulting reaction mixture was stirred at a temperature of 45 °C for 5 h and then concentrated under reduced pressure. The remaining residue was redissolved in acetonitrile, formic acid was added, and the reaction mixture was stirred at room temperature for 2 h. The phases were separated via phase separator, and the organic layer was concentrated under reduced pressure. The remaining crude product was purified via column chromatography (gradient ethyl acetate/hexane) to afford 6-bromo-5-(2-fluorophenyl)-2,3-dihydro[1,3]thiazolo[4,5-*b*]pyridine (**13b**, 596 mg, 59%). ^1^H NMR (600 MHz, CDCl_3_, δ) 4.56 (s, 2H), 6.88 (br s, 1H), 7.11–7.16 (m, 1H), 7.21 (td, *J* = 7.5; 1.1 Hz, 1H), 7.31 (s, 1H), 7.34 (td, *J* = 7.4; 1.9 Hz, 1H), 7.36–7.41 (m, 1H); ^13^C NMR (151 MHz, CDCl_3_, δ) 49.0 (CH_2_), 108.1 (C), 115.7 (CH), 115.9 (CH), 123.5 (C), 124.0 (CH), 127.7 (d, C), 130.4 (CH), 131.1 (CH), 146.2 (C), 158.7 (C), 160.3 (d, C); HRESIMS (*m*/*z*): [M + H]^+^ calcd for C_12_H_9_BrFN_2_S, 310.9671; found, 310.9654.

**5-(2-Fluorophenyl)-6-methyl-2,3-dihydro[1,3]thiazolo[4,5-*b*]pyridine (7b):** 6-Bromo-5-(2-fluorophenyl)[1,3]thiazolo[4,5-*b*]pyridine (**12b**, 1.88 g, 4.56 mmol, 1.0 equiv), methylboronic acid (1.13 g, 18.24 mmol, 4.0 equiv), potassium phosphate (1.94 g, 9.12 mmol, 2.0 equiv), palladium(II) acetate (103 mg, 0.46 mmol, 0.1 equiv), and 2-dicyclohexylphosphino-2’,6’-dimethoxybiphenyl (579 mg, 1.37 mmol, 0.3 equiv) were dissolved in absolute toluene (40 mL) in an oven-dried round-bottom flask under argon. The resulting reaction mixture was stirred at reflux for 3.5 h. After cooling to room temperature, water (80 mL) and toluene (20 mL) were added, followed by addition of ammonium pyrrolidinedithiocarbamate (328 mg, 2.00 mmol, 0.44 equiv). The reaction mixture was stirred for 1 h at room temperature, and the organic layer was washed with saturated sodium hydrogencarbonate solution, dried over magnesium sulfate, filtered, and concentrated under reduced pressure. The remaining residue was purified via column chromatography (gradient ethyl acetate/hexane) to afford 5-(2-fluorophenyl)-6-methyl[1,3]thiazolo[4,5-*b*]pyridine (**5**, 900 mg, 81%). ^1^H NMR (400 MHz, CDCl_3_, δ) 9.25 (s, 1H), 8.23 (s, 1H), 7.55–7.51 (m, 1H), 7.47–7.41 (m, 1H), 7.30–7.27 (m, 1H), 7.19–7.15 (m, 1H), 2.40 (s, 3H).

5-(2-Fluorophenyl)-6-methyl[1,3]thiazolo[4,5-*b*]pyridine (**5**, 320 mg, 1.23 mmol, 1.0 equiv) was dissolved in absolute toluene (10 mL) in an oven-dried round-bottom flask under argon, to which ammonia borane (114 mg, 3.69 mmol, 3.0 equiv) and B(C_6_F_5_)_3_ (32 mg, 0.06 mmol, 0.05 equiv) were added. The resulting reaction mixture was stirred at a temperature of 45 °C for 5 h and then concentrated under reduced pressure. The remaining residue was redissolved in acetonitrile, formic acid was added, and the reaction mixture was stirred at room temperature for further 45 min. The phases were separated via phase separator, and the organic layer was concentrated under reduced pressure. The remaining crude product was purified via column chromatography (gradient ethyl acetate/hexane) to afford 5-(2-fluorophenyl)-6-methyl-2,3-dihydro[1,3]thiazolo[4,5-*b]*pyridine (**7b**, 62%). ^1^H NMR (600 MHz, CDCl_3_, δ) 2.00 (s, 3H), 4.55 (s, 2H), 6.61 (br s, 1H), 7.07 (s, 1H), 7.09–7.12 (m, 1H), 7.19–7.21 (m, 1H), 7.32–7.36 (m, 2H); ^13^C NMR (151 MHz, CDCl_3_, δ) 17.96 (CH_3_), 17.99 (CH_3_), 48.8 (CH_2_), 115.6 (CH), 115.7 (CH), 121.0 (C), 121.8 (C), 124.15 (CH), 124.18 (CH), 129.59 (CH), 129.64 (CH), 130.2 (CH), 131.40 (CH), 131.43 (CH), 145.6 (C), 158.9 (C), 159.2 (C), 160.5 (C); HRESIMS (*m*/*z*): [M + H]^+^ calcd for C_13_H_12_FN_2_S, 247.0705; found, 247.0724.

**General procedure for the synthesis of N-acylated 6-methyl-2,3-dihydro[1,3]thiazolo[4,5-*b*]pyridines 16a**–**f:** The corresponding acyl chloride (0.31 mmol, 1.1 equiv) and triethylamine (0.09 mL, 0.63 mmol, 2.2 equiv) were added to a stirred solution of the corresponding 6-methyl-5-phenyl-2,3-dihydro[1,3]thiazolo[4,5-*b*]pyridine **7a**–**c** (0.28 mmol, 1.00 equiv) in absolute DCM (5 mL). The resulting reaction mixture was stirred at room temperature for 30–120 min, followed by dilution with DCM and water, and subsequent extraction and phase separation. The aqueous layer was thoroughly extracted with DCM, and the combined organic layer was dried over anhydrous MgSO_4_, filtered, and concentrated under reduced pressure. The remaining crude product was purified via column chromatography (gradient ethyl acetate/heptane) to afford the corresponding desired target compound **16a**–**f**, for example, 1-[5-(2-fluorophenyl)-6-methyl[1,3]thiazolo[4,5-*b*]pyridin-3(2*H*)-yl]ethanone (**16c**, 150 mg, 84%). ^1^H NMR (600 MHz, CDCl_3_, δ) 2.16 (d, *J* = 2.2 Hz, 3H), 2.61 (s, 2H), 5.32 (s, 2H), 7.12–7.15 (m, 1H), 7.21–7.24 (m, 1H), 7.34 (s, 1H), 7.37–7.49 (m, 2H); ^13^C NMR (151 MHz, CDCl_3_, δ) 18.41 (CH_3_), 18.44 (CH_3_), 24.7 (CH_3_), 49.3 (CH_2_), 115.7 (CH), 115.8 (CH), 124.09 (CH), 124.12 (CH), 125.2 (C), 127.8 (C), 129.9 (CH), 130.0 (CH), 131.37 (CH), 131.40 (CH), 132.1 (CH), 147.0 (C), 149.6 (C), 158.9 (C), 160.6 (C), 170.6 (C); HRESIMS (*m*/*z*): [M + H]^+^ calcd for C_15_H_14_FN_2_OS, 289.0811; found, 289.0806.

### Biology and biochemistry

**In vivo greenhouse screening:** Seeds of mono- and dicotyledonous weed plants and crop plants were sown in plastic or organic planting pots in sandy loam and covered with soil (replicates: *n* = 10, i.e., 10 monocotyledonous weed seeds were grown per pot or *n* = 5, i.e., 5 dicotyledonous weed seeds were grown per pot). The 2,3-dihydro[1,3]thiazolo[4,5-*b*]pyridines described above (e.g., **7a**–**c**, **13a**–**c**, **14a**–**c**, **16a**–**f**, and **17a**–**b**), formulated in the form of wettable powder (WP) , were applied to the surface of the covering soil as aqueous suspension or emulsion, with the addition of 0.5% of an additive at an application rate of 600 L of water/ha (converted). Following treatment, the pots were placed in a greenhouse and kept under optimum growth conditions for the test plants. The test plants were placed in the greenhouse for ca. three weeks under optimum growth conditions, and then the effect of the preparations was assessed visually in comparison to untreated control plants (herbicidal effect: 100% = plants died off, 0% = as untreated control plants). Efficacy values were given based on a rating scale by final visual experts’ assessment of green mass, i.e., 5 = ≥90% inhibition, 4 = 70–89% inhibition, 3 = 50–69% inhibition, 2 = 40–49% inhibition, 1 = 21–39% inhibition, and — = <20% inhibition. Advanced screening was carried out with or as emulsifiable concentrate formulations, three replicate pots, and a standardized number of seeds per pot depending on the plant species (10 seeds for corn and wheat spectrum). The harmful plants and crops used in greenhouse tests were the following species: *Alopecurus myosuroides* (ALOMY), resistant *Alopecurus myosuroides* (ALOMY_R, origin: Germany), *Apera spica-venti* (APESV), *Brachiaria platyphylla* (BRAPP), *Bromus tectorum* (BROTE), *Echinochloa crus-galli* (ECHCG), *Digitaria sanguinalis* (DIGSA), *Eleusine indica* (ELEIN), *Glycine max* (GLXMA), *Lolium rigidum* (LOLRI), *Lolium sp.* (LOLSS), resistant *Lolium sp.* (LOLSS_R, origin: France), *Poa annua* (POAAN), *Setaria viridis* (SETVI), *Sorghum halepense* (SORHA), *Triticum aestivum* (TRZAS), and *Zea mays* (ZEAMX).

***Lp*FAT A expression and purification:** The *fat a03* gene from *Lemna paucicostata*, in which the N-terminal amino acids representing the chloroplast transit peptide were replaced by an N-terminal 6xHis-tag, was cloned into a pET24 vector [3]. The *Lp*FAT A protein was expressed in *E. coli* BL21Star(DE3) cells. 5 mL of an overnight culture of *E. coli* cells grown in LB medium with 100 µg/mL carbenicillin were used to inoculate 0.5 L of autoinduction medium containing 100 µg/mL carbenicillin [28]. The bacteria were grown at 37 °C and 120 rpm for about 4.5 h to reach OD_600_ = 0.6 and then further cultivated at 21 °C overnight. The bacteria were harvested by centrifugation (20 min, 6,000*g*) and stored frozen at −80 °C. *Lp*FAT A protein was purified using the Ni-NTA Fast Start Kit (Qiagen GmbH, Germany) according to the instructions of the manufacturer. Active fractions were pooled together, and the buffer was exchanged into 25 mM potassium phosphate buffer pH 7.3 containing 10% glycerol with PD10 columns (GE Healthcare). Aliquots of the protein solution were frozen in liquid nitrogen and stored at −80 °C.

***Lp*FAT A fluorescence polarization assay:** Fluorescence polarization (FP) competition assays were performed at room temperature in black 96-well microtiter plates (Greiner, Catalog No. 655900). The assay mixture contained 25 mM potassium phosphate buffer pH 7.3, 200 mM NaCl, 0.01% Triton X-100, 2 nM fluorescent tracer, 0.4 µg of purified *Lp*FAT A protein and different amounts of the test compound in a total volume of 100 µL. FP was measured with a BMG CLARIOstar microtiter plate reader using the FP filter set for fluorescein (Ex 482-16, Em 530-40, LP504). FP is the difference between wells containing *Lp*FAT A and wells containing only tracer. The pI_50_ values were calculated from plots of inhibition values vs test compound concentration using Model 205 of the ID Business Solutions Ltd Xlfit software suite. The FAT A binding fluorescent tracer was synthesized from (2*S*,4*S*)-4-[(2,6-difluorophenyl)methoxymethyl]-4-ethyl-2-methyl-*N*-(prop-2-ynylcarbamoyl)-1,3-dioxolane-2-carboxamide [3] and FAM azide, 5-isomer (Broadpharm BP-22544, San Diego, CA) by click chemistry [29] and was purified by flash column chromatography on silica gel.

## Supporting Information

File 1 General synthetic procedures, characterization of all target compounds, methods for biological and biochemical testing, and scans of ^1^H and ^13^C NMR spectra of the new 2,3-dihydro[1,3]thiazolo[4,5-b]pyridines.
