# Supplementary material for: Synthesis and biological profile of 2,3-dihydro[1,3]thiazolo[4,5-b]pyridines, a novel class of acyl-ACP thioesterase inhibitors
Source: Beilstein J Org Chem. 2024 Mar 1;20:540–51. doi: 10.3762/bjoc.20.46 (PMC10910475; doi:10.3762/bjoc.20.46)
Supplement: File 1 — General synthetic procedures, characterization of all target compounds, methods for biological and biochemical testing, and scans of 1H and 13C NMR spectra of the new 2,3-dihydro[1,3]thiazolo[4,5-b]pyridines. [file Beilstein_J_Org_Chem-20-540-s001.pdf]

BEILSTEIN JOURNAL OF ORGANIC CHEMISTRY

Supporting Information

for

Synthesis and biological profile of 2,3-dihydro[1,3]thiazolo[4,5-*b*]pyridines, a novel class of acyl-ACP thioesterase inhibitors

Jens Frackenpohl, David M. Barber, Guido Bojack, Birgit Bollenbach-Wahl, Ralf Braun, Rahel Getachew, Sabine Hohmann, Kwang-Yoon Ko, Karoline Kurowski, Bernd Laber, Rebecca L. Mattison, Thomas Müller, Anna M. Reingruber, Dirk Schmutzler and Andrea Svejda

Beilstein J. Org. Chem. **2024,** *20,* 540–551. doi:10.3762/bjoc.20.46

General synthetic procedures, characterization of all target compounds, methods for biological and biochemical testing, and scans of ^{1}H and ^{13}C NMR spectra of the new 2,3-dihydro[1,3]thiazolo[4,5-b]pyridines

License and Terms: This is a supporting information file under the terms of the Creative Commons Attribution License (https://creativecommons.org/licenses/by/4.0). Please note that the reuse, redistribution and reproduction in particular requires that the author(s) and source are credited and that individual graphics may be subject to special legal provisions.
The license is subject to the *Beilstein Journal of Organic Chemistry* terms and conditions: (https://www.beilstein-journals.org/bjoc/terms)

1. Chemistry S2
1.1. General information S2
1.2. General synthetic procedures and characterization S2
1.2.1. General procedure for the synthesis of 6-bromo-5-2,3-dihydro[1,3]thiazolo[4,5-*b*]pyridines **13a**–**c** S2
1.2.2. Characterization of 6-bromo-5-2,3-dihydro[1,3]thiazolo[4,5-*b*]pyridines **13a**–**c** S3
1.2.3. General procedure for the synthesis of substituted 6-methyl-2,3-dihydro[1,3]thiazolo[4,5-*b*]pyridines **7a**–**c** S20
1.2.4. Characterization of substituted 6-methyl-2,3-dihydro[1,3]thiazolo[4,5-*b*]pyridines **7a**–**c** S20
1.2.5. General procedure for the synthesis of N-acylated 6-bromo-2,3-dihydro[1,3]thiazolo[4,5-*b*]pyridines **14a**–**c** S30
1.2.6. Characterization of N-acylated 6-bromo-2,3-dihydro[1,3]thiazolo[4,5-*b*]pyridines **14a**–**c** S30
1.2.7. General procedure for the synthesis of N-acylated 6-methyl-2,3-dihydro[1,3]thiazolo[4,5-*b*]pyridines **16a**–**f** S39
1.2.8. Characterization of N-acylated 6-methyl-2,3-dihydro[1,3]thiazolo[4,5-*b*]pyridines **16a**–**f** S39
1.2.9. Characterization of borylated compounds **17a**–**e** and side product **17f** S57

2. Biology and biochemistry S67
2.1. In vivo greenhouse screening S67
2.2. Biochemistry S68
2.2.1. *Lp*FAT A expression and purification S68
2.2.2. *Lp*FAT A fluorescence polarization assay S68

3. References S68

1. Chemistry

1.1. General information

All reagent-grade solvents and chemicals were purchased from standard commercial suppliers and used without further purification. All nonaqueous reactions were carried out under anhydrous conditions using dry solvents. Reactions were monitored by LC–MS or TLC carried out on 0.25 mm silica gel plates (60F-254). TLC plates were visualized using UV light. Yields refer to spectroscopically pure compounds unless otherwise stated. Flash column chromatography was carried out with a Biotage Isolera™ using CHROMABOND® Flash SiOH (Macherey-Nagel) columns in sizes ranging from 15 g to 120 g. The ^{1}H NMR, ^{13}C NMR and ^{19}F NMR spectroscopy data that are reported for the chemical examples described (400 and 600 MHz for ^{1}H NMR and 150 MHz for ^{13}C NMR and 375 MHz for ^{19}F NMR, solvent: $CDCl_3$, CD_3OD or d_6-DMSO, internal standard: tetramethylsilane δ = 0.00 ppm), were recorded using a Bruker AVII spectrometer with a Bruker TBI-probe, and the signals listed have the following meanings: br = broad; s = singlet, d = doublet, t = triplet, dd = doublet of doublets, ddd = doublet of a doublet of doublets, m = multiplet, q = quartet, quint = quintet, sext = sextet, sept = septet, dq = doublet of quartets, dt = doublet of triplets. The abbreviations used for chemical groups are defined as follows: Me = CH_3, Et = CH_2CH_3, *t*-Hex = $C(CH_3)_2CH(CH_3)_2$, *t*-Bu = $C(CH_3)_3$, *n*-Bu = unbranched butyl, *n*-Pr = unbranched propyl, c-Hex = cyclohexyl, *Ar*H = aromatic hydrogen, *Het*H = heteroaromatic hydrogen. High resolution mass spectrometry (HRMS) was conducted using a Waters spectrometer with Q-ToF Premiers using electron spray ionization (ESI).

1.2. General synthetic procedures and characterization

1.2.1. General procedure for the synthesis of 6-bromo-5-2,3-dihydro[1,3]thiazolo[4,5-*b*]pyridines 13a–c

To a stirred mixture of 6-bromopyridin-2-amine (**8**, 1.00 equiv), a phenylboronic acid (1.16 equiv), and Na_2CO_3 (2.00 equiv) in a mixture of 1,4-dioxane and water (1:1), Pd(dppf)Cl_2 (0.04 equiv) were added and stirred together at room temperature, before the mixture was stirred at 80 °C for 3 h. Thereafter, the reaction mixture was cooled to room temperature, diluted with water and extracted thoroughly with ethyl acetate. The combined organic layers were washed with brine, dried over anhydrous Na_2SO_4, filtered and concentrated under reduced pressure. The remaining residue was purified via column chromatography (gradient ethyl acetate/heptane) to afford the desired 6-phenylpyridin-2-amine **9**. The corresponding 6-phenylpyridin-2-amine **9** (1.0 equiv) was dissolved in acetonitrile and cooled to 0 °C. Thereafter, *N*-bromosuccinimide (2.2 equiv) was added carefully. The reaction mixture was warmed to room temperature and stirred for 4 h. Subsequently, the reaction mixture was diluted with water and the resulting solid was filtered off. The solid was washed thoroughly with water and dried to afford the corresponding 3,5-dibromo-6-phenylpyridin-2-amine **10**. To a stirred solution of a 3,5-dibromo-6-phenylpyridin-2-amine **10** (1.0 equiv) in *N*,*N*-dimethylformamide was added potassium *O*-ethyl dithiocarbonate (2.2 equiv) under an argon atmosphere at room temperature. The resulting mixture was heated under reflux conditions for 7 h. Thereafter, the reaction mixture was cooled to room temperature, poured onto ice water and acidified carefully with 2 N HCl. The obtained precipitate was filtered off, washed with water, collected and dried under reduced pressure to afford the corresponding 6-bromo-5-phenyl[1,3]thiazolo[4,5-b]pyridine-2-thiol and 6-bromo-5-(2-fluorophenyl)[1,3]thiazolo[4,5-*b*]pyridine-2(3*H*)thione thiol–thione tautomer **11**. As IUPAC naming tools have afforded thiols as preferred names we have opted to use the thiol naming throughout the Supporting Information. However, several spectra indicate that the thiones are the preferred tautomer in the $CDCl_3$ solution when

measuring the NMR spectra. In the next step, the corresponding 6-bromo-5-phenyl[1,3]thiazolo[4,5-b]pyridine-2-thiol **11** (1.0 equiv) was dissolved in acetic acid, and iron powder (15 equiv) was added carefully in portions. The resulting reaction mixture was stirred at a temperature of 100 °C for 10 h. After full conversion (indicated by LC–MS), the reaction mixture was cooled to 60 °C, and the iron powder was filtered off. The remaining solution was diluted with water, and the resulting precipitate was filtered, washed with water and dried under reduced pressure. The remaining crude residue was redissolved in dichloromethane, then water was added, followed by thorough extraction using dichloromethane. The combined organic layers were dried over anhydrous $MgSO_4$, filtered and dried under reduced pressure. The remaining residue was purified via column chromatography (gradient ethyl acetate/heptane) to afford the corresponding 6-bromo-5-phenyl[1,3]thiazolo[4,5-*b*]pyridine **12**. The 6-bromo-5-phenyl[1,3]thiazolo[4,5-*b*]pyridine **12** (3.07 mmol, 1.0 equiv) was dissolved in absolute toluene (10 mL) in a flame-dried round-bottom flask under argon. Ammonia borane (285 mg, 9.20 mmol, 3.0 equiv) and $B(C_6F_5)_3$ (79 mg, 0.15 mmol, 0.05 equiv) were added to the solution and the resulting reaction mixture was stirred at a temperature of 45 °C for 5 h and concentrated thereafter under reduced pressure. The remaining residue was redissolved in acetonitrile, formic acid was added, and the reaction mixture was stirred at room temperature for 2 h. The phases were separated via phase separator and the organic layer was concentrated under reduced pressure. The remaining crude product was purified via column chromatography (gradient ethyl acetate/hexane) to afford the desired substituted 6-bromo-2,3-dihydro[1,3]thiazolo[4,5-*b*]pyridine **13a–c**.

1.2.2. Characterization of 6-bromo-5-2,3-dihydro[1,3]thiazolo[4,5-b]pyridines 13a–c

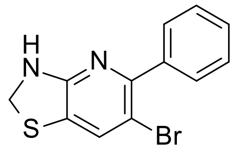

13a

6-Bromo-5-phenyl-2,3-dihydro[1,3]thiazolo[4,5-*b*]pyridine (**13a**, 700 mg, 70%). ^{1}H NMR (600 MHz, $CDCl_3$): δ 4.56 (s, 2H), 6.47-6.53 (m, 1H), 7.32-7.34 (m, 1H), 7.35-7.39 (m, 1H), 7.40-7.44 (m, 2H), 7.52-7.55 (m, 2H) ppm; ^{13}C NMR ($CDCl_3$, 151 MHz): δ 49.0 (CH_2), 106.5 (C), 122.3 (C), 128.0 (CH), 128.2 (CH), 129.2 (CH), 131.8 (CH), 139.7 (C), 150.9 (C), 159.9 (C) ppm; HRMS (ESI, m/z): calcd. for $C_{12}H_{10}N_2SBr$, 292.9735 $[M+H]^+$; found 292.9748.

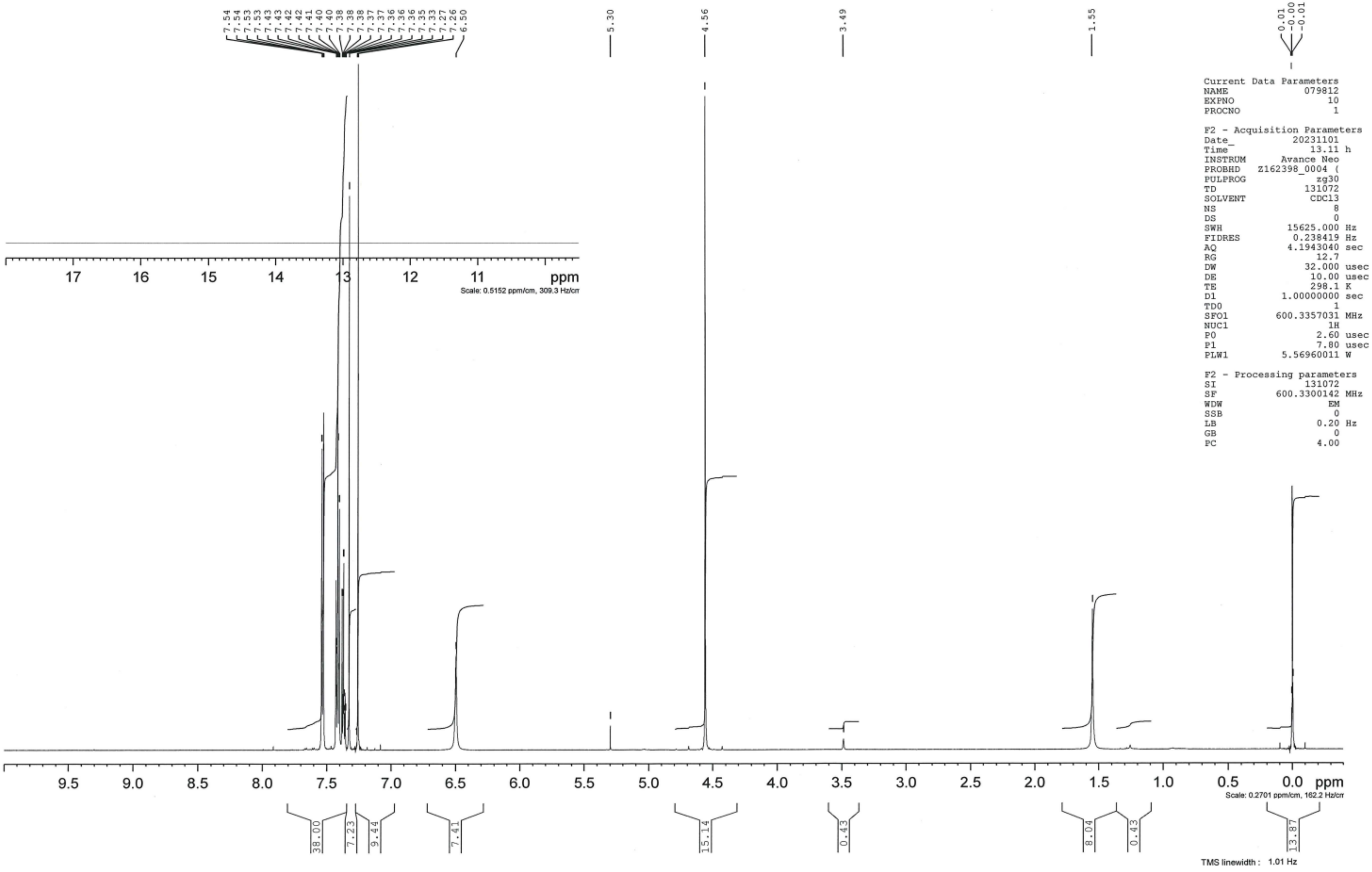
7.54 7.54 7.53 7.53 7.43 7.43 7.42 7.42 7.41 7.40 7.40 7.38 7.38 7.38 7.37 7.37 7.36 7.36 7.36 7.35 7.33 7.27 7.26 6.50
5.30
4.56
3.49
1.55
0.01 -0.00 -0.01
Current Data Parameters
NAME 079812
EXPNO 10
PROCNO 1
F2 - Acquisition Parameters
Date_ 20231101
Time_ 13.11 h
INSTRUM Avance Neo
PROBHD Z162398_0004 (
PULPROG zg30
TD 131072
SOLVENT CDCl3
NS 8
DS 0
SWH 15625.000 Hz
FIDRES 0.238419 Hz
AQ 4.1943040 sec
RG 12.7
DW 32.000 usec
DE 10.00 usec
TE 298.1 K
D1 1.00000000 sec
TD0 1
SFO1 600.3357031 MHz
NUC1 1H
P0 2.60 usec
P1 7.80 usec
PLW1 5.56960011 W
F2 - Processing parameters
SI 131072
SF 600.3300142 MHz
WDW EM
SSB 0
LB 0.20 Hz
GB 0
PC 4.00
17 16 15 14 13 12 11 ppm
Scale: 0.5152 ppm/cm, 309.3 Hz/cm
9.5 9.0 8.5 8.0 7.5 7.0 6.5 6.0 5.5 5.0 4.5 4.0 3.5 3.0 2.5 2.0 1.5 1.0 0.5 0.0 ppm
Scale: 0.2701 ppm/cm, 162.2 Hz/cm
38.00 7.23 9.44 7.41 15.14 0.43 8.04 0.43 13.87
TMS linewidth : 1.01 Hz

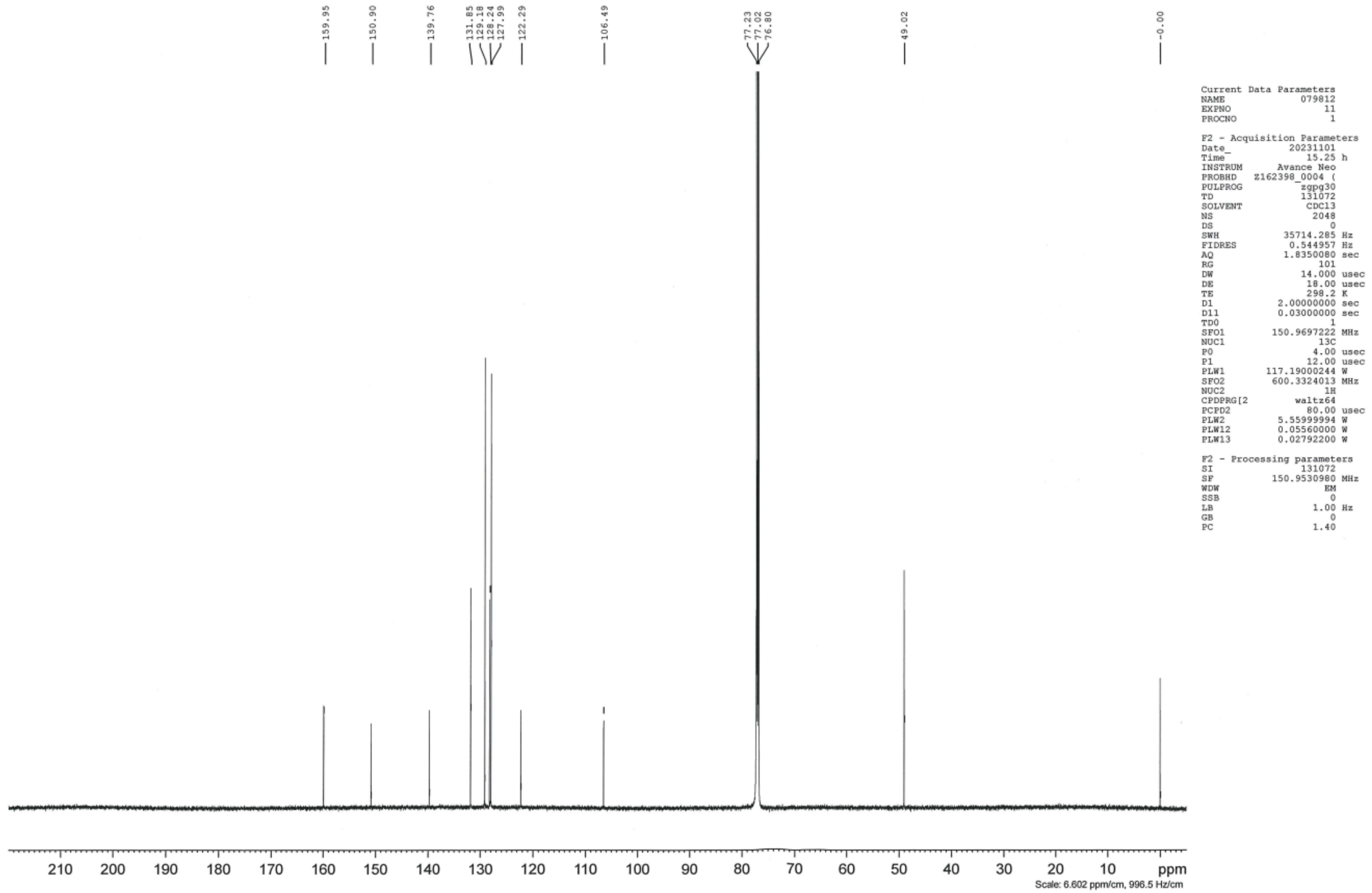
159.95
150.90
139.76
131.85
129.18
128.24
127.99
122.29
106.49
77.23
77.02
76.80
49.02
-0.00
210 200 190 180 170 160 150 140 130 120 110 100 90 80 70 60 50 40 30 20 10 ppm
Scale: 6.602 ppm/cm, 996.5 Hz/cm
Current Data Parameters
NAME 079812
EXPNO 11
PROCNO 1
F2 - Acquisition Parameters
Date_ 20231101
Time 15.25 h
INSTRUM Avance Neo
PROBHD Z162398_0004 (
PULPROG zgpg30
TD 131072
SOLVENT CDCl3
NS 2048
DS 0
SWH 35714.285 Hz
FIDRES 0.544957 Hz
AQ 1.8350080 sec
RG 101
DW 14.000 usec
DE 18.00 usec
TE 298.2 K
D1 2.00000000 sec
D11 0.03000000 sec
TD0 1
SFO1 150.9697222 MHz
NUC1 13C
P0 4.00 usec
P1 12.00 usec
PLW1 117.19000244 W
SFO2 600.3324013 MHz
NUC2 1H
CPDPRG[2 waltz64
PCPD2 80.00 usec
PLW2 5.55999994 W
PLW12 0.05560000 W
PLW13 0.02792200 W
F2 - Processing parameters
SI 131072
SF 150.9530980 MHz
WDW EM
SSB 0
LB 1.00 Hz
GB 0
PC 1.40

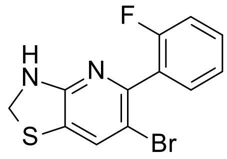

13b

Representative procedure for the synthesis of 6-bromo-5-(2-fluorophenyl)-2,3-dihydro[1,3]thiazolo[4,5-*b*]pyridine (13b): To a stirred mixture of 6-bromopyridin-2-amine (**8**, 10.00 g, 56.88 mmol, 1.00 equiv), 2-fluorophenylboronic acid (9.52 g, 65.98 mmol, 1.16 equiv), and Na_2CO_3 (12.06 g, 113.8 mmol, 2.00 equiv) in a mixture of 1,4-dioxane (80 mL) and water (80 mL) at room temperature was added Pd(dppf)Cl_2 (1.67 g, 2.28 mmol, 0.04 equiv), and the mixture was stirred at 80 °C for 3 h. Thereafter, the reaction mixture was cooled to room temperature, diluted with water, and extracted thoroughly with ethyl acetate. The combined organic layer was washed with brine, dried over Na_2SO_4, filtered, and concentrated under reduced pressure. The remaining residue was purified via column chromatography (gradient ethyl acetate/hexane) to afford 6-(2-fluorophenyl)pyridin-2-ylamine (**9b**, 9.98 g, 93%). ^{1}H NMR (400 MHz, $CDCl_3$): δ_H 7.92-7.88 (m, 1H), 7.53-7.49 (m, 1H), 7.36-7.31 (m, 1H), 7.25-7.20 (m, 1H), 7.16-7.10 (m, 1H), 6.50-6.48 (d, 1H), 4.53-4.44 (br. s, 2H, NH_2).

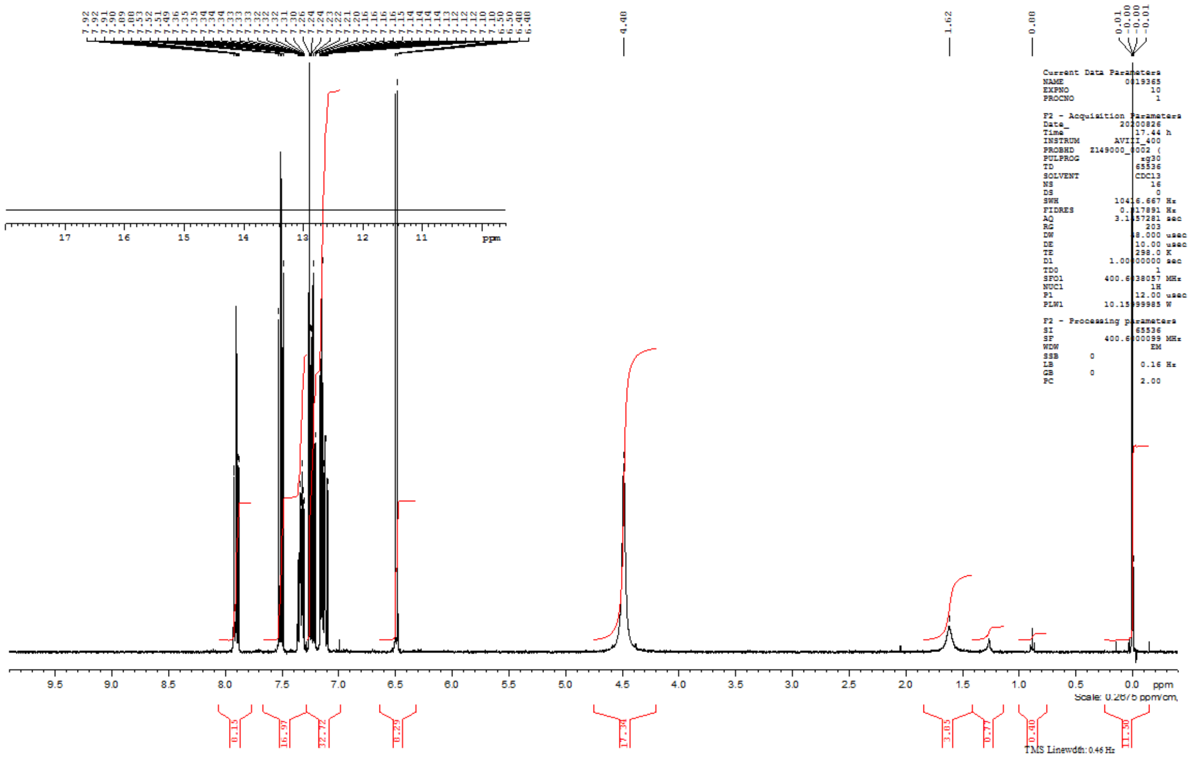

6-(2-Fluorophenyl)pyridin-2-ylamine (**9b**, 9.98 g, 52.49 mmol, 1.0 equiv) was dissolved in acetonitrile (140 mL) and cooled to 0 °C. Thereafter, *N*-bromosuccinimide (20.56 g, 115.49 mmol, 2.2 equiv) was added carefully. The reaction mixture was warmed to room temperature and stirred for 4 h. Subsequently, the reaction mixture was diluted with water, and the resulting solid was filtered off. The

solid was washed thoroughly with water and dried to afford 3,5-dibromo-6-(2-fluorophenyl)pyridin-2-ylamine (**10b**, 17.62 g, 97%) as an orange solid. ^{1}H NMR (400 MHz, $CDCl_3$): δ_H 7.92 (s, 1H), 7.44-7.33 (m, 2H), 7.25-7.20 (m, 1H), 7.17-7.12 (m, 1H), 5.07-4.98 (br. s, 2H, NH_2).

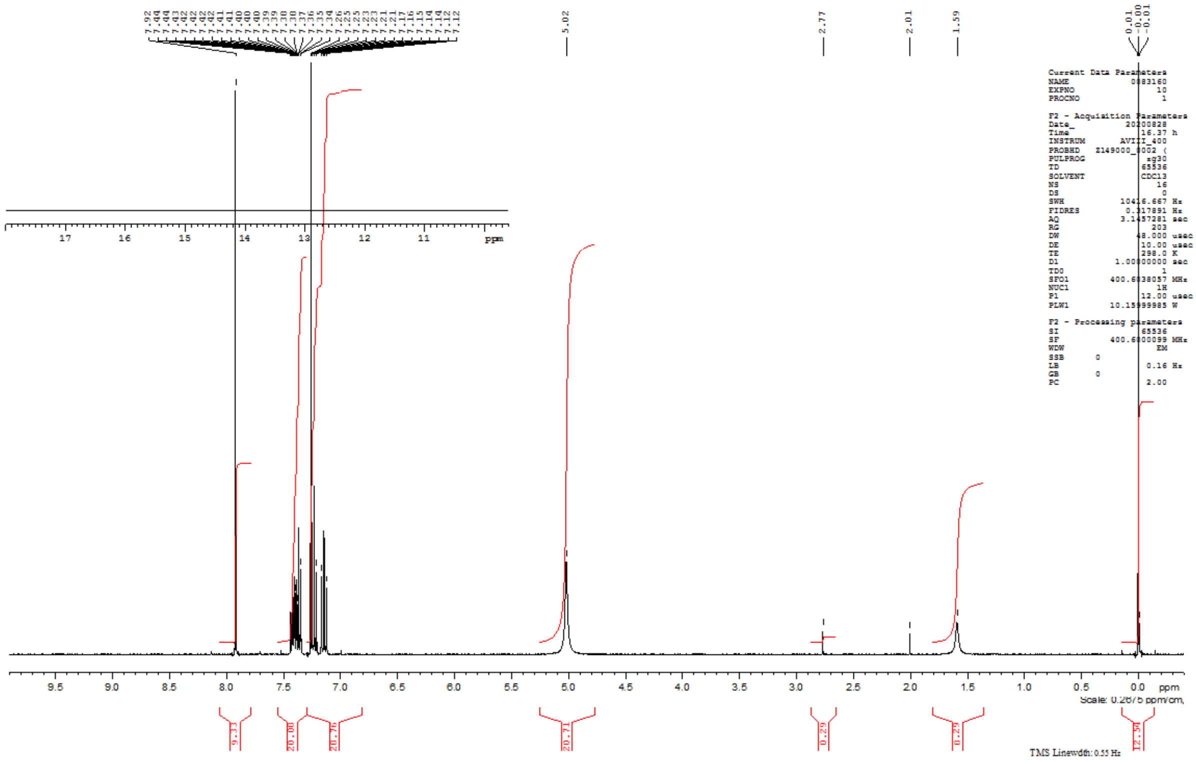

To a stirred solution of 3,5-dibromo-6-(2-fluorophenyl)pyridin-2-ylamine (**10b**, 17.62 g, 50.93 mmol, 1.0 equiv) in DMF (120 mL) at room temperature was added potassium *O*-ethyl dithiocarbonate (18.52 g, 112.04 mmol, 2.2 equiv). The resulting mixture was heated at reflux for 7 h. Thereafter, the reaction mixture was cooled to room temperature, poured onto ice water, and acidified with 2 N HCl. The obtained precipitate was filtered, washed with water, collected, and dried under reduced pressure to afford **11b** as thiol–thione tautomer consisting of 6-bromo-5-(2-fluorophenyl)[1,3]thiazolo[4,5-*b*]pyridine-2-thiol and 6-bromo-5-(2-fluorophenyl)[1,3]thiazolo[4,5-*b*]pyridine-2(3*H*)thione (17.20 g, 97%). ^{1}H NMR (400 MHz, $CDCl_3$): δ_H 9.96 (br. s, 1H), 8.01 (s, 1H), 7.50-7.44 (m, 1H), 7.41-7.38 (m, 1H), 7.29-7.25 (m, 1H), 7.19-7.15 (m, 1H).

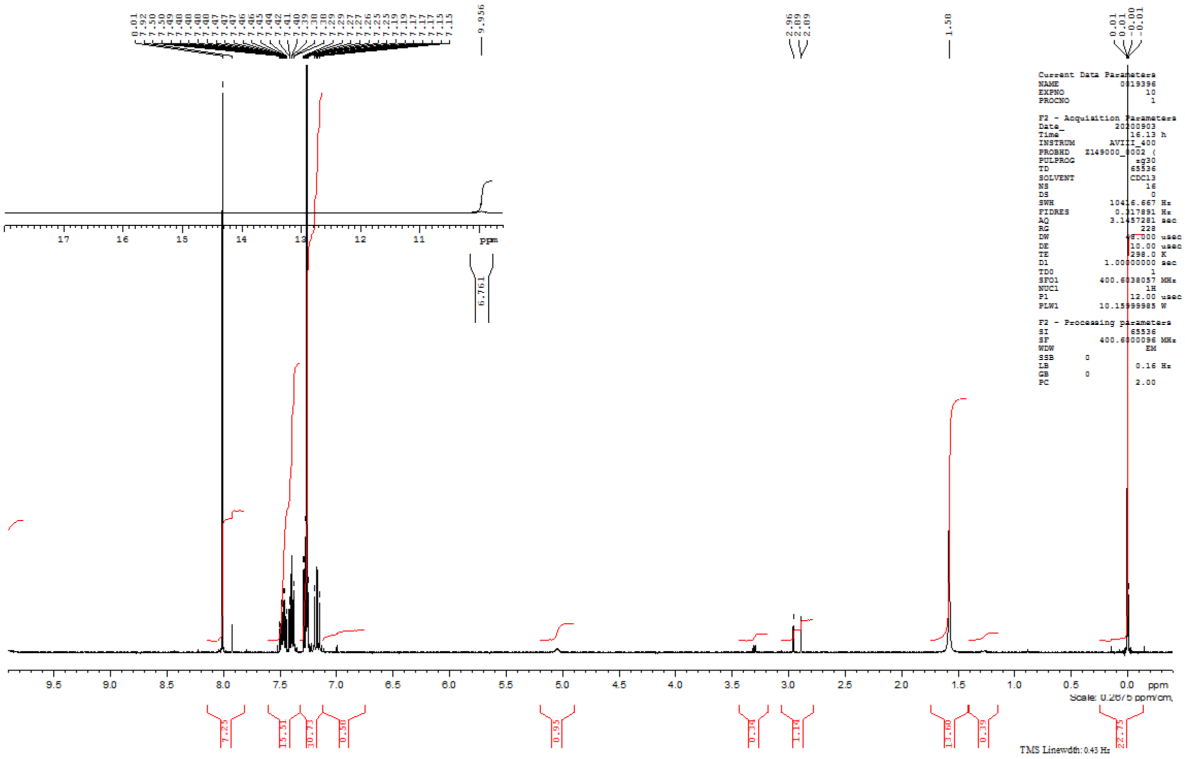

The thiol–thione tautomer **11b** (14.55 g, 42.64 mmol, 1.0 equiv) was dissolved in acetic acid (200 mL), and iron powder (35.71 g, 639.61 mmol, 15 equiv) was carefully added in portions. The resulting reaction mixture was stirred at 100 °C for 10 h. After full conversion (indicated by LC–MS), the reaction mixture was cooled to 60 °C, and the iron powder was filtered off. The remaining solution was diluted with water, and the resulting precipitate was filtered, washed with water, and dried under reduced pressure. The remaining crude residue was redissolved in DCM, then water was added, followed by thorough extraction. The combined organic layer was dried over magnesium sulfate, filtered, and dried under reduced pressure. The remaining residue was purified via column chromatography (gradient ethyl acetate/hexane) to afford 6-bromo-5-(2-fluorophenyl)[1,3]thiazolo[4,5-*b*]pyridine (**12b**, 8.75 g, 63%). ^{1}H NMR (400 MHz, $CDCl_3$): δ_H 9.32 (s, 1H), 8.64 (s, 1H), 7.54-7.45 (m, 2H), 7.31-7.27 (m, 1H), 7.21-7.16 (m, 1H).

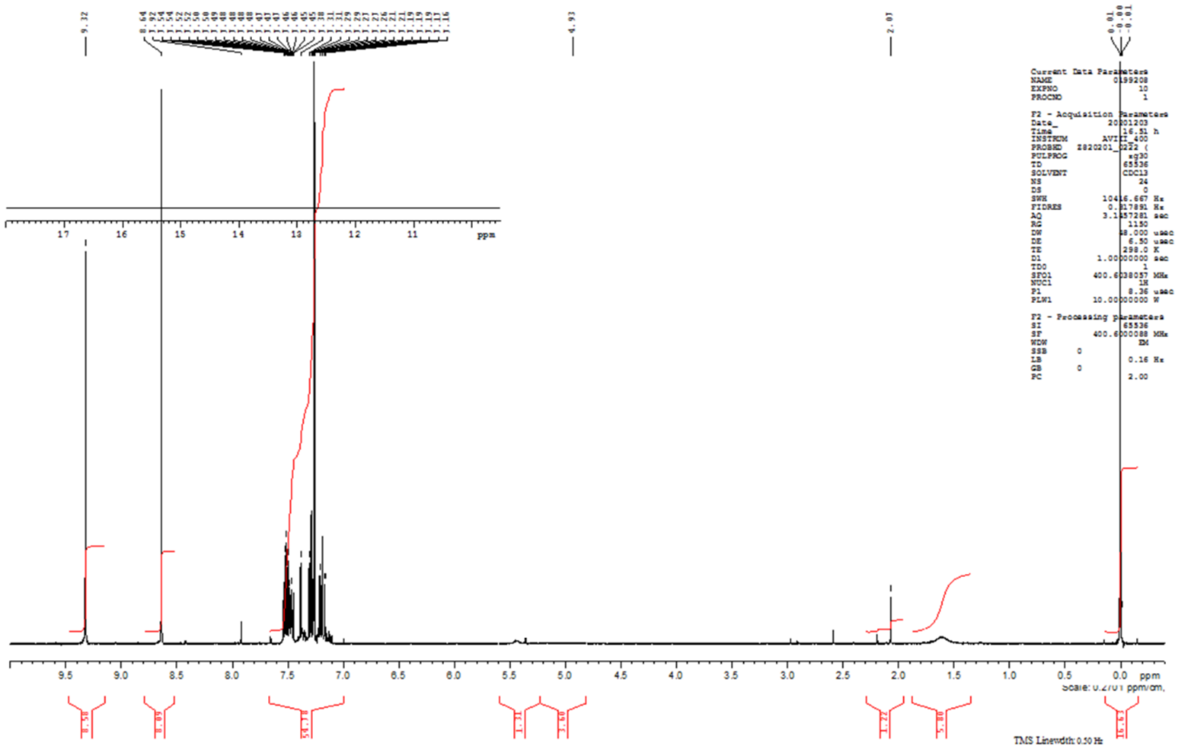

6-Bromo-5-(2-fluorophenyl)[1,3]thiazolo[4,5-*b*]pyridine (**12b**, 1,000 mg, 3.07 mmol, 1.0 equiv) was dissolved in absolute toluene (10 mL) in an oven-dried round-bottom flask under argon, to which ammonia borane (285 mg, 9.20 mmol, 3.0 equiv) and $B(C_6F_5)_3$ (79 mg, 0.15 mmol, 0.05 equiv) were added. The resulting reaction mixture was stirred at a temperature of 45 °C for 5 h and then concentrated under reduced pressure. The remaining residue was redissolved in acetonitrile, formic acid was added, and the reaction mixture was stirred at room temperature for 2 h. The phases were separated via phase separator, and the organic layer was concentrated under reduced pressure. The remaining crude product was purified via column chromatography (gradient ethyl acetate/hexane) to afford 6-bromo-5-(2-fluorophenyl)-2,3-dihydro[1,3]thiazolo[4,5-*b*]pyridine (**13b**, 596 mg, 59%). ^{1}H NMR (600 MHz, $CDCl_3$): δ 4.56 (s, 2H), 6.88 (brs, 1H), 7.11–7.16 (m, 1H), 7.21 (td, J = 7.5, 1.1 Hz, 1H), 7.31 (s, 1H), 7.34 (td, J = 7.4, 1.9 Hz, 1H), 7.36–7.41 (m, 1H) ppm; ^{13}C NMR ($CDCl_3$, 151 MHz): δ 49.0 (CH_2), 108.1 (C), 115.7 (CH), 115.9 (CH), 123.5 (C), 124.0 (CH), 127.7 (d, C), 130.4 (CH), 131.1 (CH), 146.2 (C). 158.7 (C), 160.3 (d, C) ppm; HRMS (ESI, m/z): calcd. $C_{12}H_9BrFN_2S$, 310.9671 $[M+H]^+$; found 310.9654.

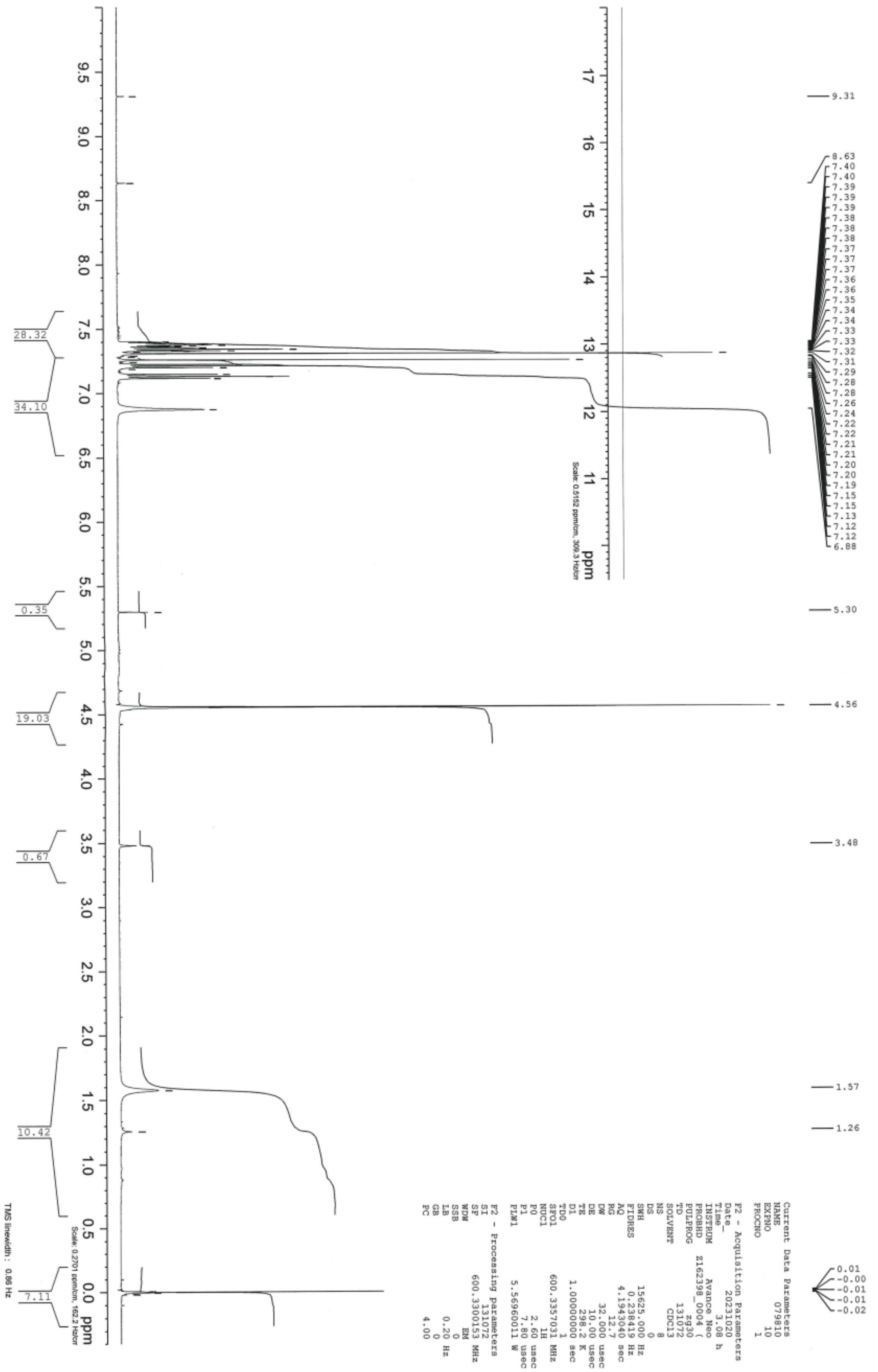

Current Data Parameters
NAME 079810
EXPNO 10
PROCNO 1
F2 - Acquisition Parameters
Date_ 20231020
Time 3.08 h
INSTRUM Avance Neo
PROBHD Z162398_0004 (
PULPROG zg30
TD 131072
SOLVENT CDCl3
NS 8
DS 0
SWH 15625.000 Hz
FIDRES 0.238419 Hz
AQ 4.1943040 sec
RG 12.7
DW 32.000 usec
DE 10.00 usec
TE 298.2 K
D1 1.0000000 sec
TD0 1
SFO1 600.3357031 MHz
NUC1 1H
P0 2.60 usec
P1 7.80 usec
PLW1 5.56960011 W
F2 - Processing parameters
SI 131072
SF 600.3300153 MHz
WDW EM
SSB 0
LB 0.20 Hz
GB 0
PC 4.00
ppm
Scale: 0.5152 ppm/cm, 309.3 Hz/cm
Scale: 0.2701 ppm/cm, 162.2 Hz/cm
TMS linewidth: 0.86 Hz

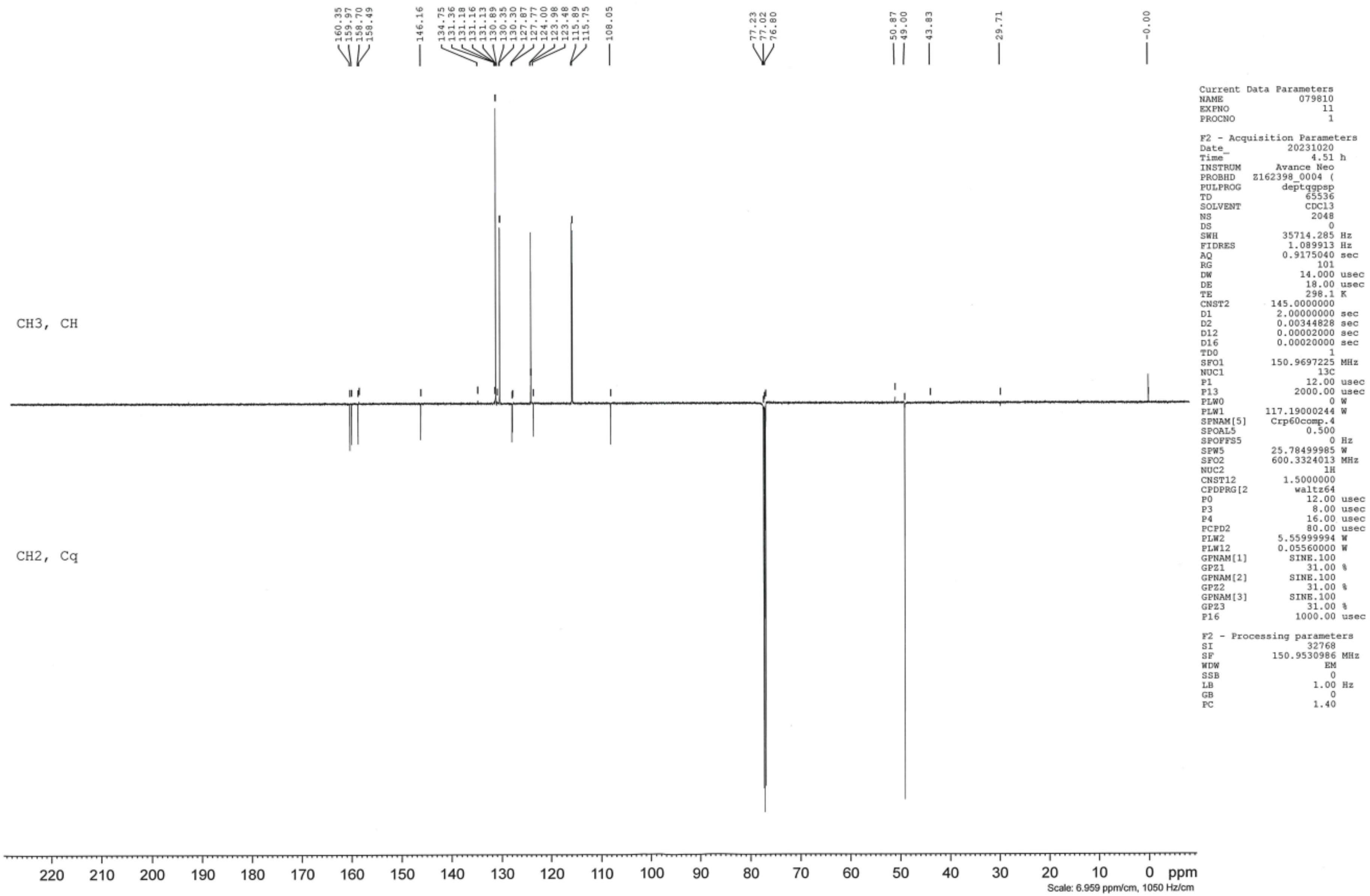
160.35
159.97
158.70
158.49
146.16
134.75
131.36
131.18
131.16
131.13
130.89
130.35
130.30
127.87
127.77
124.00
123.98
123.48
115.89
115.75
108.05
77.23
77.02
76.80
50.87
49.00
43.83
29.71
-0.00
CH3, CH
CH2, Cq
220 210 200 190 180 170 160 150 140 130 120 110 100 90 80 70 60 50 40 30 20 10 0 ppm
Scale: 6.959 ppm/cm, 1050 Hz/cm
Current Data Parameters
NAME 079810
EXPNO 11
PROCNO 1
F2 - Acquisition Parameters
Date_ 20231020
Time 4.51 h
INSTRUM Avance Neo
PROBHD Z162398_0004 (
PULPROG deptqgpsp
TD 65536
SOLVENT CDCl3
NS 2048
DS 0
SWH 35714.285 Hz
FIDRES 1.089913 Hz
AQ 0.9175040 sec
RG 101
DW 14.000 usec
DE 18.00 usec
TE 298.1 K
CNST2 145.0000000
D1 2.00000000 sec
D2 0.00344828 sec
D12 0.00002000 sec
D16 0.00020000 sec
TD0 1
SFO1 150.9697225 MHz
NUC1 13C
P1 12.00 usec
P13 2000.00 usec
PLW0 0 W
PLW1 117.19000244 W
SPNAM[5] Crp60comp.4
SPOAL5 0.500
SPOFFS5 0 Hz
SPW5 25.78499985 W
SFO2 600.3324013 MHz
NUC2 1H
CNST12 1.5000000
CPDPRG[2 waltz64
P0 12.00 usec
P3 8.00 usec
P4 16.00 usec
PCPD2 80.00 usec
PLW2 5.55999994 W
PLW12 0.05560000 W
GPNAM[1] SINE.100
GPZ1 31.00 %
GPNAM[2] SINE.100
GPZ2 31.00 %
GPNAM[3] SINE.100
GPZ3 31.00 %
P16 1000.00 usec
F2 - Processing parameters
SI 32768
SF 150.9530986 MHz
WDW EM
SSB 0
LB 1.00 Hz
GB 0
PC 1.40

^{1}H NMR (400 MHz, $CDCl_3$) and LC–MS spectra of a second batch within the preparation of **13b**:

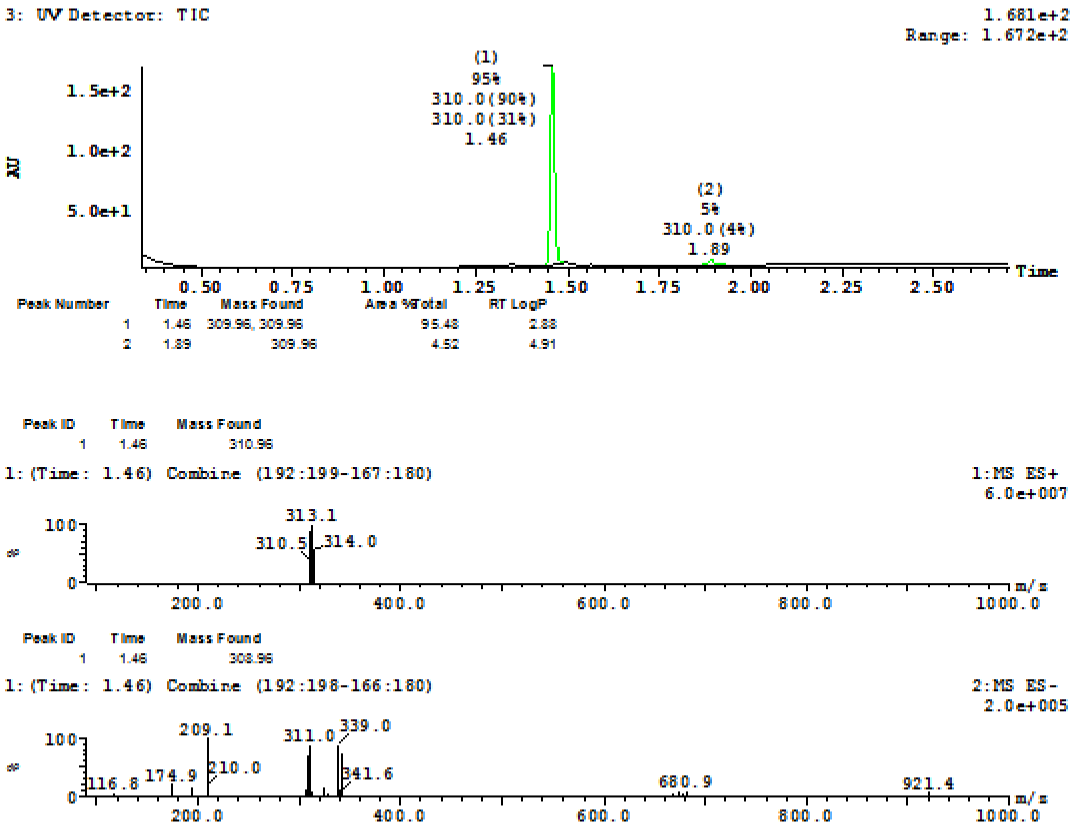

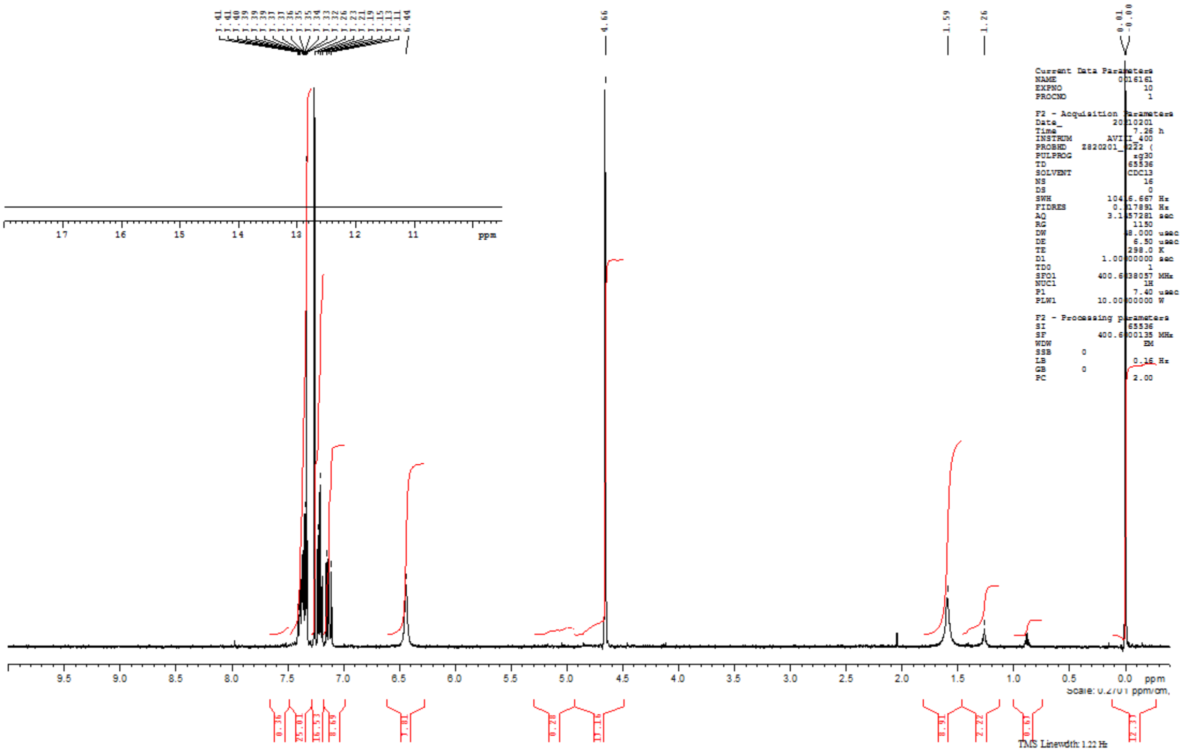

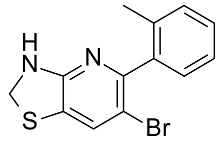

13c

Representative procedure for the synthesis of 6-bromo-5-(2-methylphenyl)-2,3-dihydro[1,3]thiazolo[4,5-*b*]pyridine (13c): To a stirred mixture of 6-bromopyridin-2-amine (**8**, 15.00 g, 86.69 mmol, 1.00 equiv), 2-methylphenylboronic acid (23.58 g, 173.39 mmol, 2.00 equiv) and K_2CO_3 (23.96 g, 173.39 mmol, 2.00 equiv) in a mixture of 1,4-dioxane (160 mL) and water (160 mL) at room temperature was added Pd(dppf)Cl_2 (3.17 g, 4.34 mmol, 0.05 equiv) and the mixture was stirred at 80 °C for 3 h. Thereafter, the reaction mixture was cooled to room temperature, diluted with water and extracted thoroughly with ethyl acetate. The combined organic layer was washed with brine, dried over Na_2SO_4, filtered and concentrated under reduced pressure. The remaining residue was purified via column chromatography (gradient ethyl acetate/hexane) to afford 6-(2-methylphenyl)pyridin-2-amine (**9c**, 9.40 g, 59%). ^{1}H NMR (400 MHz, $CDCl_3$): δ_H 7.51-7.49 (m, 1H), 7.37-7.34 (m, 1H), 7.30-7.23 (m, 2H), 6.74-6.72 (m, 1H), 6.48-6.44 (m, 1H), 4.52-4.46 (br. s, 2H, NH_2), 2.35 (s, 3H).

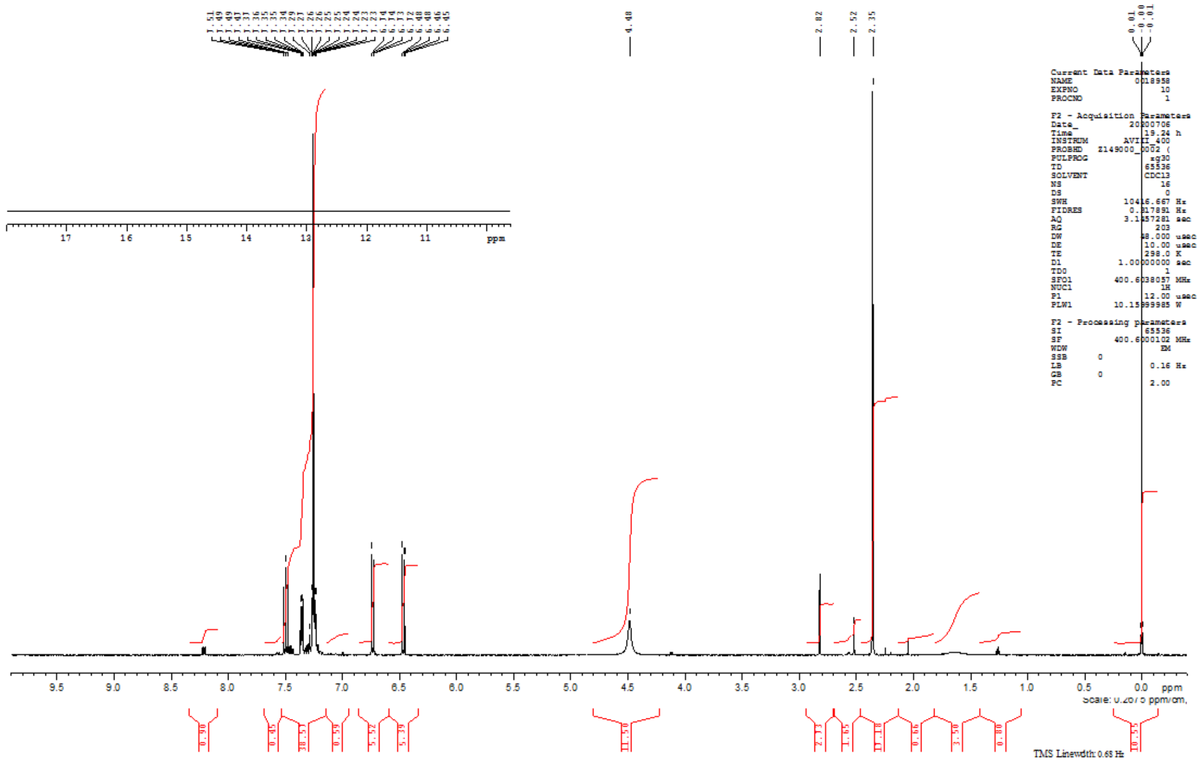

6-(2-Methylphenyl)pyridin-2-amine (**9c**, 8.97 g, 48.20 mmol, 1.0 equiv) was dissolved in acetonitrile (140 mL) and cooled to 0 °C. Thereafter, *N*-bromosuccinimide (18.87 g, 106.04 mmol, 2.2 equiv) was added carefully. The reaction mixture was warmed to room temperature and stirred for 2 h.

Subsequently, the reaction mixture was diluted with water and the resulting solid was filtered off. The solid was washed thoroughly with water and dried to afford compound 3,5-dibromo-6-(2-methylphenyl)pyridin-2-ylamine (**10c**, 13.10 g, 79%) as an orange solid. ^{1}H NMR (400 MHz, $CDCl_3$): δ_H 7.91 (s, 1H), 7.33-7.27 (m, 3H), 7.20-7.17 (m, 1H), 5.04-4.96 (br. s, 2H, NH_2), 2.18 (s, 3H). The reaction was repeated on the same scale to afford 26.17 g of **10c** in total

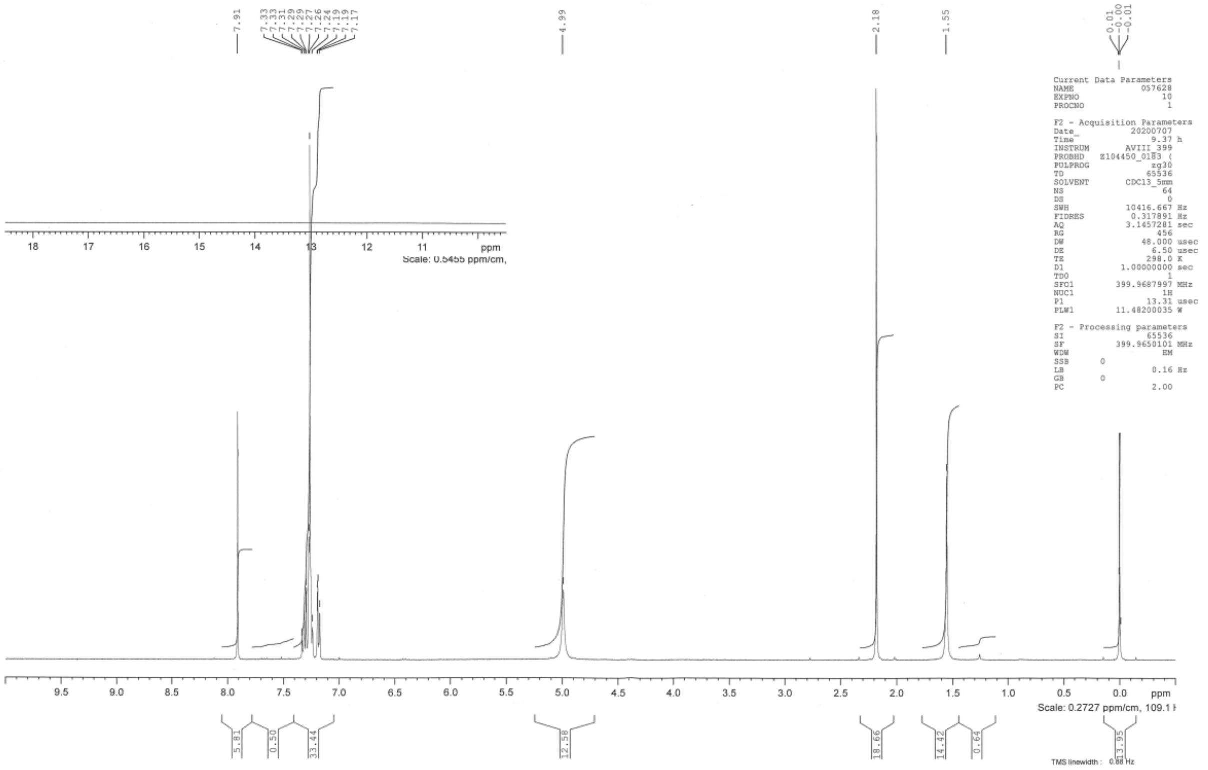

To a stirred solution of 3,5-dibromo-6-(2-methylphenyl)pyridin-2-amine (**10c**, 26.17 g, 76.50 mmol, 1.0 equiv) in *N*,*N*-dimethylformamide (120 mL) at room temperature was added potassium *O*-ethyl dithiocarbonate (27.81 g, 168.31 mmol, 2.2 equiv). The resulting mixture was heated under reflux conditions for 6.5 h. Thereafter, the reaction mixture was cooled to room temperature, poured onto ice water and acidified with 2 N HCl. The obtained precipitate was filtered, washed with water, collected and dried under reduced pressure to afford the 6-bromo-5-(2-methylphenyl)[1,3]thiazolo[4,5-*b*]pyridine-2-thiol and 6-bromo-5-(2-methylphenyl)[1,3]thiazolo[4,5-*b*]pyridine-2(3*H*)-thione thiol–thione tautomer **11c** (25.00 g, 97%). ^{1}H NMR (400 MHz, $CDCl_3$): δ_H 9.72 (br. s, 1H), 8.01 (s, 1H), 7.37-7.35 (m, 1H), 7.33-7.29 (m, 2H), 7.21-7.19 (m, 1H), 2.16 (s, 3H).

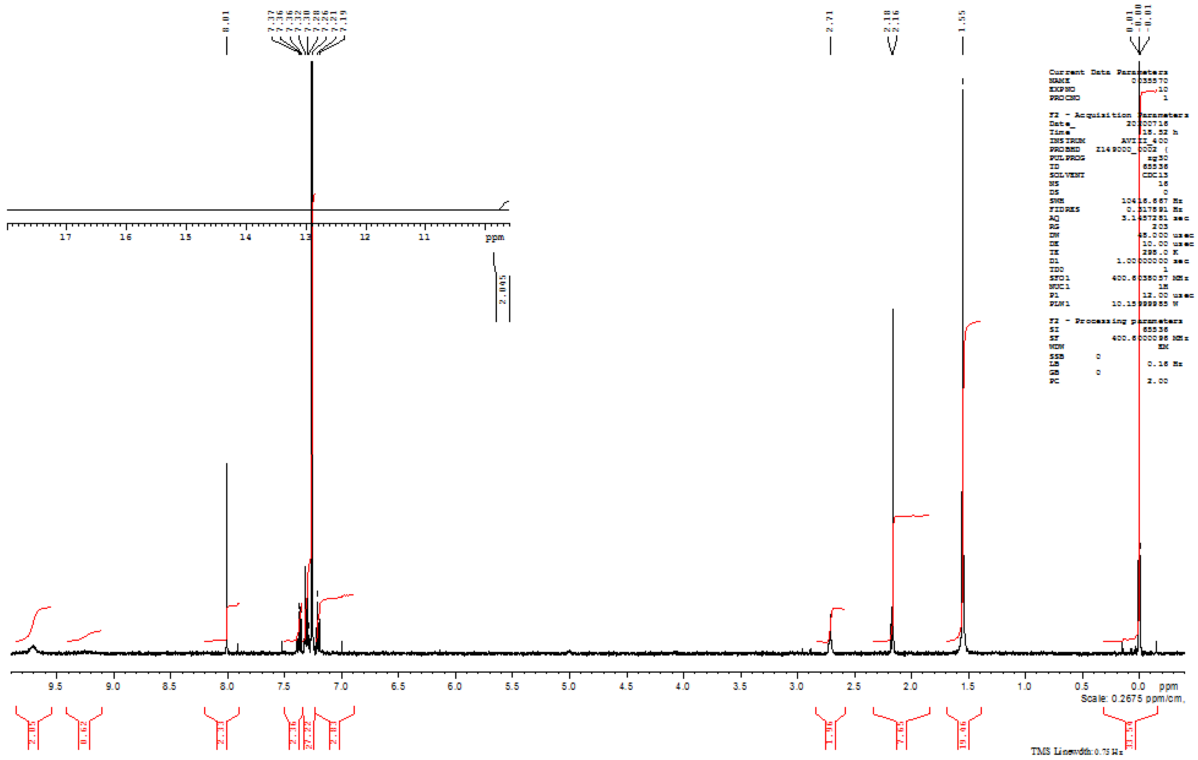

6-Bromo-5-(2-methylphenyl)[1,3]thiazolo[4,5-*b*]pyridine-2-thiol (**11c**, 26.81 g, 79 mmol, 1.0 equiv) was dissolved in acetic acid (350 mL), and iron powder (55.49 g, 994 mmol, 15 equiv) was added carefully in portions. The resulting reaction mixture was stirred at a temperature of 100 °C for 8 h. After full conversion (indicated by LC–MS), the reaction mixture was cooled to 60 °C, and the iron powder was filtered off. The remaining solution was diluted with water, and the resulting precipitate was filtered, washed with water and dried under reduced pressure. The remaining crude residue was redissolved in dichloromethane, then water was added, followed by thorough extraction. The combined organic layer was dried over magnesium sulfate, filtered and dried under reduced pressure. The remaining residue was purified via column chromatography (gradient ethyl acetate/hexane) to afford 6-bromo-5-(2-methylphenyl)[1,3]thiazolo[4,5-*b*]pyridine (**12c**, 8.75 g, 65%). ^{1}H NMR (400 MHz, $CDCl_3$): δ_H 9.32 (s, 1H), 8.63 (s, 1H), 7.38-7.35 (m, 1H), 7.32-7.27 (m, 3H), 2.18 (s, 3H).

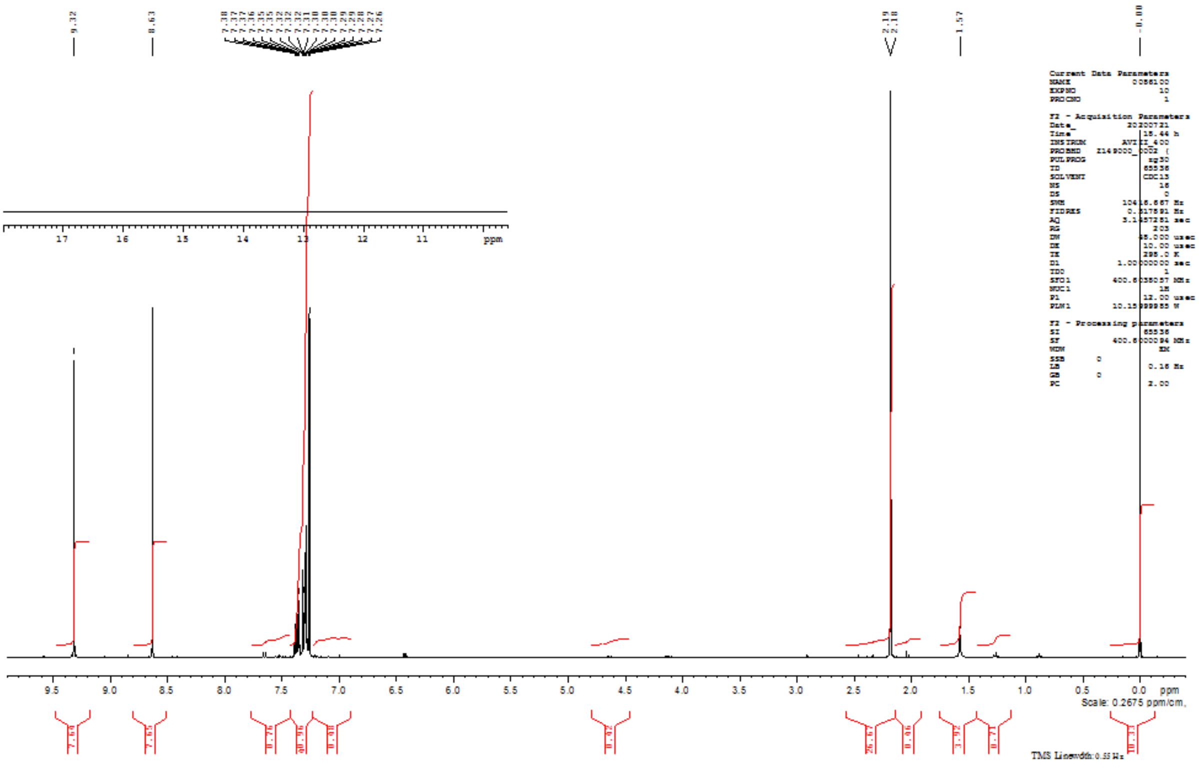

6-Bromo-5-(2-methylphenyl)[1,3]thiazolo[4,5-*b*]pyridine (**12c**, 1,000 mg, 3.07 mmol, 1.0 equiv) was dissolved in absolute toluene (10 mL) in a oven-dried round-bottom flask under argon, and ammonia borane (285 mg, 9.20 mmol, 3.0 equiv) and $B(C_6F_5)_3$ (79 mg, 0.15 mmol, 0.05 equiv) were added. The resulting reaction mixture was stirred at a temperature of 45 °C for 5 h and concentrated thereafter under reduced pressure. The remaining residue was redissolved in acetonitrile, formic acid was added, and the reaction mixture was stirred at room temperature for 2 h. The phases were separated via phase separator and the organic layer was concentrated under reduced pressure. The remaining crude product was purified via column chromatography (gradient ethyl acetate/hexane) to afford 6-bromo-5-(2-methylphenyl)-2,3-dihydro[1,3]thiazolo[4,5-*b*]pyridine (**13c**, yield after formic acid-mediated borane cleavage of intermediates **17d** and **17e**: 109 mg, 54%).^{1}H NMR (600 MHz, $CDCl_3$): δ 2.18 (s, 3H), 4.34 (s, 2H), 7.15–7.18 (m, 1H), 7.22–7.26 (m, 2H), 7.26–7.30 (m, 2H), 7.35–7.40 (m, 1H) ppm; ^{13}C NMR ($CDCl_3$, 151 MHz): δ 19.4 (CH_3), 48.9 (CH_2), 107.0 (C), 122.6 (C), 125.6 (CH), 128.4 (CH), 128.9 (CH), 130.0 (CH), 130.9 (CH), 136.1 (C), 139.7 (C), 151.2 (C), 160.0 (C) ppm; HRMS (ESI, m/z): calcd. for $C_{13}H_{12}BrN_2S$, 306.9919 $[M+H]^+$; found 306.9905.

^{1}H NMR (400 MHz, DMSO-d6$_6$):

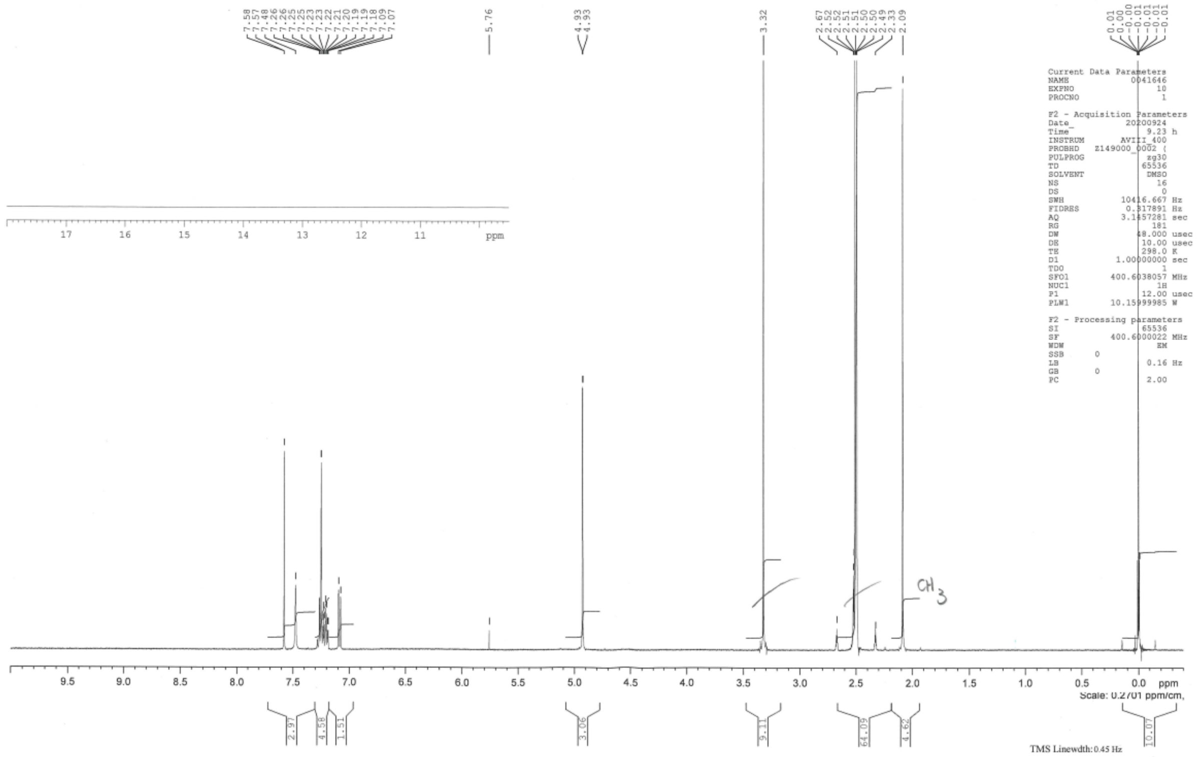

LC–MS:

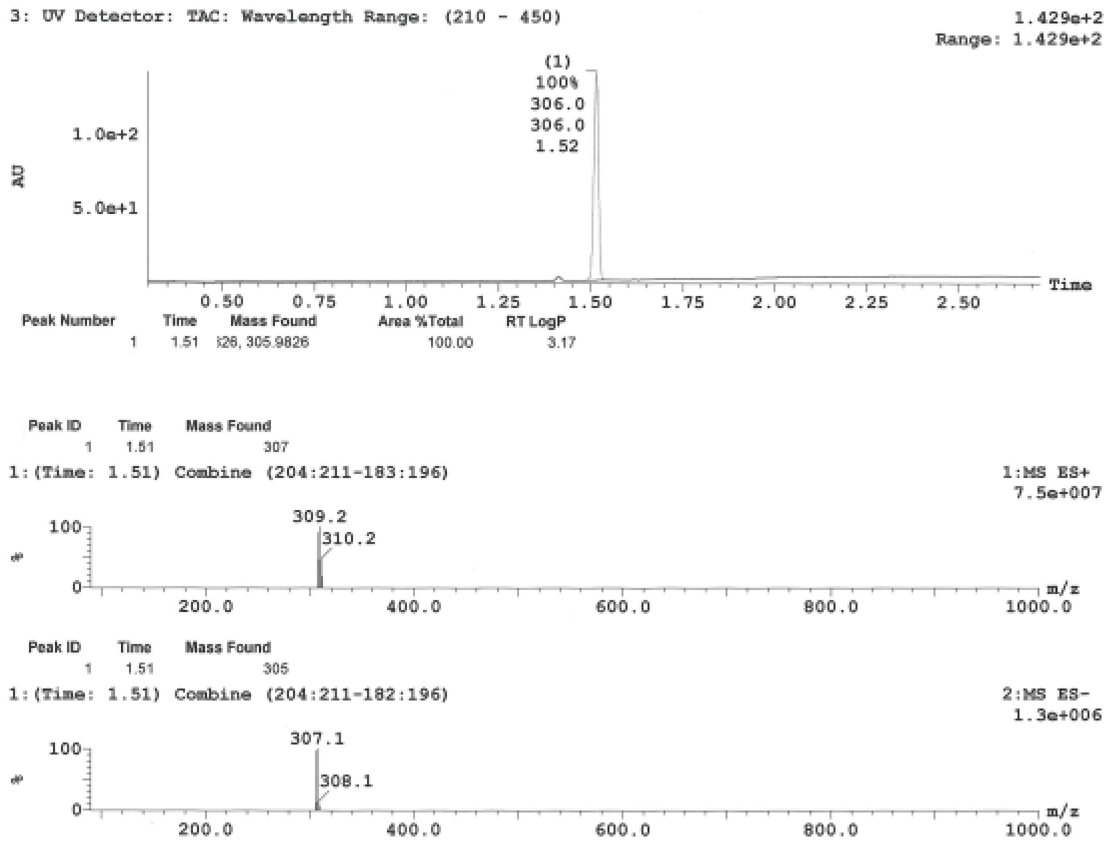

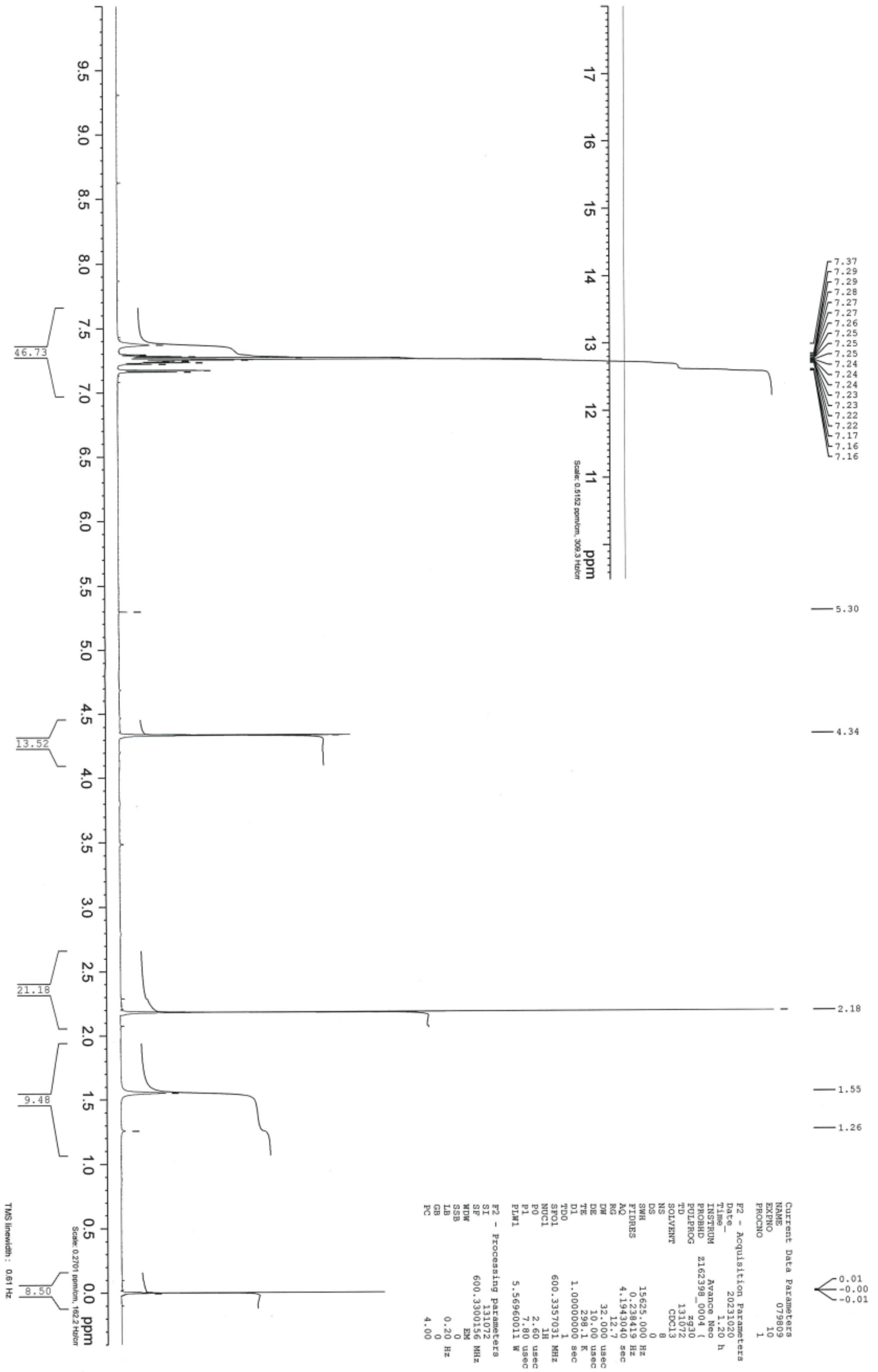
7.37
7.29
7.29
7.28
7.27
7.27
7.26
7.25
7.25
7.25
7.24
7.24
7.24
7.23
7.23
7.22
7.22
7.17
7.16
7.16
5.30
4.34
2.18
1.55
1.26
0.01
-0.00
-0.01
Current Data Parameters
NAME 079809
EXPNO 10
PROCNO 1
F2 - Acquisition Parameters
Date_ 20231020
Time 1.20 h
INSTRUM Avance Neo
PROBHD Z162398_0004 (
PULPROG zg30
TD 131072
SOLVENT CDCl3
NS 8
DS 0
SWH 15625.000 Hz
FIDRES 0.238419 Hz
AQ 4.1943040 sec
RG 12.7
DW 32.000 usec
DE 10.00 usec
TE 298.1 K
D1 1.00000000 sec
TD0 1
SFO1 600.3357031 MHz
NUC1 1H
P0 2.60 usec
P1 7.80 usec
PLW1 5.56960011 W
F2 - Processing parameters
SI 131072
SF 600.3300156 MHz
WDW EM
SSB 0
LB 0.20 Hz
GB 0
PC 4.00
17 16 15 14 13 12 11 ppm
Scale: 0.5152 ppm/cm, 309.3 Hz/cm
9.5 9.0 8.5 8.0 7.5 7.0 6.5 6.0 5.5 5.0 4.5 4.0 3.5 3.0 2.5 2.0 1.5 1.0 0.5 0.0 ppm
Scale: 0.2701 ppm/cm, 162.2 Hz/cm
46.73
13.52
21.18
9.48
8.50
TMS linewidth : 0.61 Hz

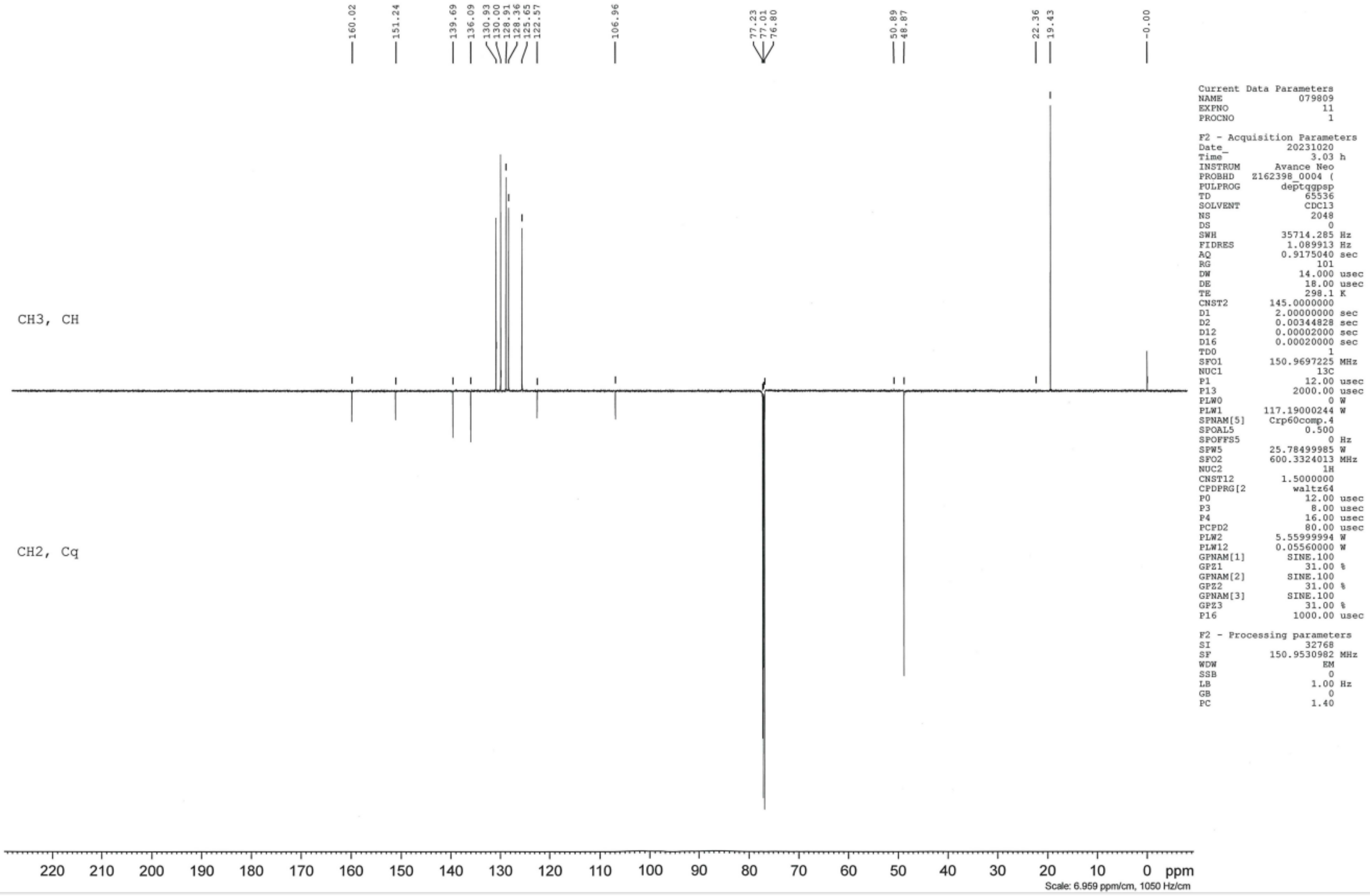
160.02
151.24
139.69
136.09
130.93
130.00
128.91
128.36
125.65
122.57
106.96
77.23
77.01
76.80
50.89
48.87
22.36
19.43
-0.00
CH3, CH
CH2, Cq
Current Data Parameters
NAME 079809
EXPNO 11
PROCNO 1
F2 - Acquisition Parameters
Date_ 20231020
Time 3.03 h
INSTRUM Avance Neo
PROBHD Z162398_0004 (
PULPROG deptqgpsp
TD 65536
SOLVENT CDCl3
NS 2048
DS 0
SWH 35714.285 Hz
FIDRES 1.089913 Hz
AQ 0.9175040 sec
RG 101
DW 14.000 usec
DE 18.00 usec
TE 298.1 K
CNST2 145.0000000
D1 2.00000000 sec
D2 0.00344828 sec
D12 0.00002000 sec
D16 0.00020000 sec
TD0 1
SFO1 150.9697225 MHz
NUC1 13C
P1 12.00 usec
P13 2000.00 usec
PLW0 0 W
PLW1 117.19000244 W
SPNAM[5] Crp60comp.4
SPOAL5 0.500
SPOFFS5 0 Hz
SPW5 25.78499985 W
SFO2 600.3324013 MHz
NUC2 1H
CNST12 1.5000000
CPDPRG[2 waltz64
P0 12.00 usec
P3 8.00 usec
P4 16.00 usec
PCPD2 80.00 usec
PLW2 5.55999994 W
PLW12 0.05560000 W
GPNAM[1] SINE.100
GPZ1 31.00 %
GPNAM[2] SINE.100
GPZ2 31.00 %
GPNAM[3] SINE.100
GPZ3 31.00 %
P16 1000.00 usec
F2 - Processing parameters
SI 32768
SF 150.9530982 MHz
WDW EM
SSB 0
LB 1.00 Hz
GB 0
PC 1.40
220 210 200 190 180 170 160 150 140 130 120 110 100 90 80 70 60 50 40 30 20 10 0 ppm
Scale: 6.959 ppm/cm, 1050 Hz/cm

1.2.3. General procedure for the synthesis of substituted 6-methyl-2,3-dihydro[1,3]thiazolo[4,5-*b*]pyridines 7a–c

The corresponding 6-bromo-5-phenyl[1,3]thiazolo[4,5-*b*]pyridine **13a–c** (4.56 mmol, 1.0 equiv), methylboronic acid (1.13 g, 18.24 mmol, 4.0 equiv), potassium phosphate (1.94 g, 9.12 mmol, 2.0 equiv), palladium(II) acetate (103 mg, 0.46 mmol, 0.1 equiv) and 2-dicyclohexylphosphino-2′,6′-dimethoxybiphenyl (579 mg, 1.37 mmol, 0.3 equiv) were consecutively dissolved in absolute toluene (40 mL) in a flame-dried round-bottom flask under argon. The resulting reaction mixture was stirred under reflux conditions for 3.5 h. After cooling to room temperature, water (80 mL) and toluene (20 mL) were added, followed by addition of ammonium pyrrolidinedithiocarbamate (328 mg, 2.00 mmol, 0.44 equiv). The reaction mixture was stirred for 1 h at room temperature, and the organic layer was washed with saturated aq $NaHCO_3$ solution, dried over anhydrous $MgSO_4$, filtered and concentrated under reduced pressure. The resulting crude residue was purified via column chromatography (gradient ethyl acetate/heptane) to afford the desired 5-phenyl-6-methyl[1,3]thiazolo[4,5-*b*]pyridine **5**, **15a** or **15c** as an intermediate. The corresponding 5-phenyl-6-methyl[1,3]thiazolo[4,5-*b*]pyridine **5**, **15a** or **15c** (1.23 mmol, 1.0 equiv) was dissolved in absolute toluene (10 mL) in a flame-dried round-bottom flask under argon. Ammonia borane (114 mg, 3.69 mmol, 3.0 equiv) and $B(C_6F_5)_3$ (32 mg, 0.06 mmol, 0.05 equiv) were added and the resulting reaction mixture was stirred at a temperature of 45 °C for 5 h and concentrated thereafter under reduced pressure. The remaining residue was redissolved in acetonitrile, formic acid was added, and the reaction mixture was stirred at room temperature for further 45 min. The phases were separated, and the organic layer was concentrated under reduced pressure. The resulting crude product was purified via column chromatography (gradient ethyl acetate/heptane) to afford the desired 5-phenyl-6-methyl-2,3-dihydro[1,3]thiazolo[4,5-*b*]pyridine **7a–c**.

1.2.4. Characterization of substituted 6-methyl-2,3-dihydro[1,3]thiazolo[4,5-b]pyridines 7a–c

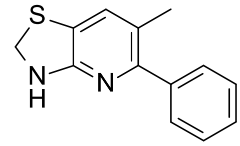

7a

6-Methyl-5-phenyl-2,3-dihydro[1,3]thiazolo[4,5-*b*]pyridine (**7a**, 610 mg, 55%). ^{1}H NMR (600 MHz, $CDCl_3$): δ 2.08 (s, 3H), 4.30 (s, 2H), 7.03 (s, 1H), 7.31–7.35 (m, 2H), 7.40–7.41 (m, 4H) ppm; ^{13}C NMR ($CDCl_3$, 151 MHz): δ 18.8 (CH_3), 48.6 (CH_2), 119.6 (C), 120.2 (C), 127.4 (CH), 128.1 (CH), 129.0 (CH), 130.5 (CH), 140.7 (C), 150.8 (C), 159.4 (C) ppm; HRMS (ESI, m/z): calcd. for $C_{13}H_{13}N_2S$, 229.0799 $[M+H]^+$; found 229.0790.

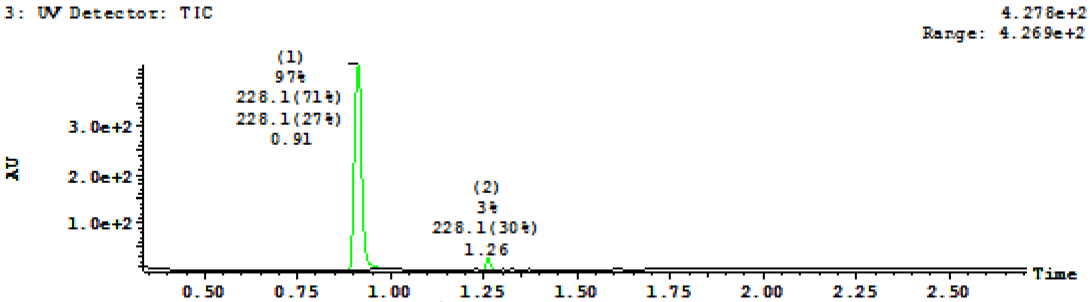

Peak Number	Time	Mass Found	Area %Total	RT LogP
1	0.91	228.07, 228.07	96.91	1.24
2	1.26	228.07	3.09	2.17

Peak ID	Time	Mass Found
1	0.91	229.07

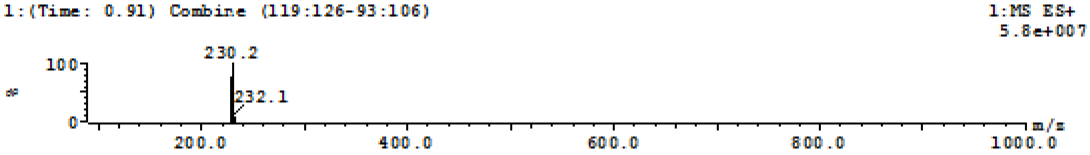

Peak ID	Time	Mass Found
1	0.91	273.07,227.07

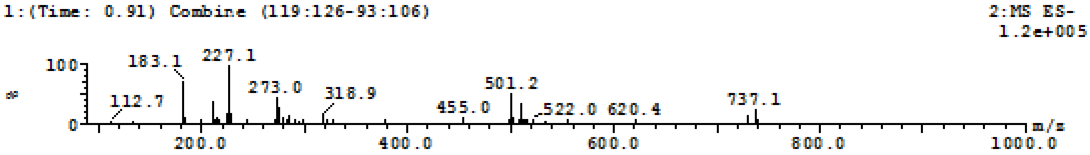

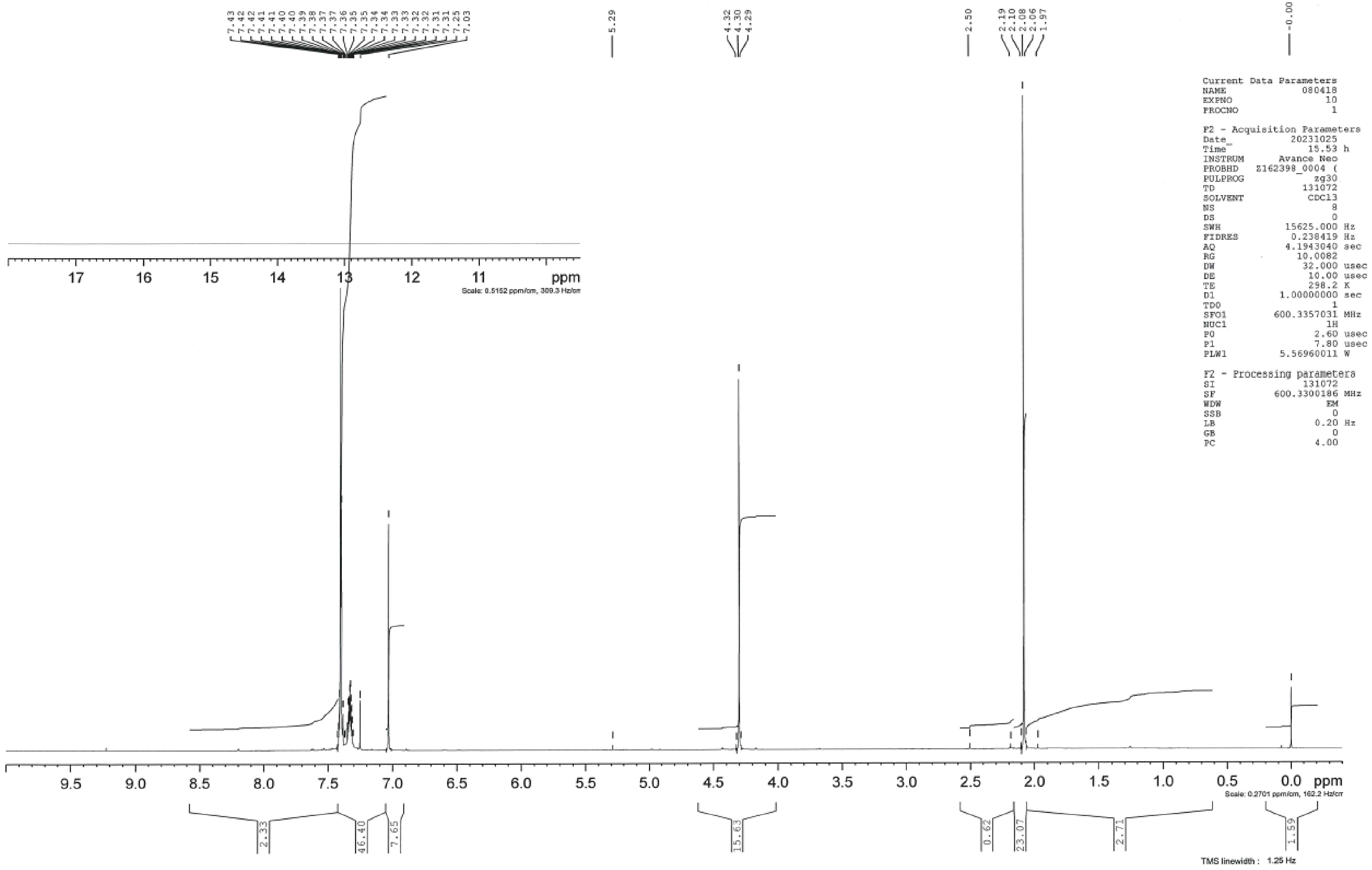
7.43 7.42 7.42 7.41 7.41 7.40 7.40 7.39 7.38 7.37 7.37 7.36 7.35 7.35 7.34 7.34 7.33 7.33 7.32 7.32 7.31 7.31 7.25 7.03
5.29
4.32 4.30 4.29
2.50
2.19 2.10 2.08 2.06 1.97
-0.00
Current Data Parameters
NAME 080418
EXPNO 10
PROCNO 1
F2 - Acquisition Parameters
Date_ 20231025
Time_ 15.53 h
INSTRUM Avance Neo
PROBHD Z162398_0004 (
PULPROG zg30
TD 131072
SOLVENT CDCl3
NS 8
DS 0
SWH 15625.000 Hz
FIDRES 0.238419 Hz
AQ 4.1943040 sec
RG 10.0082
DW 32.000 usec
DE 10.00 usec
TE 298.2 K
D1 1.00000000 sec
TD0 1
SFO1 600.3357031 MHz
NUC1 1H
P0 2.60 usec
P1 7.80 usec
PLW1 5.56960011 W
F2 - Processing parameters
SI 131072
SF 600.3300186 MHz
WDW EM
SSB 0
LB 0.20 Hz
GB 0
PC 4.00
17 16 15 14 13 12 11 ppm
Scale: 0.5152 ppm/cm, 309.3 Hz/cm
9.5 9.0 8.5 8.0 7.5 7.0 6.5 6.0 5.5 5.0 4.5 4.0 3.5 3.0 2.5 2.0 1.5 1.0 0.5 0.0 ppm
Scale: 0.2701 ppm/cm, 162.2 Hz/cm
2.33
46.40
7.65
15.63
0.62
23.07
2.71
1.59
TMS linewidth : 1.25 Hz

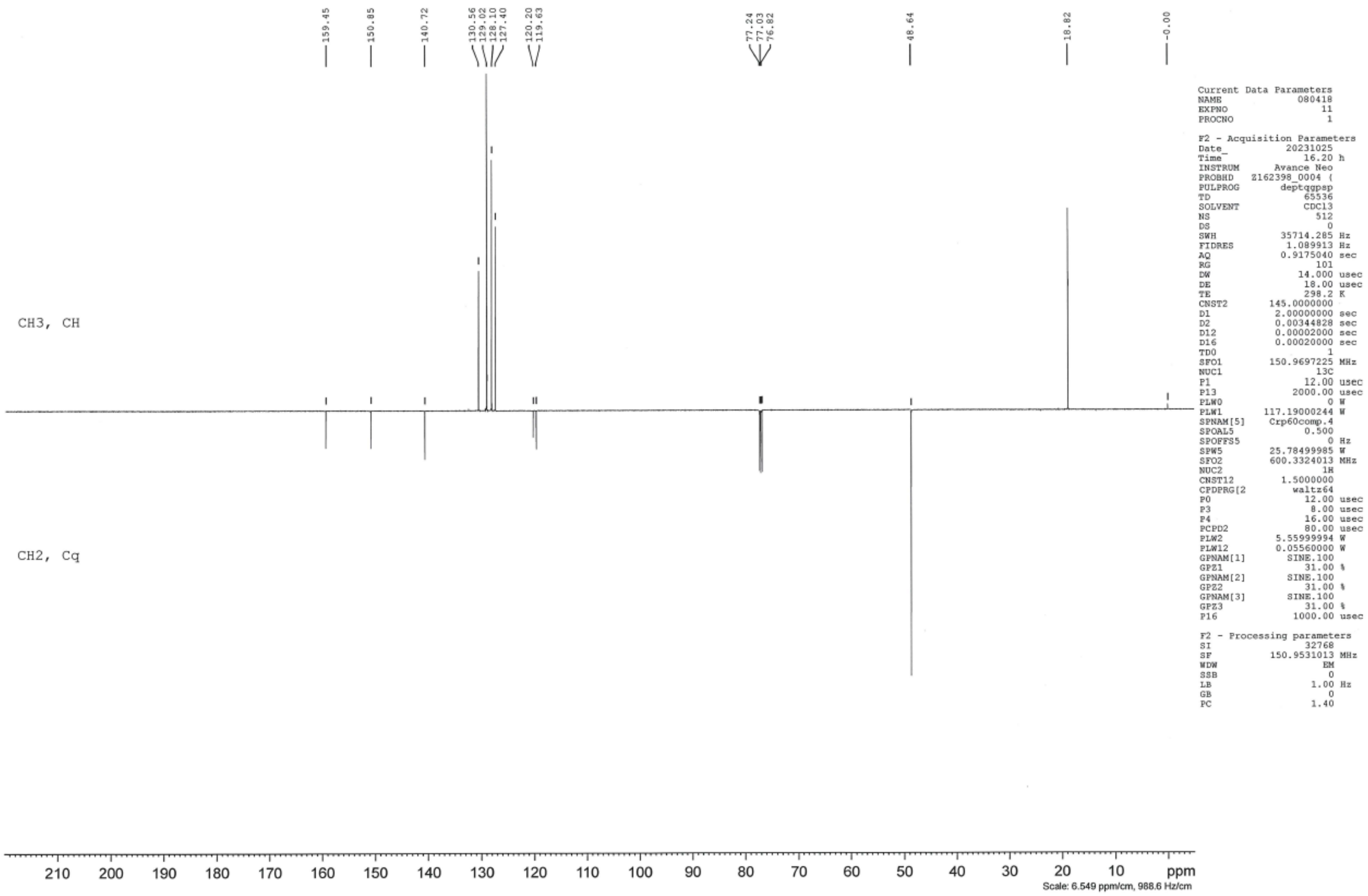
159.45
150.85
140.72
130.56
129.02
128.10
127.40
120.20
119.63
77.24
77.03
76.82
48.64
18.82
-0.00
CH3, CH
CH2, Cq
Current Data Parameters
NAME 080418
EXPNO 11
PROCNO 1
F2 - Acquisition Parameters
Date_ 20231025
Time 16.20 h
INSTRUM Avance Neo
PROBHD Z162398_0004 (
PULPROG deptqgpsp
TD 65536
SOLVENT CDCl3
NS 512
DS 0
SWH 35714.285 Hz
FIDRES 1.089913 Hz
AQ 0.9175040 sec
RG 101
DW 14.000 usec
DE 18.00 usec
TE 298.2 K
CNST2 145.0000000
D1 2.00000000 sec
D2 0.00344828 sec
D12 0.00002000 sec
D16 0.00020000 sec
TD0 1
SFO1 150.9697225 MHz
NUC1 13C
P1 12.00 usec
P13 2000.00 usec
PLW0 0 W
PLW1 117.19000244 W
SPNAM[5] Crp60comp.4
SPOAL5 0.500
SPOFFS5 0 Hz
SPW5 25.78499985 W
SFO2 600.3324013 MHz
NUC2 1H
CNST12 1.5000000
CPDPRG[2 waltz64
P0 12.00 usec
P3 8.00 usec
P4 16.00 usec
PCPD2 80.00 usec
PLW2 5.55999994 W
PLW12 0.05560000 W
GPNAM[1] SINE.100
GPZ1 31.00 %
GPNAM[2] SINE.100
GPZ2 31.00 %
GPNAM[3] SINE.100
GPZ3 31.00 %
P16 1000.00 usec
F2 - Processing parameters
SI 32768
SF 150.9531013 MHz
WDW EM
SSB 0
LB 1.00 Hz
GB 0
PC 1.40
210 200 190 180 170 160 150 140 130 120 110 100 90 80 70 60 50 40 30 20 10 ppm
Scale: 6.549 ppm/cm, 988.6 Hz/cm

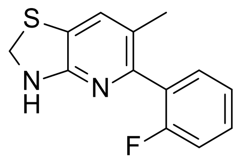

7b

5-(2-Fluorophenyl)-6-methyl-2,3-dihydro[1,3]thiazolo[4,5-*b*]pyridine (**7b**, 630 mg, 62%). ^{1}H NMR (600 MHz, $CDCl_3$): δ 2.00 (s, 3H), 4.55 (s, 2H), 6.61 (brs, 1H), 7.07 (s, 1H), 7.09–7.12 (m, 1H), 7.19–7.21 (m, 1H), 7.32–7.36 (m, 2H) ppm; ^{13}C NMR ($CDCl_3$, 151 MHz): δ 17.96 (CH_3), 17.99 (CH_3), 48.8 (CH_2), 115.6 (CH), 115.7 (CH), 121.0 (C), 121.8 (C), 124.15 (CH), 124.18 (CH), 129.59 (CH), 129.64 (CH), 130.2 (CH), 131.40 (CH), 131.43 (CH), 145.6 (C), 158.9 (C), 159.2 (C), 160.5 (C) ppm; HRMS (ESI, m/z): calcd. for $C_{13}H_{12}FN_2S$, 247.0705 $[M+H]^+$; found 247.0724.

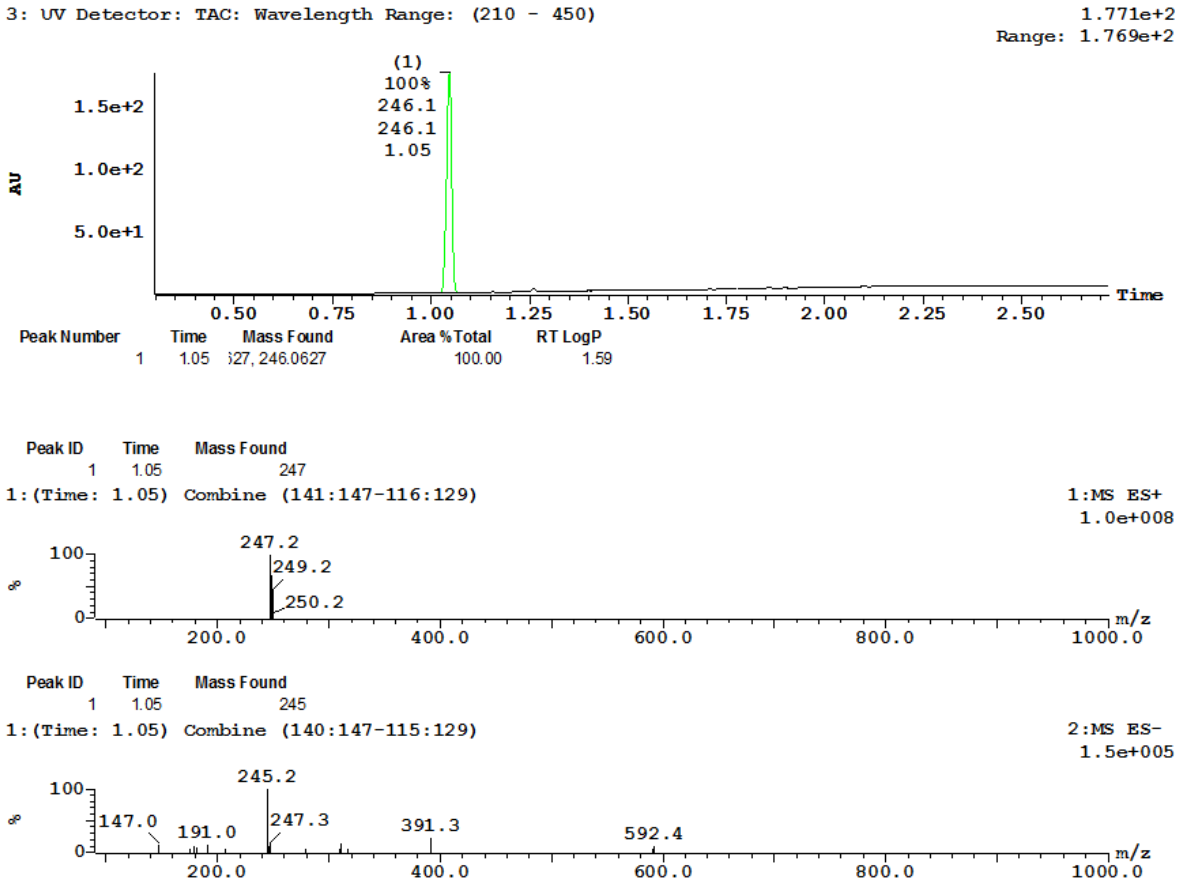

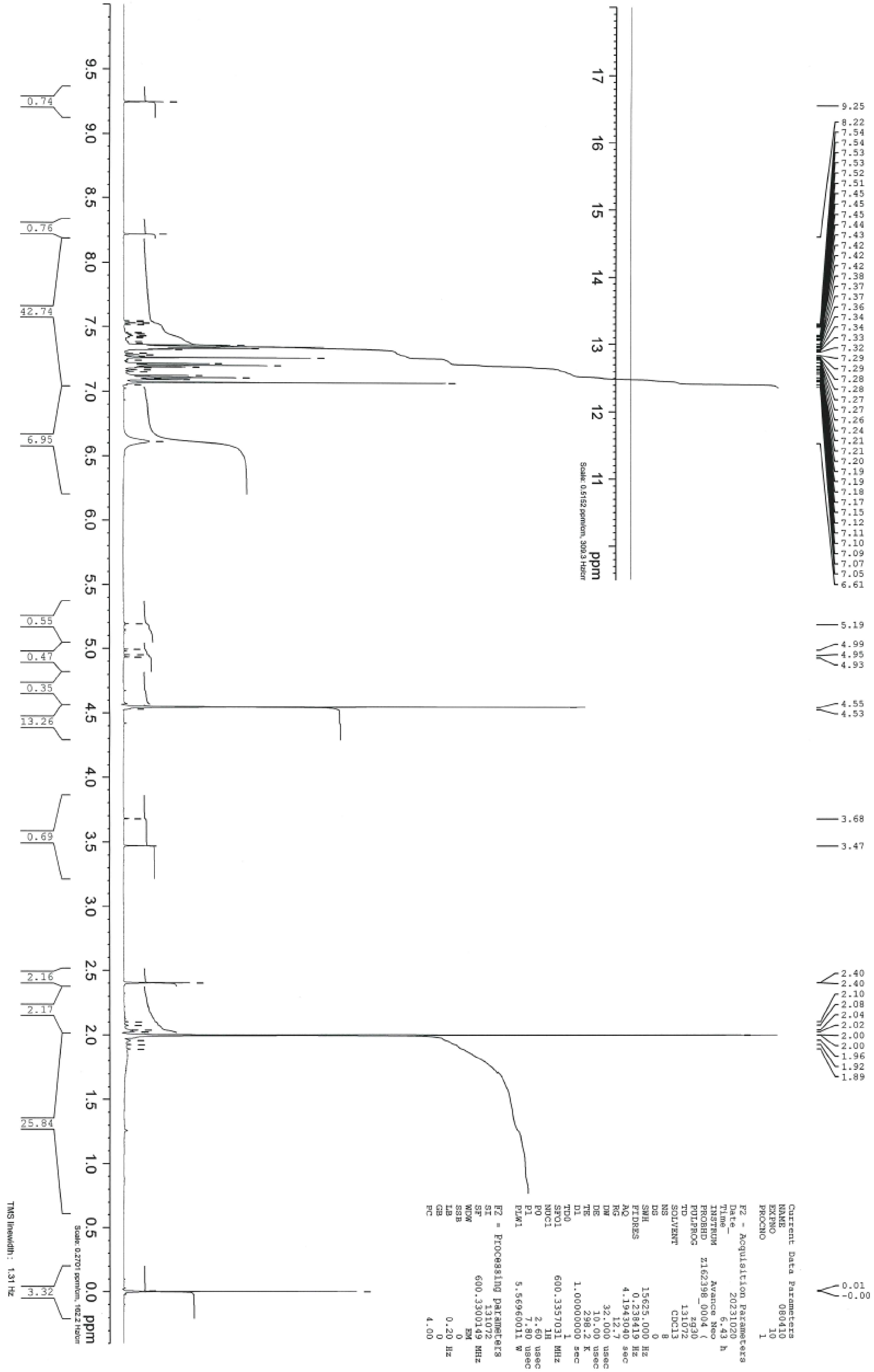
Current Data Parameters
NAME 080410
EXPNO 10
PROCNO 1
F2 - Acquisition Parameters
Date_ 20231020
Time 6.43 h
INSTRUM Avance Neo
PROBHD Z162398_0004 (
PULPROG zg30
TD 131072
SOLVENT CDCl3
NS 8
DS 0
SWH 15625.000 Hz
FIDRES 0.238419 Hz
AQ 4.1943040 sec
RG 12.7
DW 32.000 usec
DE 10.00 usec
TE 298.2 K
D1 1.00000000 sec
TD0 1
SFO1 600.3357031 MHz
NUC1 1H
P0 2.60 usec
P1 7.80 usec
PLW1 5.56960011 W
F2 - Processing parameters
SI 131072
SF 600.3300149 MHz
WDW EM
SSB 0
LB 0.20 Hz
GB 0
PC 4.00

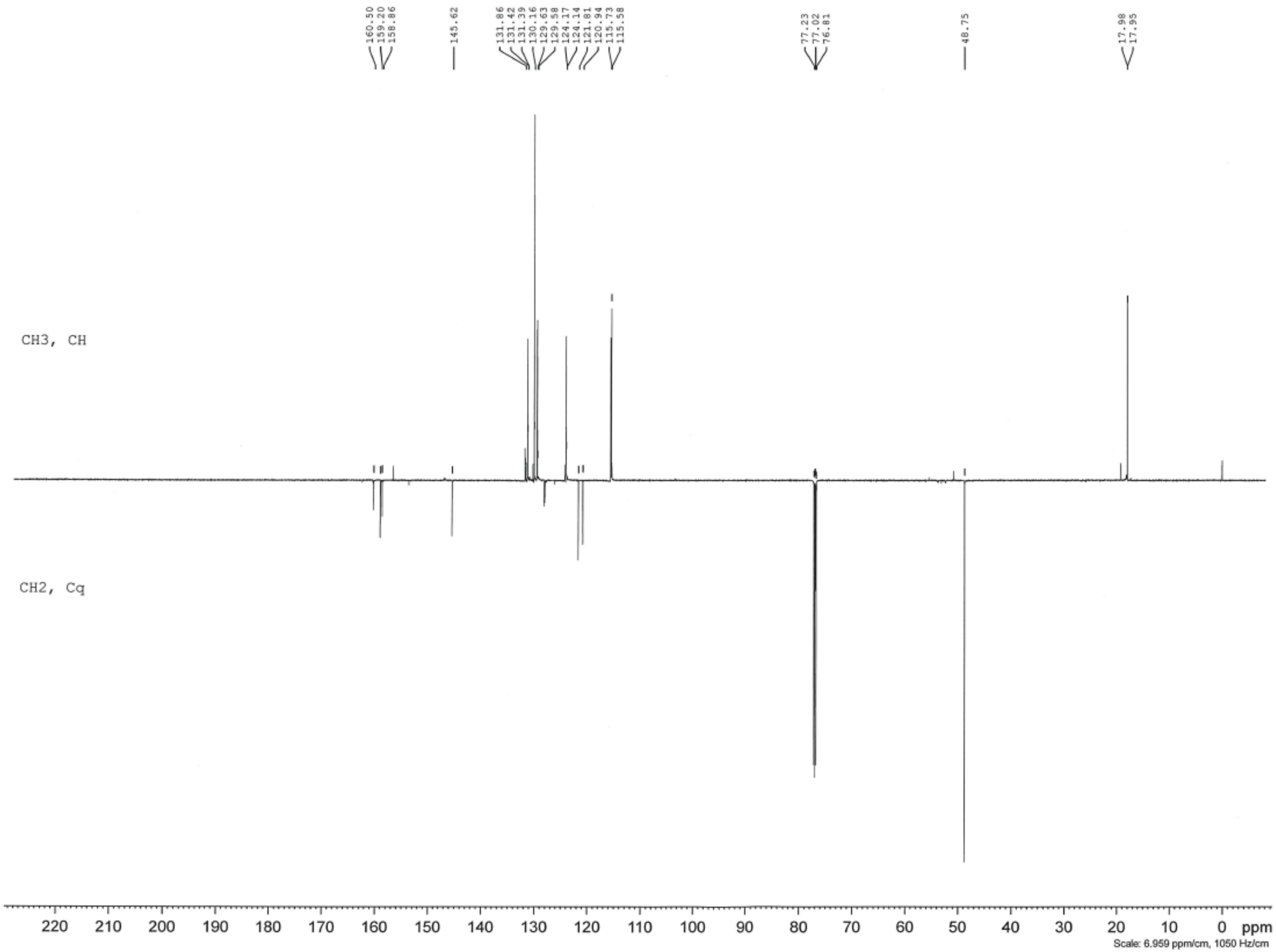


```
Current Data Parameters
NAME              080410
EXPNO                 11
PROCNO                 1

F2 - Acquisition Parameters
Date_           20231020
Time                8.25 h
INSTRUM       Avance Neo
PROBHD   Z162398_0004 (
PULPROG        deptqgpsp
TD                 65536
SOLVENT            CDCl3
NS                  2048
DS                     0
SWH            35714.285 Hz
FIDRES          1.089913 Hz
AQ             0.9175040 sec
RG                   101
DW                14.000 usec
DE                 18.00 usec
TE                 298.1 K
CNST2        145.0000000
D1            2.00000000 sec
D2            0.00344828 sec
D12           0.00002000 sec
D16           0.00020000 sec
TD0                    1
SFO1         150.9697225 MHz
NUC1                 13C
P1                 12.00 usec
P13              2000.00 usec
PLW0                   0 W
PLW1        117.19000244 W
SPNAM[5]    Crp60comp.4
SPOAL5             0.500
SPOFFS5                0 Hz
SPW5         25.78499985 W
SFO2         600.3324013 MHz
NUC2                  1H
CNST12         1.5000000
CPDPRG[2          waltz64
P0                 12.00 usec
P3                  8.00 usec
P4                 16.00 usec
PCPD2              80.00 usec
PLW2          5.55999994 W
PLW12         0.05560000 W
GPNAM[1]        SINE.100
GPZ1               31.00 %
GPNAM[2]        SINE.100
GPZ2               31.00 %
GPNAM[3]        SINE.100
GPZ3               31.00 %
P16              1000.00 usec

F2 - Processing parameters
SI                 32768
SF           150.9530989 MHz
WDW                   EM
SSB                    0
LB                  1.00 Hz
GB                     0
PC                  1.40
```

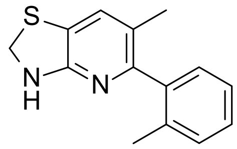

7c

6-Methyl-5-(2-methylphenyl)-2,3-dihydro[1,3]thiazolo[4,5-*b*]pyridine (**7c**, 212 mg, 52%). ^{1}H NMR (600 MHz, $CDCl_3$): δ 1.85 (s, 3H), 2.12 (s, 3H), 4.22 (s, 2H), 7.02 (s, 1H), 7.13 (d, *J* = 7.2 Hz, 1H), 7.19–7.26 (m, 3H), 7.69 (brs, 1H) ppm; ^{13}C NMR ($CDCl_3$, 151 MHz): δ 17.8 (CH_3), 19.4 (CH_3), 48.5 (CH_2), 119.7 (C), 120.0 (C), 125.6 (CH), 127.6 (CH), 129.0 (CH), 129.9 (CH), 130.0 (CH), 136.1 (C), 140.2 (C), 151.0 (C), 159.3 (C) ppm; HRMS (ESI, m/z): calcd. for $C_{14}H_{15}N_2S$, 243.0956 $[M+H]^+$; found 243.0963.

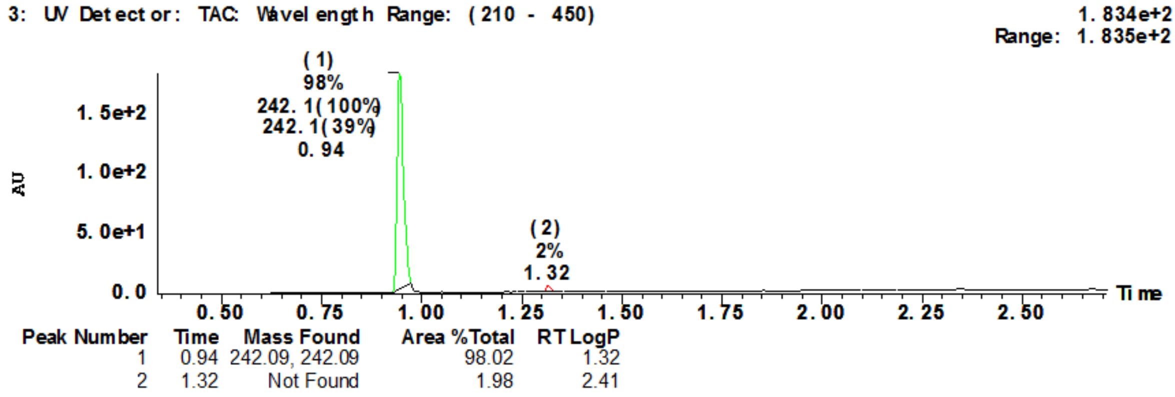

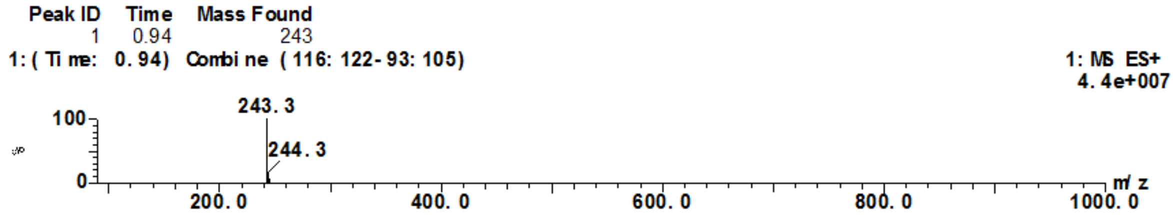

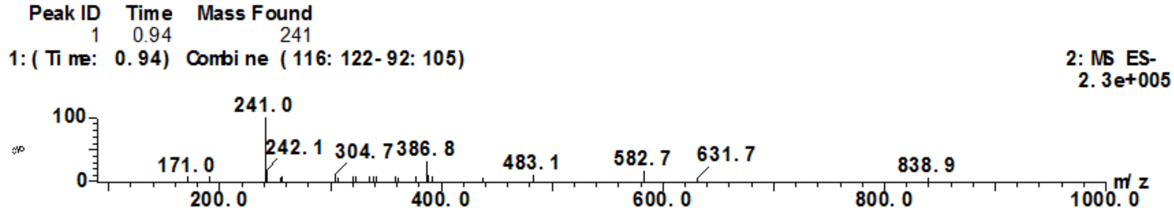

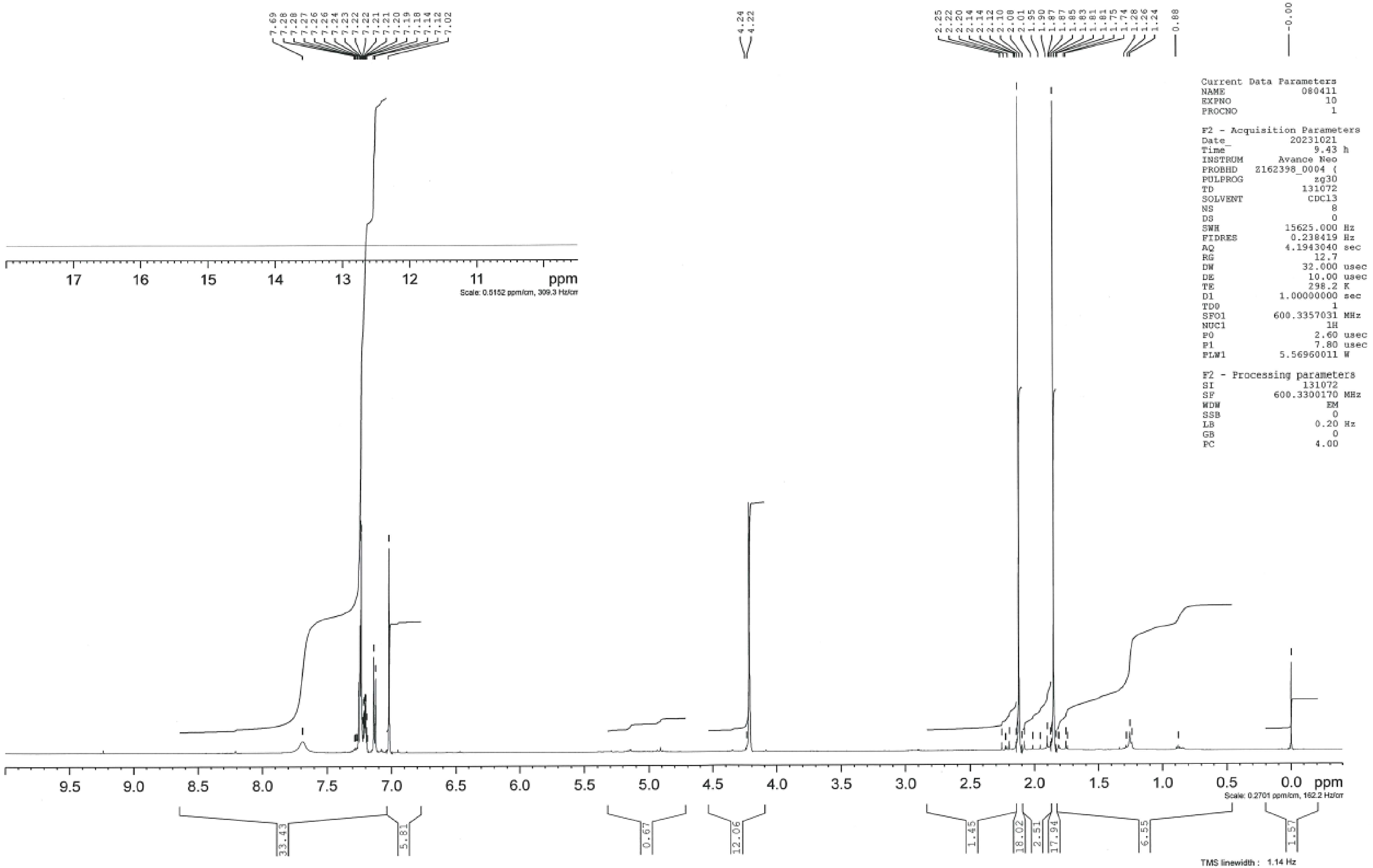
Current Data Parameters
NAME 080411
EXPNO 10
PROCNO 1
F2 - Acquisition Parameters
Date_ 20231021
Time 9.43 h
INSTRUM Avance Neo
PROBHD Z162398_0004 (
PULPROG zg30
TD 131072
SOLVENT CDCl3
NS 8
DS 0
SWH 15625.000 Hz
FIDRES 0.238419 Hz
AQ 4.1943040 sec
RG 12.7
DW 32.000 usec
DE 10.00 usec
TE 298.2 K
D1 1.00000000 sec
TD0 1
SFO1 600.3357031 MHz
NUC1 1H
P0 2.60 usec
P1 7.80 usec
PLW1 5.56960011 W
F2 - Processing parameters
SI 131072
SF 600.3300170 MHz
WDW EM
SSB 0
LB 0.20 Hz
GB 0
PC 4.00
7.69 7.28 7.28 7.27 7.26 7.26 7.24 7.23 7.22 7.22 7.21 7.21 7.20 7.19 7.18 7.14 7.12 7.02
4.24 4.22
2.25 2.22 2.20 2.14 2.14 2.12 2.10 2.08 2.01 1.95 1.90 1.87 1.87 1.85 1.83 1.81 1.81 1.75 1.74 1.28 1.26 1.24
0.88
-0.00
33.43
5.81
0.67
12.06
1.45
18.02
2.51
17.94
6.55
1.57
ppm
Scale: 0.5152 ppm/cm, 309.3 Hz/cm
Scale: 0.2701 ppm/cm, 162.2 Hz/cm
TMS linewidth : 1.14 Hz

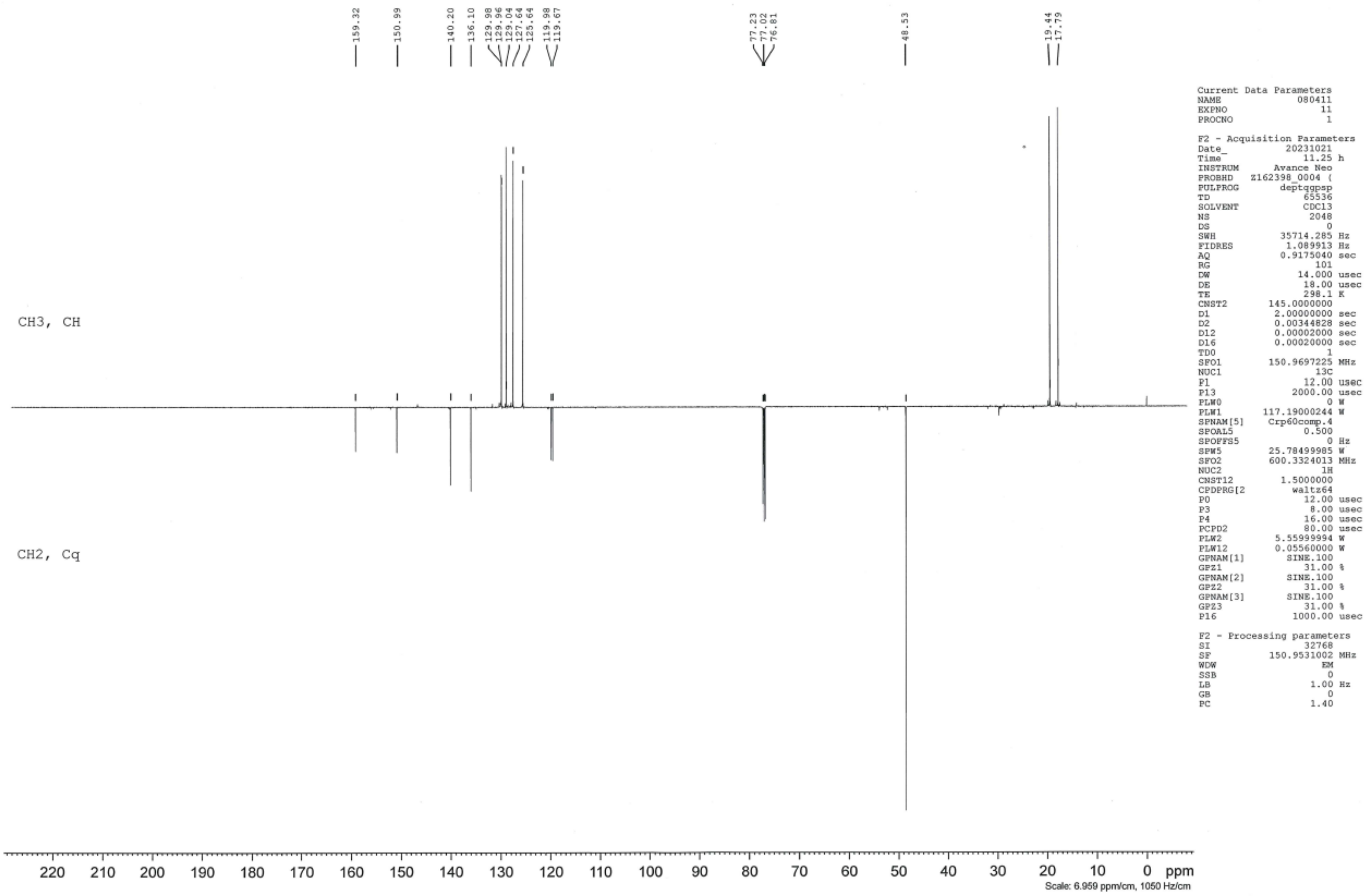

159.32
150.99
140.20
136.10
129.98
129.96
129.04
127.64
125.64
119.98
119.67
77.23
77.02
76.81
48.53
19.44
17.79
CH3, CH
CH2, Cq
220 210 200 190 180 170 160 150 140 130 120 110 100 90 80 70 60 50 40 30 20 10 0 ppm
Scale: 6.959 ppm/cm, 1050 Hz/cm
Current Data Parameters
NAME 080411
EXPNO 11
PROCNO 1
F2 - Acquisition Parameters
Date_ 20231021
Time 11.25 h
INSTRUM Avance Neo
PROBHD Z162398_0004 (
PULPROG deptqgpsp
TD 65536
SOLVENT CDCl3
NS 2048
DS 0
SWH 35714.285 Hz
FIDRES 1.089913 Hz
AQ 0.9175040 sec
RG 101
DW 14.000 usec
DE 18.00 usec
TE 298.1 K
CNST2 145.0000000
D1 2.00000000 sec
D2 0.00344828 sec
D12 0.00002000 sec
D16 0.00020000 sec
TD0 1
SFO1 150.9697225 MHz
NUC1 13C
P1 12.00 usec
P13 2000.00 usec
PLW0 0 W
PLW1 117.19000244 W
SPNAM[5] Crp60comp.4
SPOAL5 0.500
SPOFFS5 0 Hz
SPW5 25.78499985 W
SFO2 600.3324013 MHz
NUC2 1H
CNST12 1.5000000
CPDPRG[2 waltz64
P0 12.00 usec
P3 8.00 usec
P4 16.00 usec
PCPD2 80.00 usec
PLW2 5.55999994 W
PLW12 0.05560000 W
GPNAM[1] SINE.100
GPZ1 31.00 %
GPNAM[2] SINE.100
GPZ2 31.00 %
GPNAM[3] SINE.100
GPZ3 31.00 %
P16 1000.00 usec
F2 - Processing parameters
SI 32768
SF 150.9531002 MHz
WDW EM
SSB 0
LB 1.00 Hz
GB 0
PC 1.40

1.2.5. General procedure for the synthesis of N-acylated 6-bromo-2,3-dihydro[1,3]thiazolo[4,5-*b*]pyridines 14a–c

The corresponding acid chloride (0.36 mmol, 1.1 equiv) and triethylamine (0.10 mL, 0.72 mmol, 2.2 equiv) were added to a stirred solution of the corresponding 6-bromo-5-phenyl-2,3-dihydro[1,3]thiazolo[4,5-*b*]pyridine **13** (0.33 mmol, 1.00 equiv) in absolute dichloromethane (5 mL). The resulting reaction mixture was stirred at room temperature for 2 h, followed by dilution with dichloromethane and water, and subsequent extraction and phase separation. The aqueous layer was thoroughly extracted with dichloromethane, and the combined organic layers were dried over anhydrous $MgSO_4$, filtered, and concentrated under reduced pressure. The resulting crude product was purified via column chromatography (gradient ethyl acetate/heptane) to afford the desired target compounds **14a**–**c**.

1.2.6. Characterization of N-acylated 6-bromo-2,3-dihydro[1,3]thiazolo[4,5-*b*]pyridines 14a–c

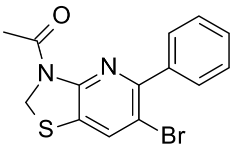

14a

1-(6-Bromo-5-phenyl[1,3]thiazolo[4,5-*b*]pyridin-3(2*H*)-yl)ethanone (**14a**, 92 mg, 72%). ^{1}H NMR (600 MHz, $CDCl_3$): δ 2.65 (s, 3H), 5.35 (s, 2H), 7.39–7.47 (m, 3H), 7.65–7.66 (m, 1H), 7.66–7.70 (m, 2H) ppm; ^{13}C NMR ($CDCl_3$, 151 MHz): δ 25.0 (CH_3), 49.9 (CH_2), 112.8 (C), 126.3 (C), 127.9 (CH), 128.7 (CH), 129.7 (CH), 134.6 (CH), 138.8 (C), 150.7 (C), 151.2 (C), 170.4 (C) ppm; HRMS (ESI, m/z): calcd. for $C_{14}H_{12}BrN_2OS$, 334.9854 $[M+H]^+$; found 334.9854.

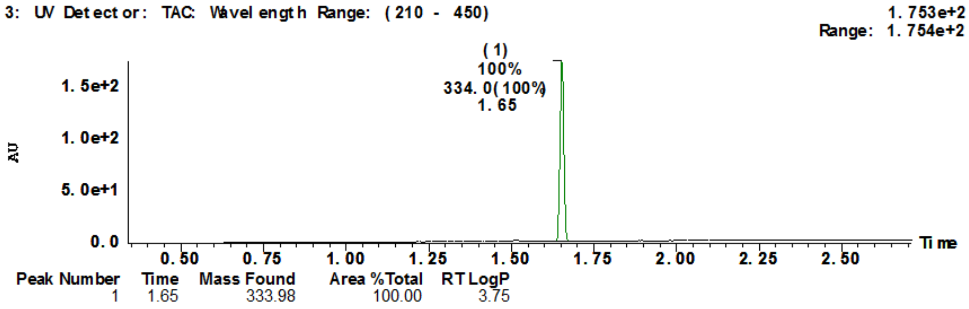

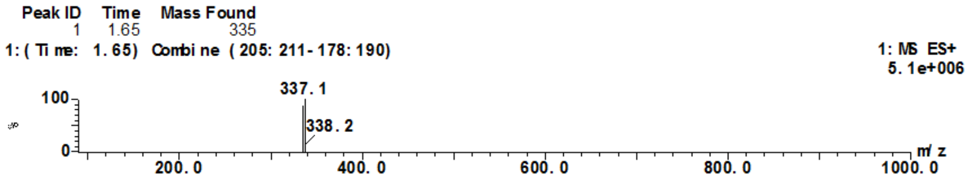

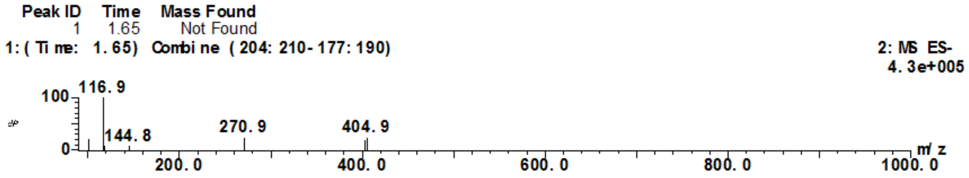

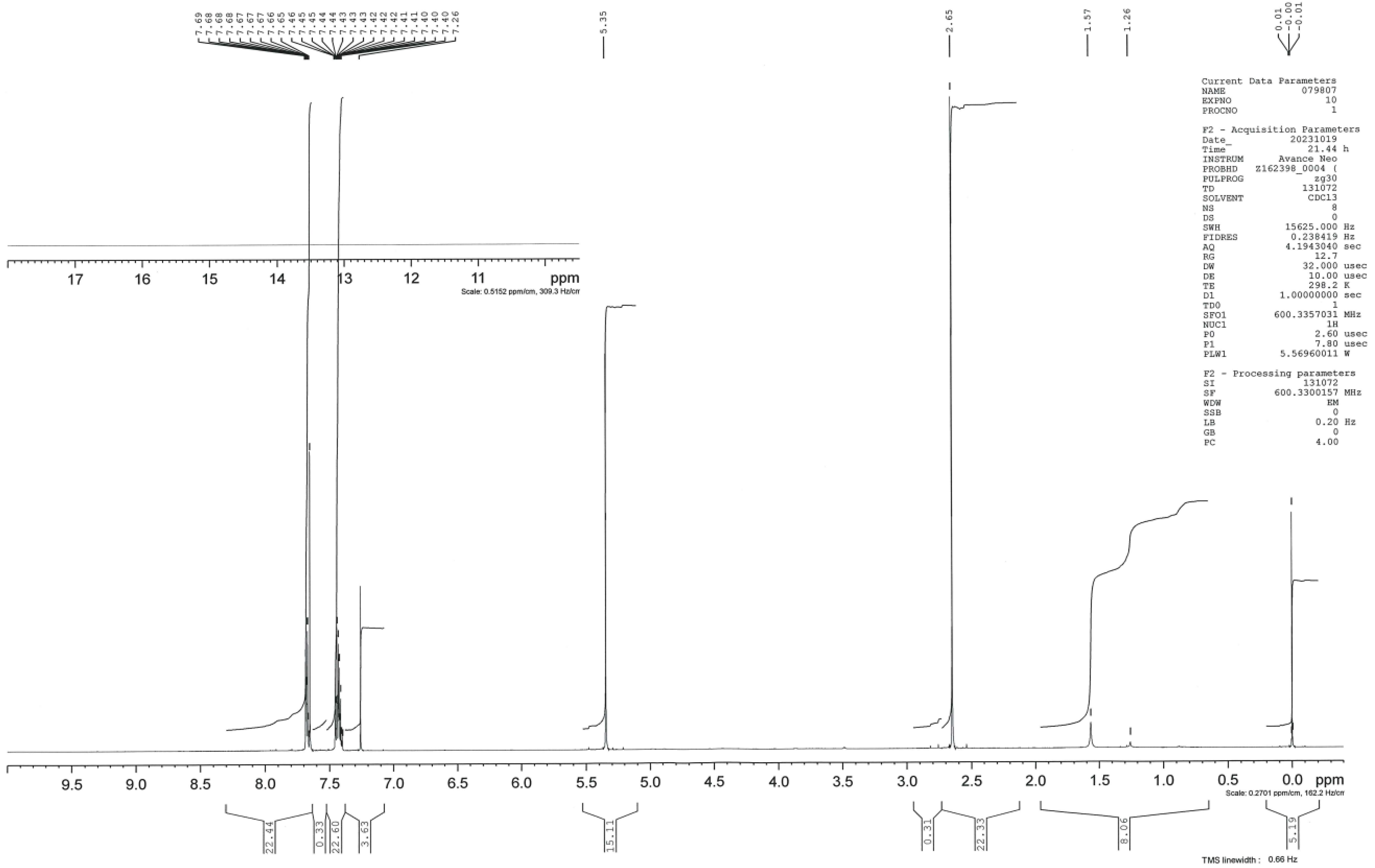
7.69 7.68 7.68 7.68 7.67 7.67 7.67 7.66 7.65 7.46 7.45 7.45 7.44 7.44 7.43 7.43 7.43 7.42 7.42 7.42 7.41 7.41 7.40 7.40 7.40 7.26
5.35
2.65
1.57
1.26
0.01 -0.00 -0.01
Current Data Parameters
NAME 079807
EXPNO 10
PROCNO 1
F2 - Acquisition Parameters
Date_ 20231019
Time_ 21.44 h
INSTRUM Avance Neo
PROBHD Z162398_0004 (
PULPROG zg30
TD 131072
SOLVENT CDCl3
NS 8
DS 0
SWH 15625.000 Hz
FIDRES 0.238419 Hz
AQ 4.1943040 sec
RG 12.7
DW 32.000 usec
DE 10.00 usec
TE 298.2 K
D1 1.00000000 sec
TD0 1
SFO1 600.3357031 MHz
NUC1 1H
P0 2.60 usec
P1 7.80 usec
PLW1 5.56960011 W
F2 - Processing parameters
SI 131072
SF 600.3300157 MHz
WDW EM
SSB 0
LB 0.20 Hz
GB 0
PC 4.00
17 16 15 14 13 12 11 ppm
Scale: 0.5152 ppm/cm, 309.3 Hz/cm
9.5 9.0 8.5 8.0 7.5 7.0 6.5 6.0 5.5 5.0 4.5 4.0 3.5 3.0 2.5 2.0 1.5 1.0 0.5 0.0 ppm
Scale: 0.2701 ppm/cm, 162.2 Hz/cm
22.44 0.33 22.60 3.63 15.11 0.31 22.33 8.06 5.19
TMS linewidth : 0.66 Hz

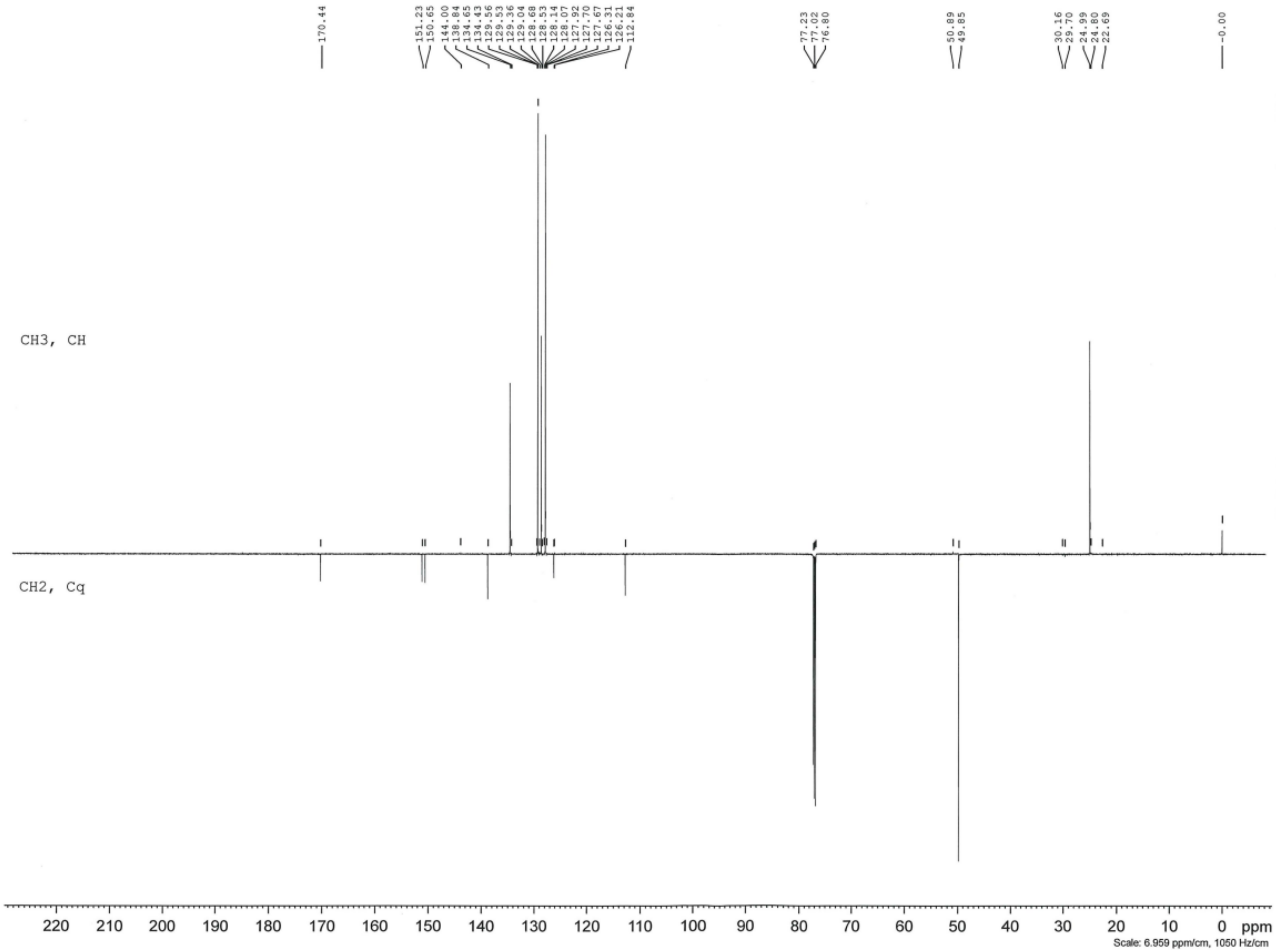


```
Current Data Parameters
NAME          079807
EXPNO             11
PROCNO             1

F2 - Acquisition Parameters
Date_       20231019
Time_          23.27 h
INSTRUM     Avance Neo
PROBHD  Z162398_0004 (
PULPROG     deptqgpsp
TD             65536
SOLVENT        CDCl3
NS              2048
DS                 0
SWH        35714.285 Hz
FIDRES      1.089913 Hz
AQ         0.9175040 sec
RG               101
DW            14.000 usec
DE             18.00 usec
TE             298.2 K
CNST2    145.0000000
D1        2.00000000 sec
D2        0.00344828 sec
D12       0.00002000 sec
D16       0.00020000 sec
TD0                1
SFO1     150.9697225 MHz
NUC1             13C
P1             12.00 usec
P13          2000.00 usec
PLW0               0 W
PLW1    117.19000244 W
SPNAM[5]  Crp60comp.4
SPOAL5         0.500
SPOFFS5            0 Hz
SPW5     25.78499985 W
SFO2     600.3324013 MHz
NUC2              1H
CNST12     1.5000000
CPDPRG[2     waltz64
P0             12.00 usec
P3              8.00 usec
P4             16.00 usec
PCPD2          80.00 usec
PLW2      5.55999994 W
PLW12     0.05560000 W
GPNAM[1]    SINE.100
GPZ1           31.00 %
GPNAM[2]    SINE.100
GPZ2           31.00 %
GPNAM[3]    SINE.100
GPZ3           31.00 %
P16          1000.00 usec

F2 - Processing parameters
SI             32768
SF       150.9530991 MHz
WDW               EM
SSB                0
LB              1.00 Hz
GB                 0
PC              1.40
```

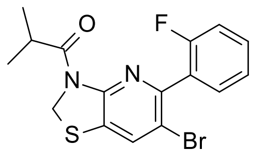

14b

1-[6-Bromo-5-(2-fluorophenyl)[1,3]thiazolo[4,5-*b*]pyridin-3(2*H*)-yl]-2-methylpropan-1-one (**14b**, 22 mg, 17%). ^{1}H NMR (600 MHz, $CDCl_3$): δ 1.14 (d, *J* = 6.9 Hz, 6H), 3.95–4.00 (m, 1H), 5.35 (s, 2H), 7.15–7.25 (m, 2H), 7.38–7.45 (m, 2H), 7.64–7.66 (m, 1H) ppm; ^{13}C NMR ($CDCl_3$, 151 MHz): δ 19.0 (CH_3), 32.8 (CH), 50.3 (CH_2), 114.8 (C), 115.8 (CH), 123.8 (CH), 127.3 (C), 127.6 (C), 130.6 (CH), 131.2 (CH), 133.9 (CH), 147.3 (C), 150.4 (C), 159.5 (d, C), 177.9 (C) ppm; HRMS (ESI, m/z): calcd. for $C_{16}H_{15}BrFN_2OS$, 381.0065 $[M+H]^+$; found 381.0072.

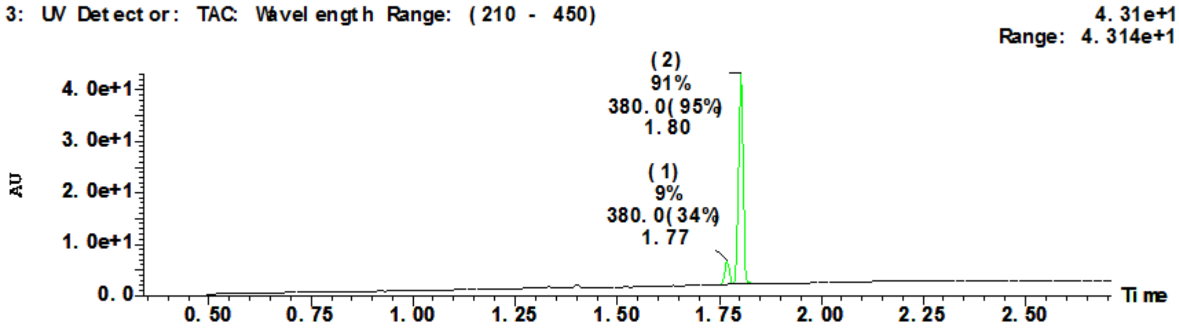

Peak Number	Time	Mass Found	Area %Total	RT LogP
1	1.77	380.00	9.41	4.32
2	1.80	380.00	90.59	4.46

Peak ID	Time	Mass Found
1	1.77	381

1: (Time: 1.77) Combine (219: 225-196: 208)

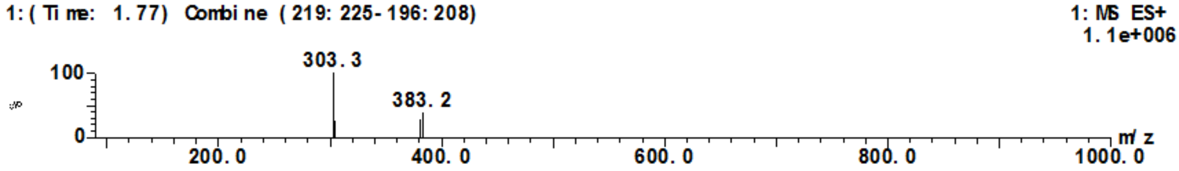

Peak ID	Time	Mass Found
1	1.77	Not Found

1: (Time: 1.77) Combine (219: 225-195: 208)

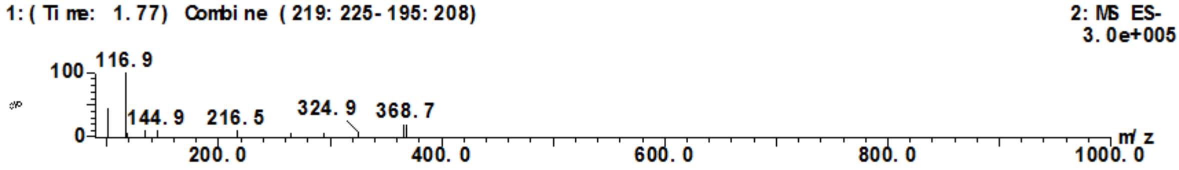

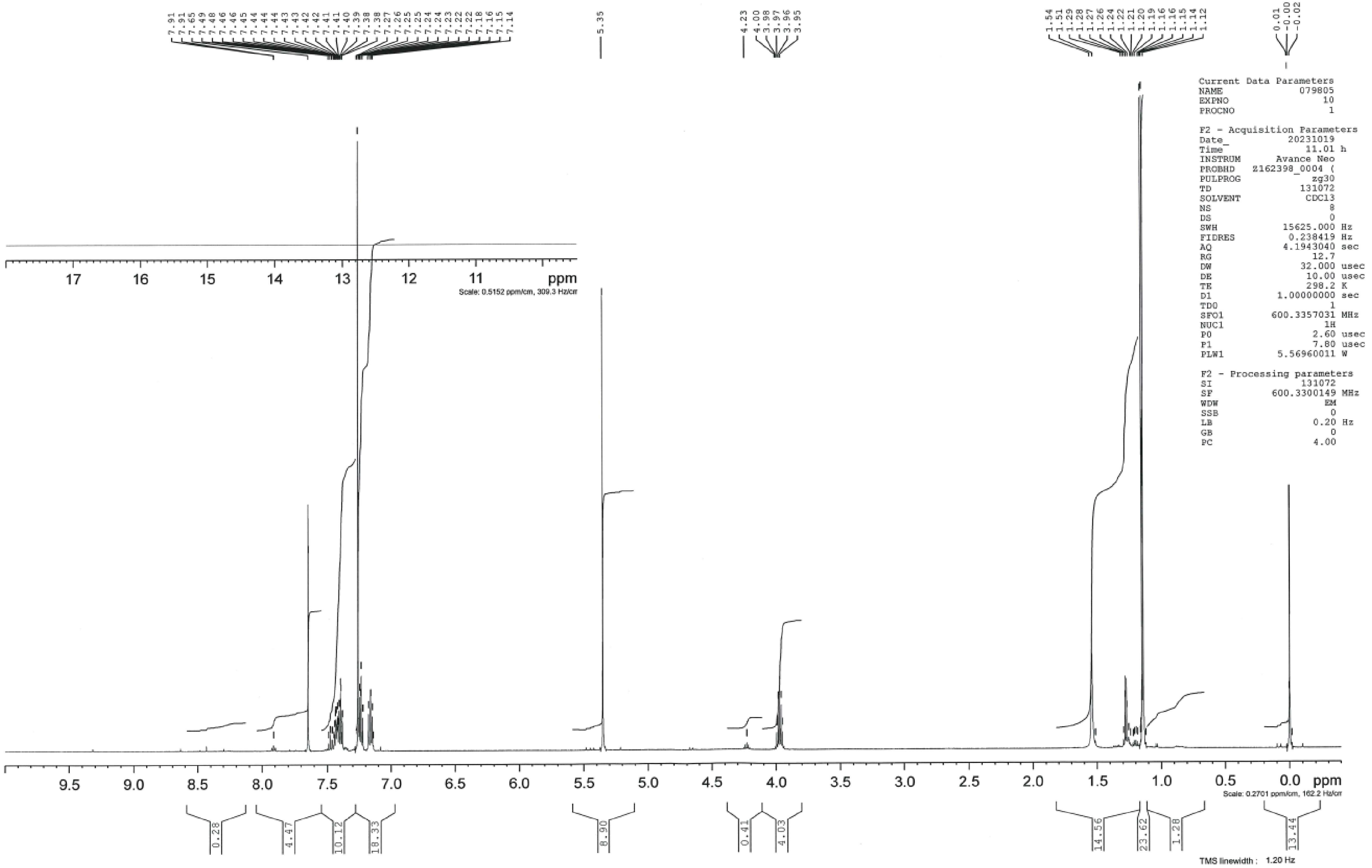
7.91
7.91
7.65
7.49
7.48
7.46
7.46
7.45
7.44
7.44
7.44
7.43
7.43
7.42
7.42
7.41
7.41
7.40
7.39
7.38
7.38
7.27
7.26
7.25
7.25
7.24
7.24
7.23
7.22
7.22
7.18
7.16
7.15
7.14
5.35
4.23
4.00
3.98
3.97
3.96
3.95
1.54
1.51
1.29
1.28
1.27
1.26
1.24
1.22
1.21
1.20
1.19
1.16
1.16
1.15
1.14
1.12
0.01
-0.00
-0.02
Current Data Parameters
NAME 079805
EXPNO 10
PROCNO 1
F2 - Acquisition Parameters
Date_ 20231019
Time 11.01 h
INSTRUM Avance Neo
PROBHD Z162398_0004 (
PULPROG zg30
TD 131072
SOLVENT CDCl3
NS 8
DS 0
SWH 15625.000 Hz
FIDRES 0.238419 Hz
AQ 4.1943040 sec
RG 12.7
DW 32.000 usec
DE 10.00 usec
TE 298.2 K
D1 1.00000000 sec
TD0 1
SFO1 600.3357031 MHz
NUC1 1H
P0 2.60 usec
P1 7.80 usec
PLW1 5.56960011 W
F2 - Processing parameters
SI 131072
SF 600.3300149 MHz
WDW EM
SSB 0
LB 0.20 Hz
GB 0
PC 4.00
17
16
15
14
13
12
11
ppm
Scale: 0.5152 ppm/cm, 309.3 Hz/cm
9.5
9.0
8.5
8.0
7.5
7.0
6.5
6.0
5.5
5.0
4.5
4.0
3.5
3.0
2.5
2.0
1.5
1.0
0.5
0.0
ppm
Scale: 0.2701 ppm/cm, 162.2 Hz/cm
0.28
4.47
10.12
18.33
8.90
0.41
4.03
14.56
23.62
1.28
13.44
TMS linewidth : 1.20 Hz

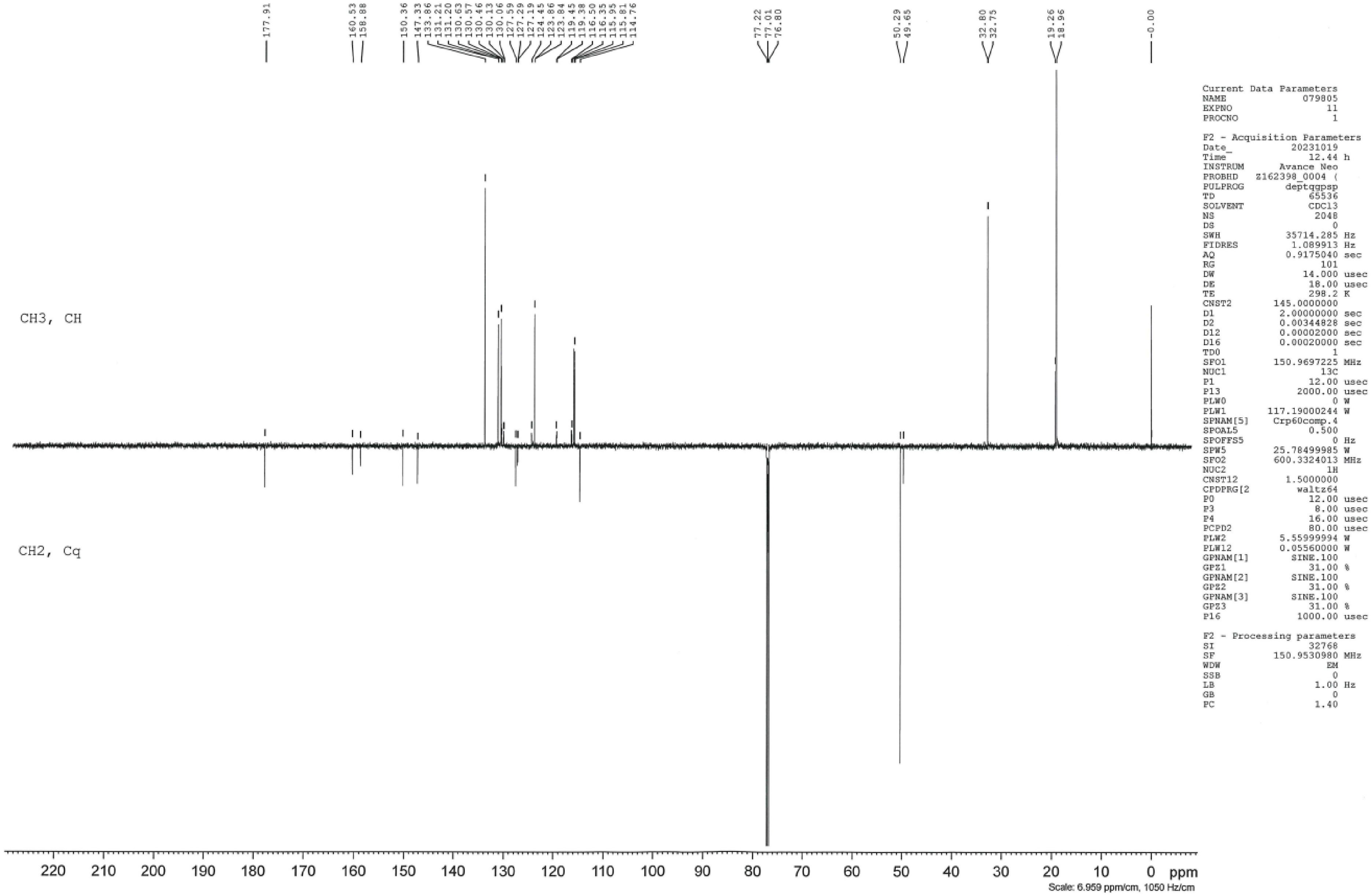
177.91
160.53
158.88
150.36
147.33
133.86
131.21
131.20
130.63
130.57
130.46
130.13
130.06
127.59
127.29
127.19
124.45
123.86
123.84
119.45
119.38
116.50
116.35
115.95
115.81
114.76
77.22
77.01
76.80
50.29
49.65
32.80
32.75
19.26
18.96
-0.00
CH3, CH
CH2, Cq
Current Data Parameters
NAME 079805
EXPNO 11
PROCNO 1
F2 - Acquisition Parameters
Date_ 20231019
Time_ 12.44 h
INSTRUM Avance Neo
PROBHD Z162398_0004 (
PULPROG deptqgpsp
TD 65536
SOLVENT CDCl3
NS 2048
DS 0
SWH 35714.285 Hz
FIDRES 1.089913 Hz
AQ 0.9175040 sec
RG 101
DW 14.000 usec
DE 18.00 usec
TE 298.2 K
CNST2 145.0000000
D1 2.00000000 sec
D2 0.00344828 sec
D12 0.00002000 sec
D16 0.00020000 sec
TD0 1
SFO1 150.9697225 MHz
NUC1 13C
P1 12.00 usec
P13 2000.00 usec
PLW0 0 W
PLW1 117.19000244 W
SPNAM[5] Crp60comp.4
SPOAL5 0.500
SPOFFS5 0 Hz
SPW5 25.78499985 W
SFO2 600.3324013 MHz
NUC2 1H
CNST12 1.5000000
CPDPRG[2 waltz64
P0 12.00 usec
P3 8.00 usec
P4 16.00 usec
PCPD2 80.00 usec
PLW2 5.55999994 W
PLW12 0.05560000 W
GPNAM[1] SINE.100
GPZ1 31.00 %
GPNAM[2] SINE.100
GPZ2 31.00 %
GPNAM[3] SINE.100
GPZ3 31.00 %
P16 1000.00 usec
F2 - Processing parameters
SI 32768
SF 150.9530980 MHz
WDW EM
SSB 0
LB 1.00 Hz
GB 0
PC 1.40
220 210 200 190 180 170 160 150 140 130 120 110 100 90 80 70 60 50 40 30 20 10 0 ppm
Scale: 6.959 ppm/cm, 1050 Hz/cm

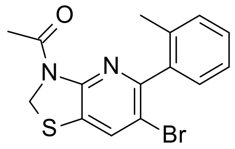

14c

1-[6-Bromo-5-(2-methylphenyl)[1,3]thiazolo[4,5-*b*]pyridin-3(2*H*)-yl]ethanone (**14c**, 51 mg, 73%). ^{1}H NMR (600 MHz, CDCl$_3$): δ 2.20–2.23 (m, 3H), 2.57 (s, 3H), 5.36–5.43 (m, 2H), 7.21–7.23 (m, 1H), 7.28–7.32 (m, 2H), 7.34–7.37 (m, 1H), 7.67–7.69 (m, 1H) ppm; ^{13}C NMR (CDCl$_3$, 151 MHz): δ 19.6 (CH$_3$), 24.8 (CH$_3$), 49.8 (CH$_2$), 114.6 (C), 125.5 (CH), 126.5 (C), 128.6 (CH), 129.1 (CH), 130.1 (CH), 133.8 (CH), 135.9 (C), 139.1 (C), 150.4 (C), 152.7 (C), 170.5 (C) ppm; HRMS (ESI, m/z): calcd. for $C_{15}H_{14}BrN_2OS$, 349.0025 [M+H]$^+$; found 349.0010.

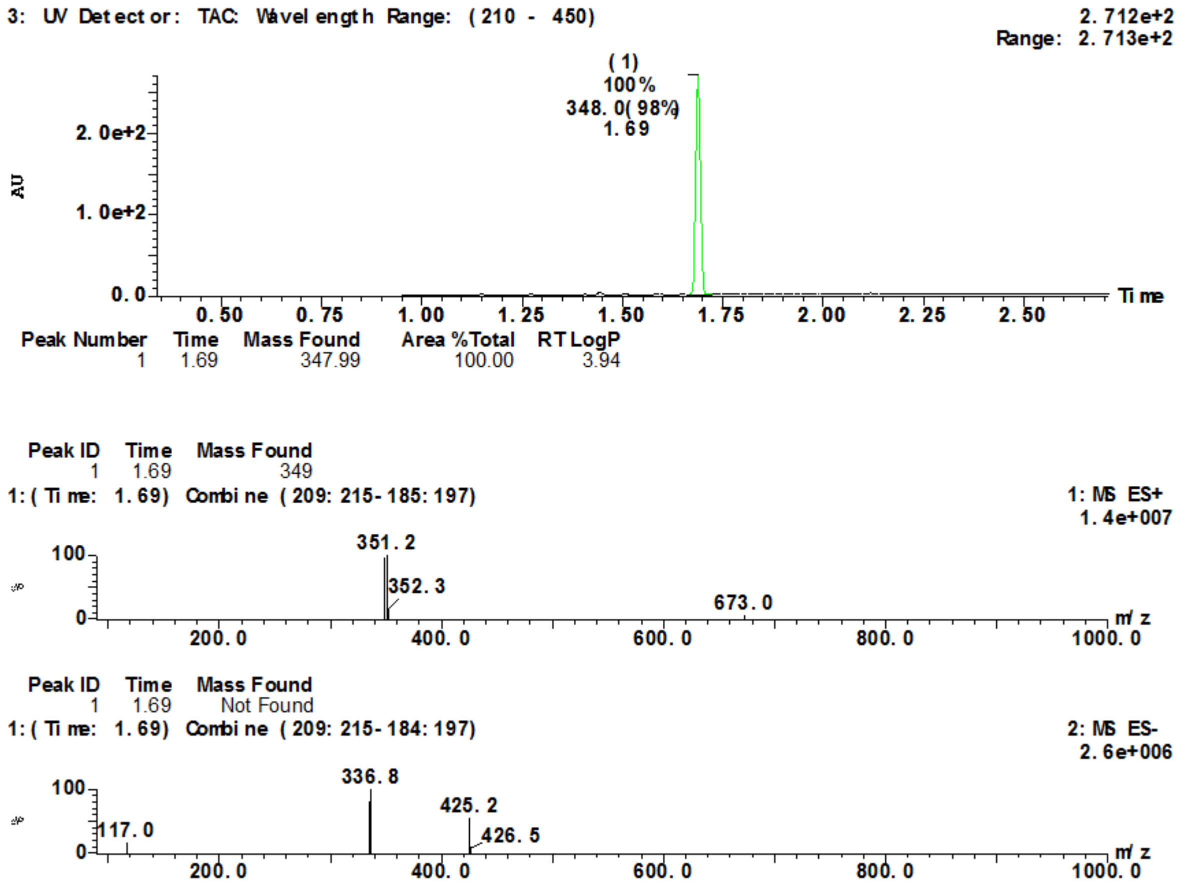

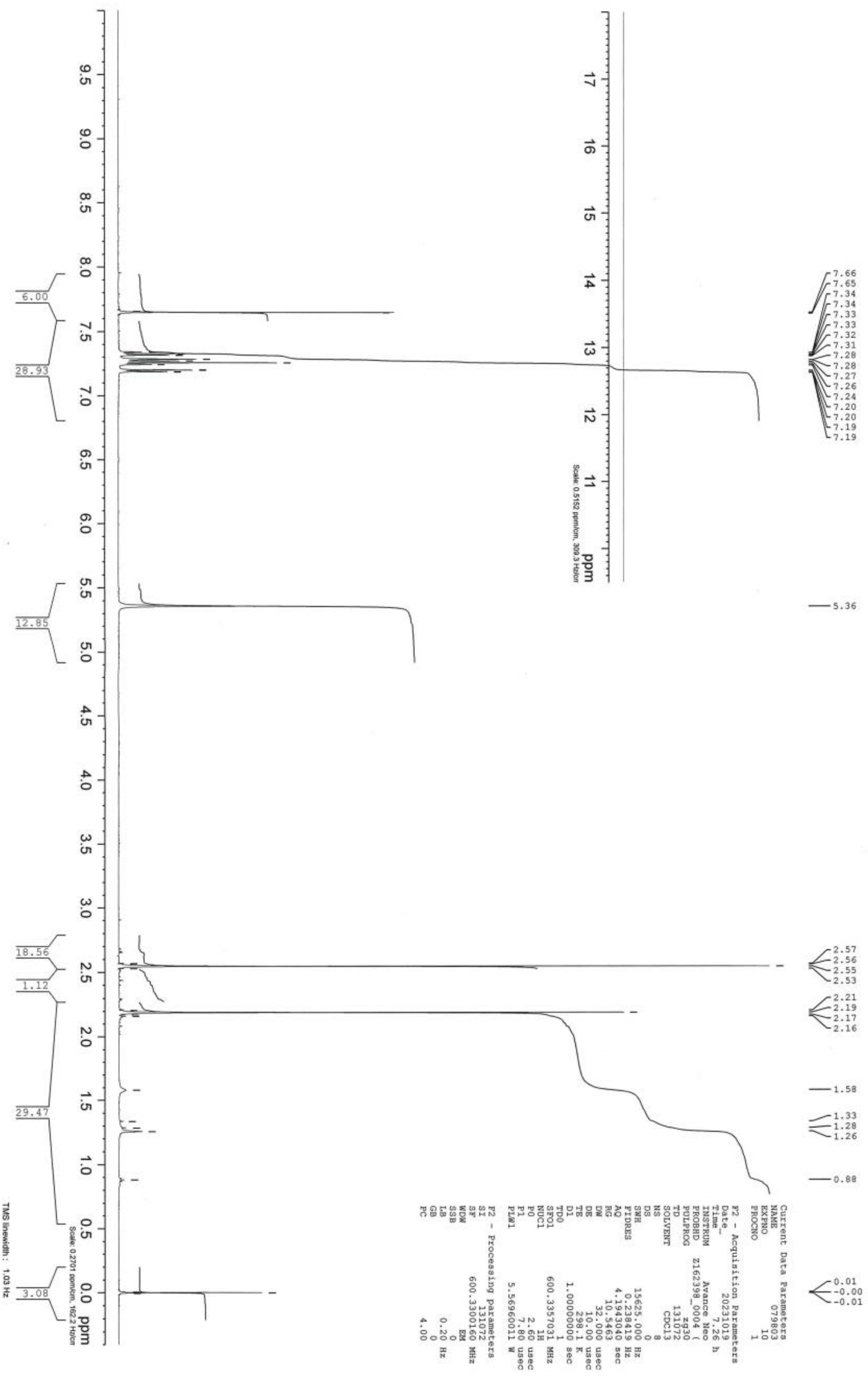
7.66
7.65
7.34
7.34
7.33
7.33
7.32
7.31
7.28
7.28
7.27
7.26
7.24
7.20
7.20
7.19
7.19
5.36
2.57
2.56
2.55
2.53
2.21
2.19
2.17
2.16
1.58
1.33
1.28
1.26
0.88
0.01
-0.00
-0.01
6.00
28.93
12.85
18.56
1.12
29.47
3.08
ppm
Scale: 0.2701 ppm/cm, 162.2 Hz/cm
TMS linewidth : 1.03 Hz
Scale: 0.5152 ppm/cm, 309.3 Hz/cm
Current Data Parameters
NAME 079803
EXPNO 10
PROCNO 1
F2 - Acquisition Parameters
Date_ 20231019
Time_ 7.26 h
INSTRUM Avance Neo
PROBHD Z162398_0004 (
PULPROG zg30
TD 131072
SOLVENT CDCl3
NS 8
DS 0
SWH 15625.000 Hz
FIDRES 0.238419 Hz
AQ 4.1943040 sec
RG 10.5463
DW 32.000 usec
DE 10.00 usec
TE 298.1 K
D1 1.00000000 sec
TD0 1
SFO1 600.3357031 MHz
NUC1 1H
P0 2.60 usec
P1 7.80 usec
PLW1 5.56960011 W
F2 - Processing parameters
SI 131072
SF 600.3300160 MHz
WDW EM
SSB 0
LB 0.20 Hz
GB 0
PC 4.00

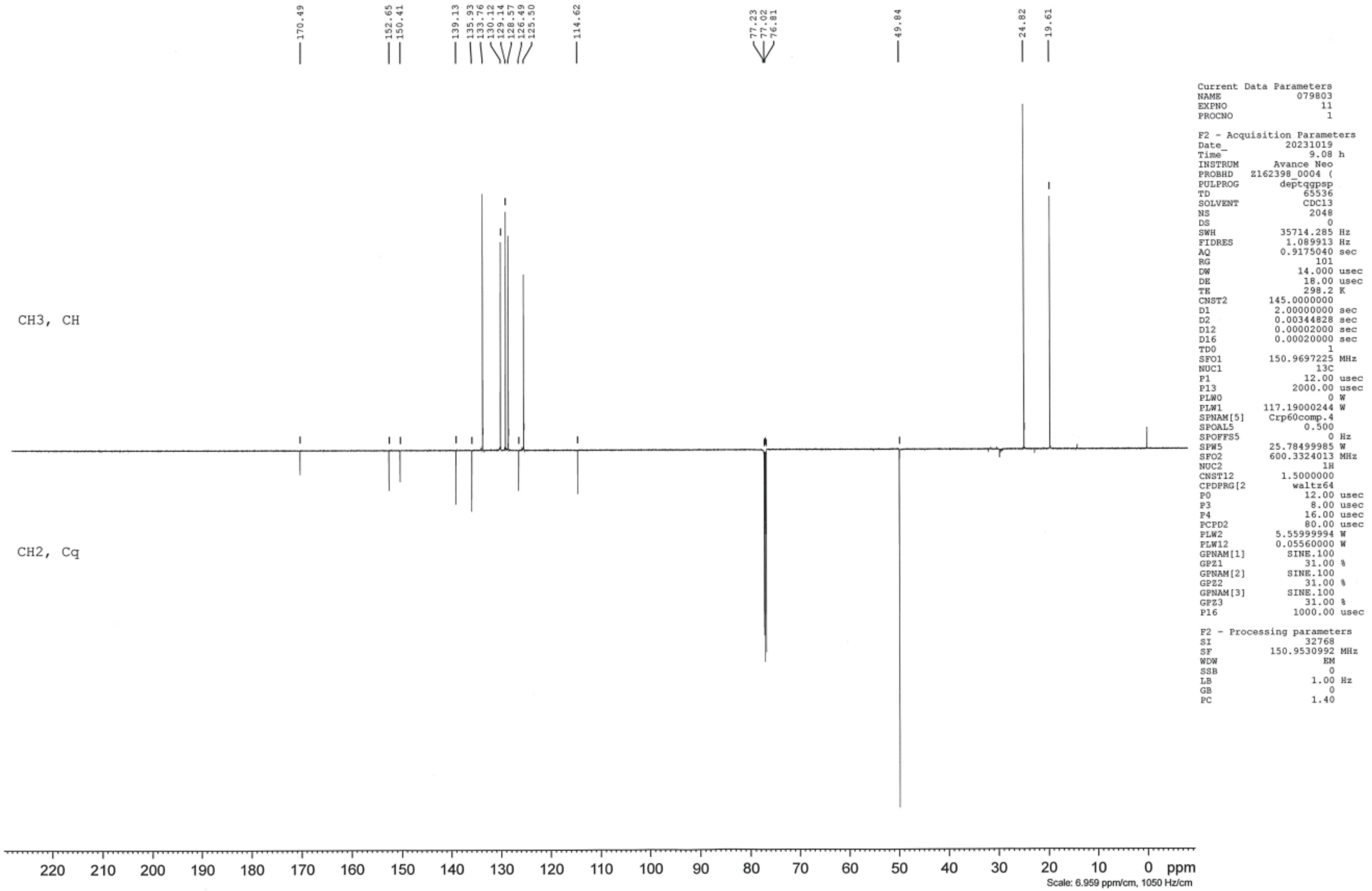
170.49
152.65
150.41
139.13
135.93
133.76
130.12
129.14
128.57
126.49
125.50
114.62
77.23
77.02
76.81
49.84
24.82
19.61
CH3, CH
CH2, Cq
220 210 200 190 180 170 160 150 140 130 120 110 100 90 80 70 60 50 40 30 20 10 0 ppm
Scale: 6.959 ppm/cm, 1050 Hz/cm
Current Data Parameters
NAME 079803
EXPNO 11
PROCNO 1
F2 - Acquisition Parameters
Date_ 20231019
Time 9.08 h
INSTRUM Avance Neo
PROBHD Z162398_0004 (
PULPROG deptqgpsp
TD 65536
SOLVENT CDCl3
NS 2048
DS 0
SWH 35714.285 Hz
FIDRES 1.089913 Hz
AQ 0.9175040 sec
RG 101
DW 14.000 usec
DE 18.00 usec
TE 298.2 K
CNST2 145.0000000
D1 2.00000000 sec
D2 0.00344828 sec
D12 0.00002000 sec
D16 0.00020000 sec
TD0 1
SFO1 150.9697225 MHz
NUC1 13C
P1 12.00 usec
P13 2000.00 usec
PLW0 0 W
PLW1 117.19000244 W
SPNAM[5] Crp60comp.4
SPOAL5 0.500
SPOFFS5 0 Hz
SPW5 25.78499985 W
SFO2 600.3324013 MHz
NUC2 1H
CNST12 1.5000000
CPDPRG[2 waltz64
P0 12.00 usec
P3 8.00 usec
P4 16.00 usec
PCPD2 80.00 usec
PLW2 5.55999994 W
PLW12 0.05560000 W
GPNAM[1] SINE.100
GPZ1 31.00 %
GPNAM[2] SINE.100
GPZ2 31.00 %
GPNAM[3] SINE.100
GPZ3 31.00 %
P16 1000.00 usec
F2 - Processing parameters
SI 32768
SF 150.9530992 MHz
WDW EM
SSB 0
LB 1.00 Hz
GB 0
PC 1.40

1.2.7. General procedure for the synthesis of N-acylated 6-methyl-2,3-dihydro[1,3]thiazolo[4,5-*b*]pyridines 16a–f

The corresponding acyl chloride (0.31 mmol, 1.1 equiv) and triethylamine (0.09 mL, 0.63 mmol, 2.2 equiv) were added to a stirred solution of the corresponding 6-methyl-5-phenyl-2,3-dihydro[1,3]thiazolo[4,5-*b*]pyridine **7a–c** (0.28 mmol, 1.00 equiv) in absolute DCM (5 mL). The resulting reaction mixture was stirred at room temperature for 30–120 min, followed by dilution with DCM and water, and subsequent extraction and phase separation. The aqueous layer was thoroughly extracted with DCM, and the combined organic layer was dried over anhydrous $MgSO_4$, filtered, and concentrated under reduced pressure. The remaining crude product was purified via column chromatography (gradient ethyl acetate/heptane) to afford the corresponding desired target compound **16a**–**f**.

1.2.8. Characterization of N-acylated 6-methyl-2,3-dihydro[1,3]thiazolo[4,5-*b*]pyridines 16a–f

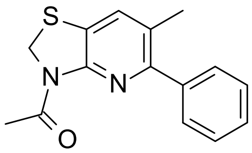

16a

1-(6-Methyl-5-phenyl[1,3]thiazolo[4,5-*b*]pyridin-3(2*H*)-yl)ethanone (**16a**, 150 mg, 94%). ^{1}H NMR (600 MHz, $CDCl_3$): δ 2.32 (s, 3H), 2.68 (s, 3H), 5.32 (s, 2H), 7.33 (s, 1H), 7.36–7.39 (m, 1H), 7.42–7.45 (m, 2H), 7.51–7.53 (m, 2H) ppm; ^{13}C NMR ($CDCl_3$, 151 MHz): δ 19.7 (CH_3), 24.8 (CH_3), 49.2 (CH_2), 124.1 (C), 125.9 (C), 127.9 (CH), 128.1 (CH), 129.0 (CH), 139.9 (C), 149.6 (C), 151.8 (C), 170.5 (C) ppm; HRMS (ESI, m/z): calcd. for $C_{15}H_{15}N_2OS$, 271.0906 $[M+H]^+$; found 271.0905.

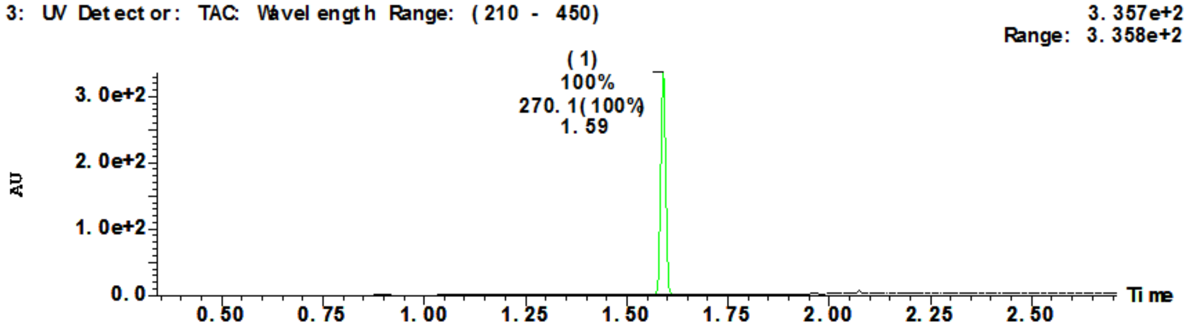

Peak Number	Time	Mass Found	Area %Total	RT LogP
1	1.59	270.08	100.00	3.46

Peak ID	Time	Mass Found
1	1.59	271

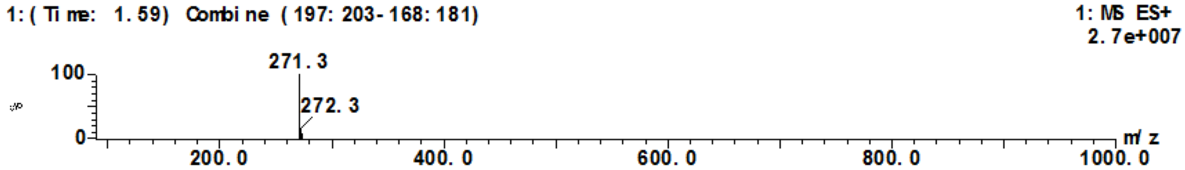

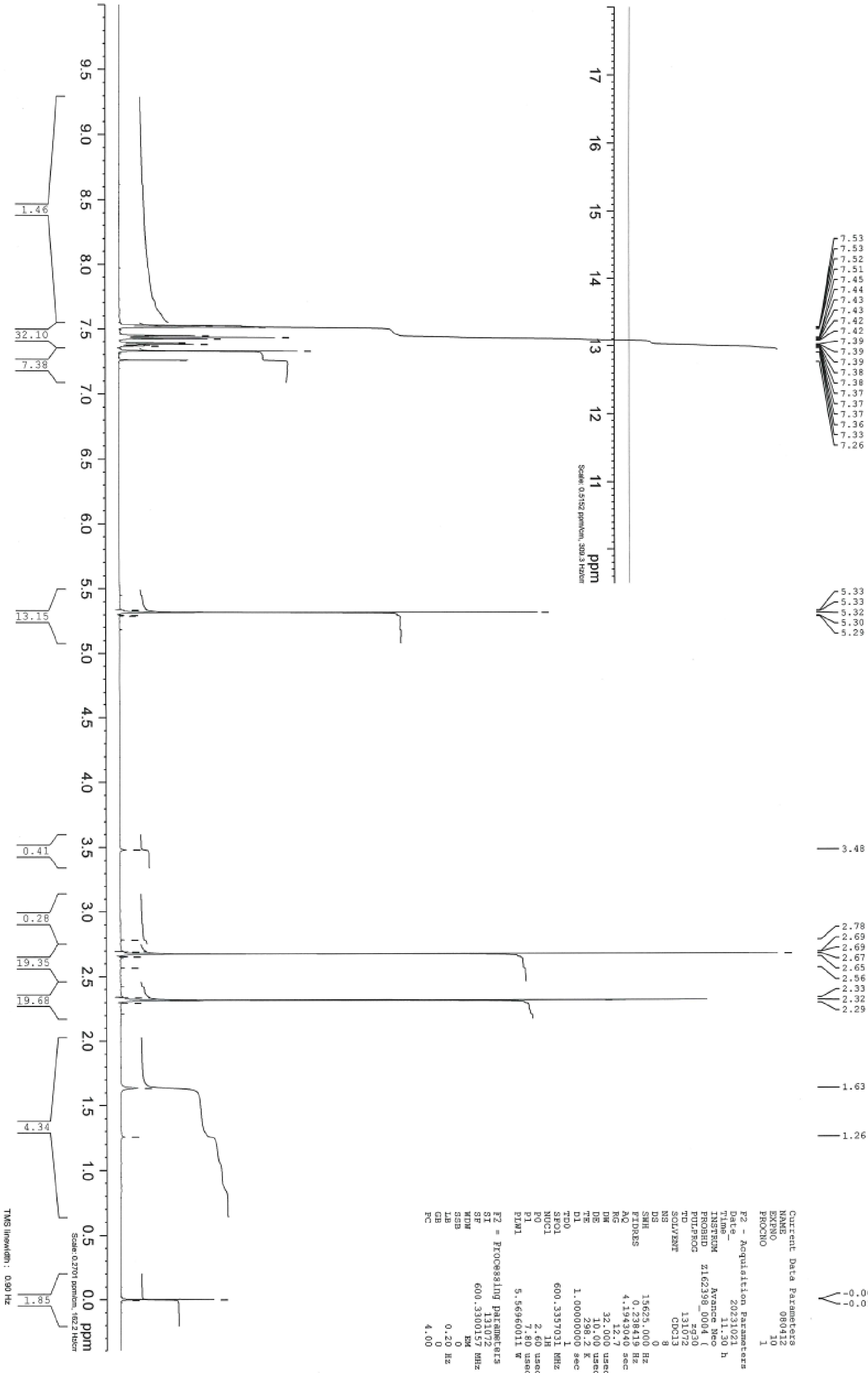
Current Data Parameters
NAME 080412
EXPNO 10
PROCNO 1
F2 - Acquisition Parameters
Date_ 20231021
Time_ 11.30 h
INSTRUM Avance Neo
PROBHD Z162398_0004 (
PULPROG zg30
TD 131072
SOLVENT CDCl3
NS 8
DS 0
SWH 15625.000 Hz
FIDRES 0.238419 Hz
AQ 4.1943040 sec
RG 12.7
DW 32.000 usec
DE 10.00 usec
TE 298.2 K
D1 1.00000000 sec
TD0 1
SFO1 600.3357031 MHz
NUC1 1H
P0 2.60 usec
P1 7.80 usec
PLW1 5.56960011 W
F2 - Processing parameters
SI 131072
SF 600.3300157 MHz
WDW EM
SSB 0
LB 0.20 Hz
GB 0
PC 4.00
7.53
7.53
7.52
7.51
7.45
7.44
7.43
7.43
7.42
7.42
7.39
7.39
7.39
7.38
7.38
7.37
7.37
7.37
7.36
7.33
7.26
5.33
5.33
5.32
5.30
5.29
3.48
2.78
2.69
2.69
2.67
2.65
2.56
2.33
2.32
2.29
1.63
1.26
-0.00
-0.01
1.46
32.10
7.38
13.15
0.41
0.28
19.35
19.68
4.34
1.85
ppm
TMS linewidth: 0.90 Hz
Scale: 0.2701 ppm/cm, 162.2 Hz/cm
Scale: 0.5152 ppm/cm, 309.3 Hz/cm

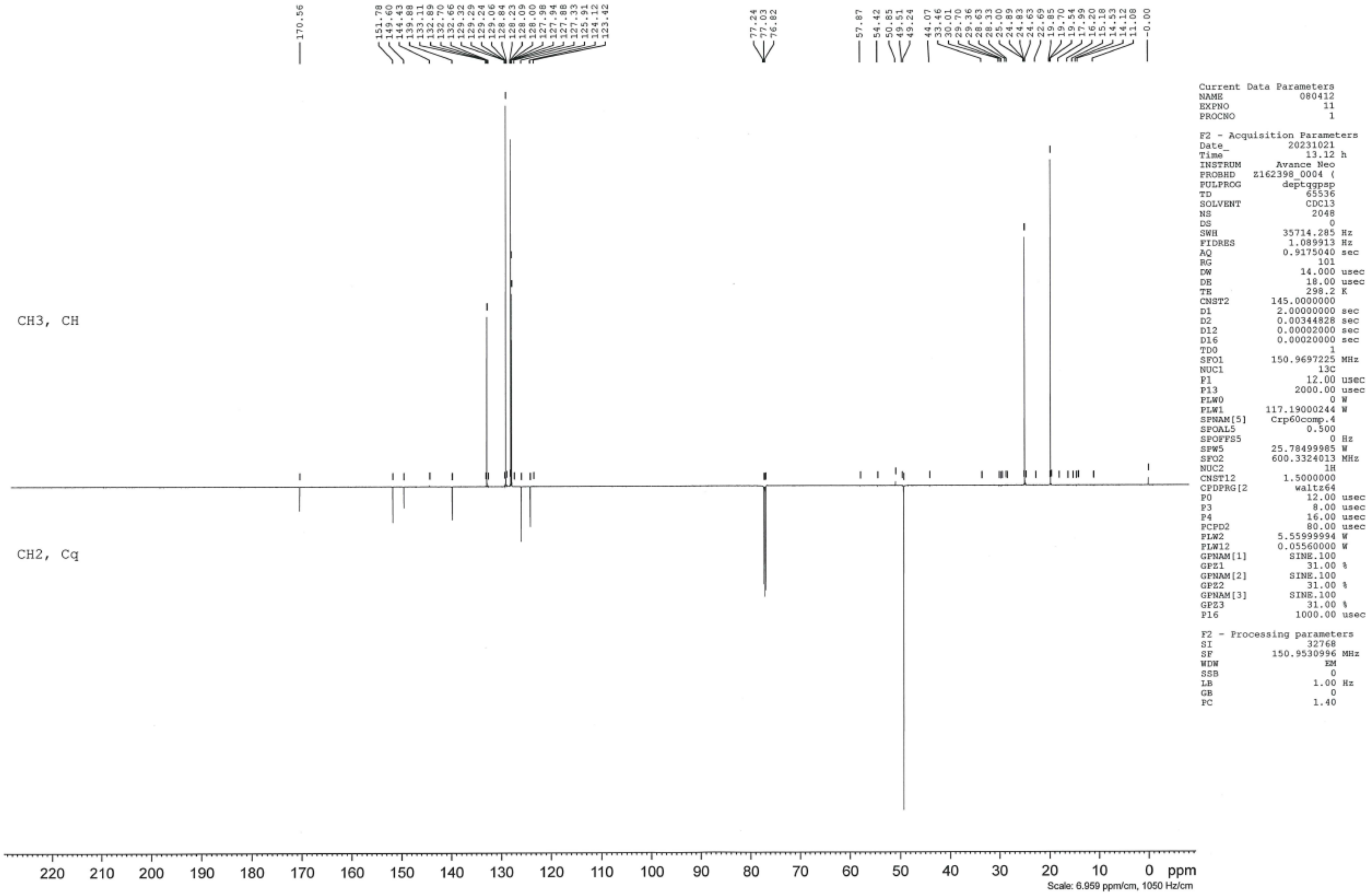
170.56
151.78
149.60
144.43
139.88
133.11
132.89
132.70
132.66
129.32
129.29
129.24
129.06
128.84
128.23
128.09
128.00
127.98
127.94
127.88
127.33
125.91
124.12
123.42
77.24
77.03
76.82
57.87
54.42
50.85
49.51
49.24
44.07
33.46
30.01
29.70
29.36
28.63
28.33
25.00
24.89
24.83
24.63
22.69
19.85
19.70
19.54
17.99
16.20
15.18
14.53
14.12
11.08
-0.00
CH3, CH
CH2, Cq
Current Data Parameters
NAME 080412
EXPNO 11
PROCNO 1
F2 - Acquisition Parameters
Date_ 20231021
Time 13.12 h
INSTRUM Avance Neo
PROBHD Z162398_0004 (
PULPROG deptqgpsp
TD 65536
SOLVENT CDCl3
NS 2048
DS 0
SWH 35714.285 Hz
FIDRES 1.089913 Hz
AQ 0.9175040 sec
RG 101
DW 14.000 usec
DE 18.00 usec
TE 298.2 K
CNST2 145.0000000
D1 2.00000000 sec
D2 0.00344828 sec
D12 0.00002000 sec
D16 0.00020000 sec
TD0 1
SFO1 150.9697225 MHz
NUC1 13C
P1 12.00 usec
P13 2000.00 usec
PLW0 0 W
PLW1 117.19000244 W
SPNAM[5] Crp60comp.4
SPOAL5 0.500
SPOFFS5 0 Hz
SPW5 25.78499985 W
SFO2 600.3324013 MHz
NUC2 1H
CNST12 1.5000000
CPDPRG[2 waltz64
P0 12.00 usec
P3 8.00 usec
P4 16.00 usec
PCPD2 80.00 usec
PLW2 5.55999994 W
PLW12 0.05560000 W
GPNAM[1] SINE.100
GPZ1 31.00 %
GPNAM[2] SINE.100
GPZ2 31.00 %
GPNAM[3] SINE.100
GPZ3 31.00 %
P16 1000.00 usec
F2 - Processing parameters
SI 32768
SF 150.9530996 MHz
WDW EM
SSB 0
LB 1.00 Hz
GB 0
PC 1.40
220 210 200 190 180 170 160 150 140 130 120 110 100 90 80 70 60 50 40 30 20 10 0 ppm
Scale: 6.959 ppm/cm, 1050 Hz/cm

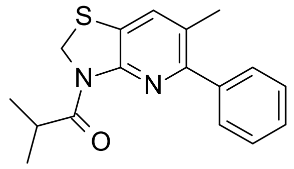

16b

2-Methyl-1-(6-methyl-5-phenyl[1,3]thiazolo[4,5-*b*]pyridin-3(2*H*)-yl)propan-1-one (**16b**, 130 mg, 72%). ^{1}H NMR (600 MHz, $CDCl_3$): δ 1.20 (d, *J* = 6.7 Hz, 6H), 2.32 (s, 2H), 4.15–4.19 (m, 1H), 5.32 (s, 2H), 7.33 (s, 1H), 7.37–7.39 (m, 1H), 7.43–7.45 (m, 2H), 7.51–7.53 (m, 2H) ppm; ^{13}C NMR ($CDCl_3$, 151 MHz): δ 19.2 (2 × CH_3), 19.7 (CH_3), 32.6 (CH), 49.6 (CH_2), 124.3 (C), 125.8 (C), 127.8 (CH), 128.0 (CH), 129.0 (CH), 132.9 (CH), 139.9 (C), 149.4 (C), 151.8 (C), 177.9 (C) ppm; HRMS (ESI, m/z): calcd. for $C_{17}H_{19}N_2OS$, 299.1218 $[M+H]^+$; found 299.1218.

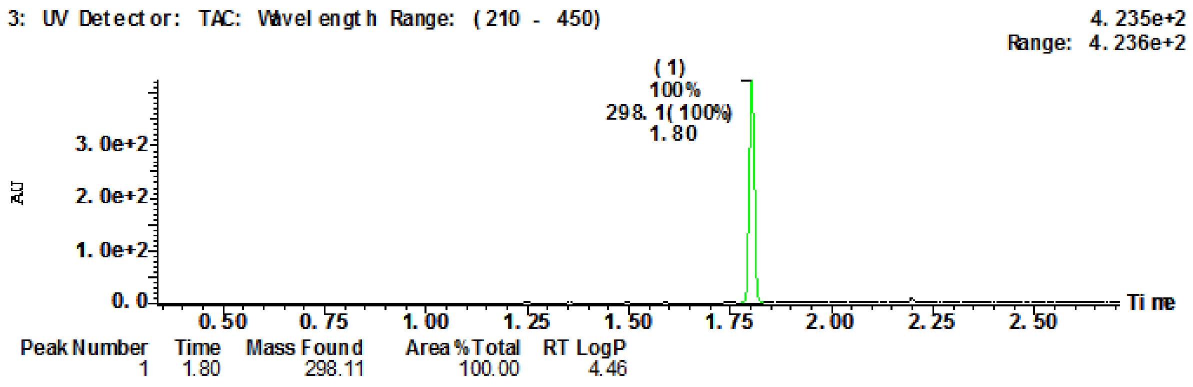

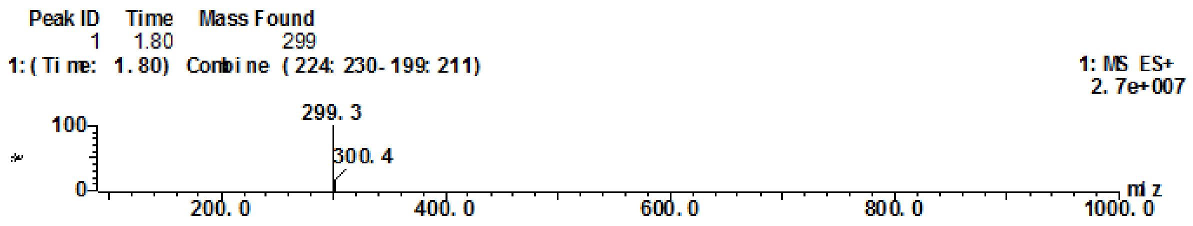

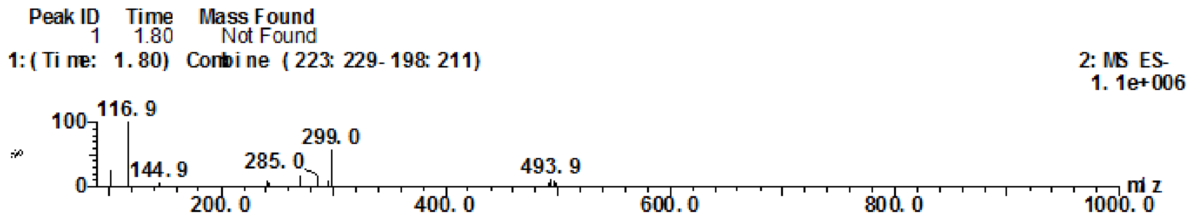

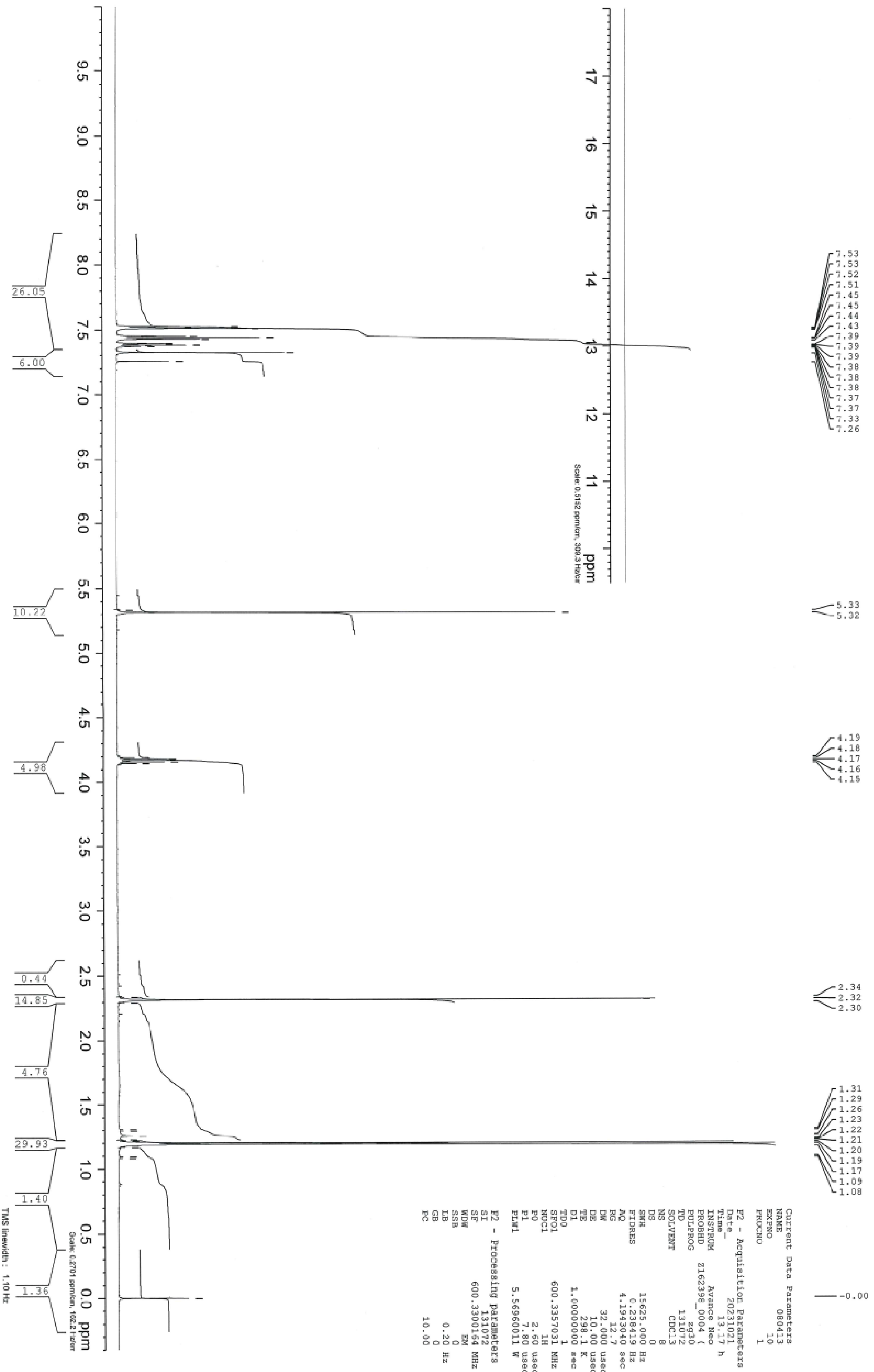

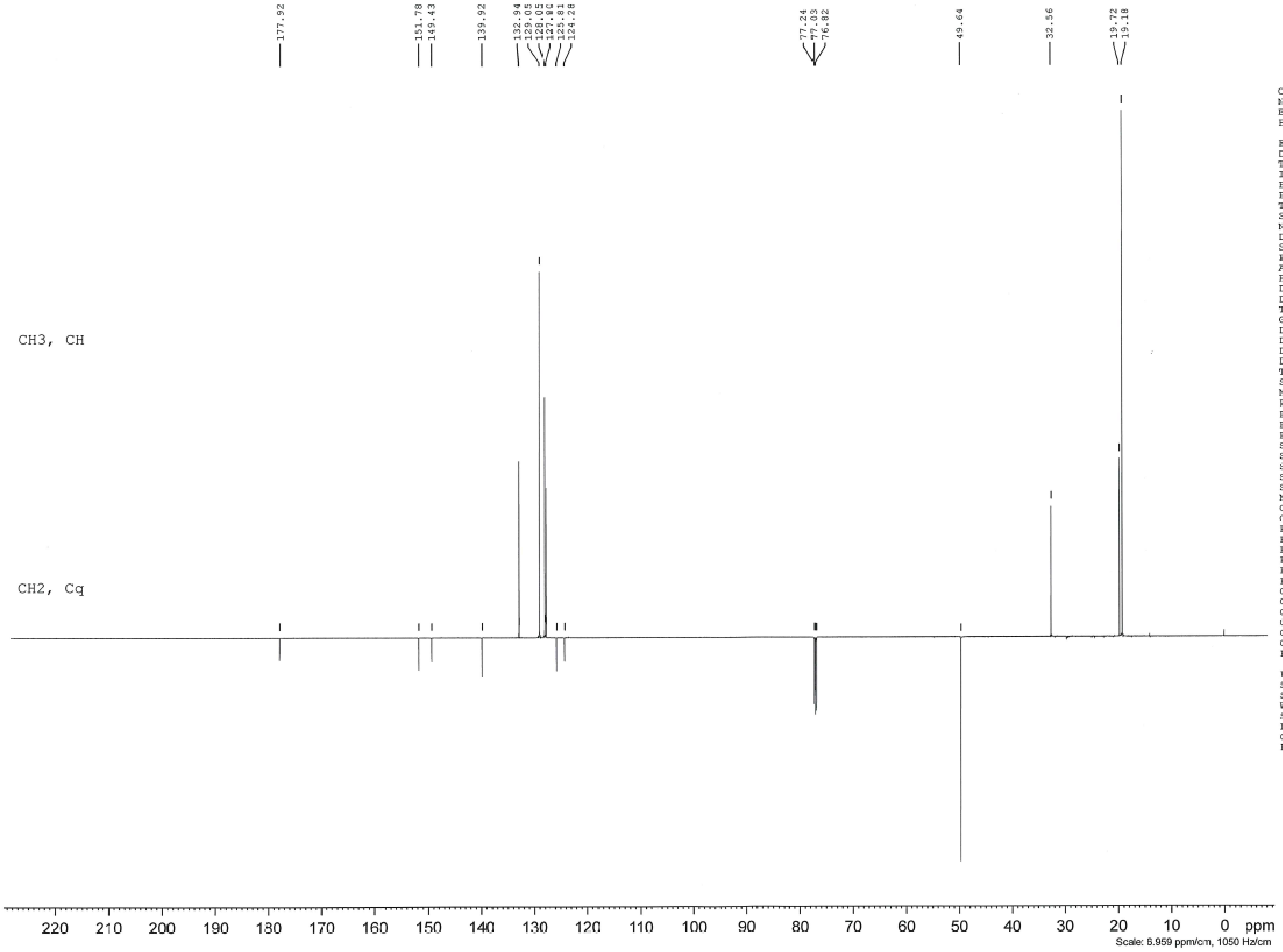

Current Data Parameters	
NAME	080413
EXPNO	11
PROCNO	1
F2 - Acquisition Parameters	
Date_	20231021
Time	15.00 h
INSTRUM	Avance Neo
PROBHD	Z162398_0004 (
PULPROG	deptqgpsp
TD	65536
SOLVENT	CDCl3
NS	2048
DS	0
SWH	35714.285 Hz
FIDRES	1.089913 Hz
AQ	0.9175040 sec
RG	101
DW	14.000 usec
DE	18.00 usec
TE	298.1 K
CNST2	145.0000000
D1	2.00000000 sec
D2	0.00344828 sec
D12	0.00002000 sec
D16	0.00020000 sec
TD0	1
SFO1	150.9697225 MHz
NUC1	13C
P1	12.00 usec
P13	2000.00 usec
PLW0	0 W
PLW1	117.19000244 W
SPNAM[5]	Crp60comp.4
SPOAL5	0.500
SPOFFS5	0 Hz
SPW5	25.78499985 W
SFO2	600.3324013 MHz
NUC2	1H
CNST12	1.5000000
CPDPRG[2	waltz64
P0	12.00 usec
P3	8.00 usec
P4	16.00 usec
PCPD2	80.00 usec
PLW2	5.55999994 W
PLW12	0.05560000 W
GPNAM[1]	SINE.100
GPZ1	31.00 %
GPNAM[2]	SINE.100
GPZ2	31.00 %
GPNAM[3]	SINE.100
GPZ3	31.00 %
P16	1000.00 usec
F2 - Processing parameters	
SI	32768
SF	150.9531001 MHz
WDW	EM
SSB	0
LB	1.00 Hz
GB	0
PC	1.40

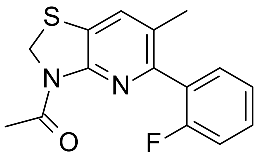

16c

1-[5-(2-Fluorophenyl)-6-methyl[1,3]thiazolo[4,5-*b*]pyridin-3(2*H*)-yl]ethanone (**16c**, 150 mg, 84%). ^{1}H NMR (600 MHz, $CDCl_3$): δ 2.16 (d, *J* = 2.2 Hz, 3H), 2.61 (s, 2H), 5.32 (s, 2H), 7.12–7.15 (m, 1H), 7.21–7.24 (m, 1H), 7.34 (s, 1H), 7.37–7.49 (m, 2H) ppm; ^{13}C NMR ($CDCl_3$, 151 MHz): δ 18.41 (CH_3), 18.44 (CH_3), 24.7 (CH_3), 49.3 (CH_2), 115.7 (CH), 115.8 (CH), 124.09 (CH), 124.12 (CH), 125.2 (C), 127.8 (C), 129.9 (CH), 130.0 (CH), 131.37 (CH), 131.40 (CH), 132.1 (CH), 147.0 (C), 149.6 (C), 158.9 (C), 160.6 (C), 170.6 (C) ppm; HRMS (ESI, m/z): calcd. for $C_{15}H_{14}FN_2OS$, 289.0811 $[M+H]^+$; found 289.0806.

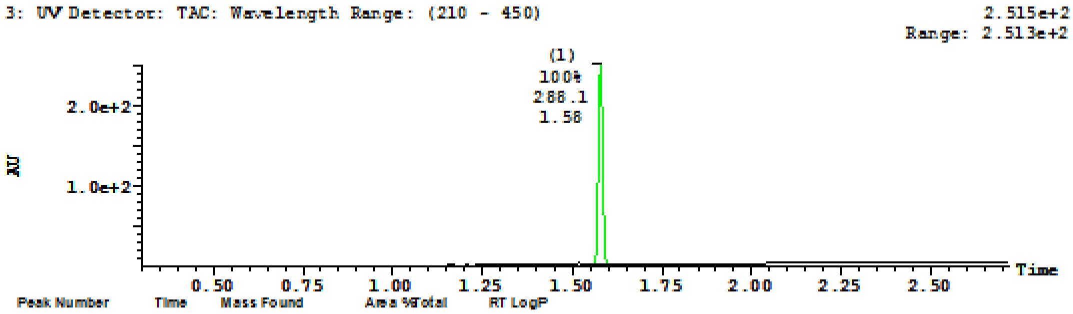

Peak Number	Time	Mass Found	Area %Total	RT LogP
1	1.58	288.0733	100.00	3.42

Peak ID	Time	Mass Found
1	1.58	289

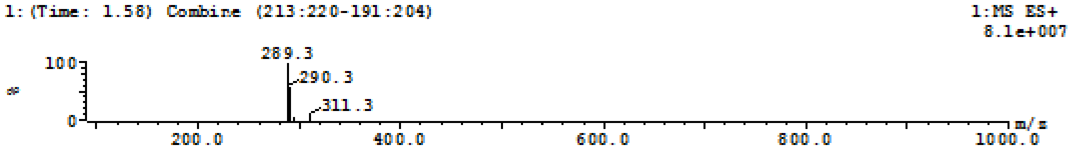

Peak ID	Time	Mass Found
1	1.58	Not Found

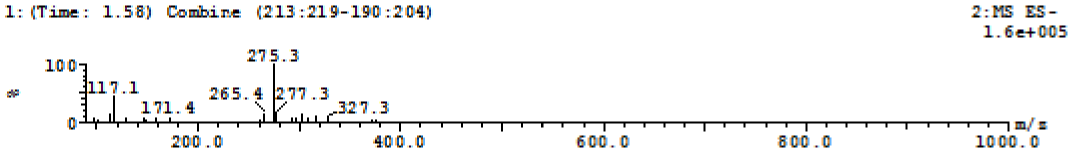

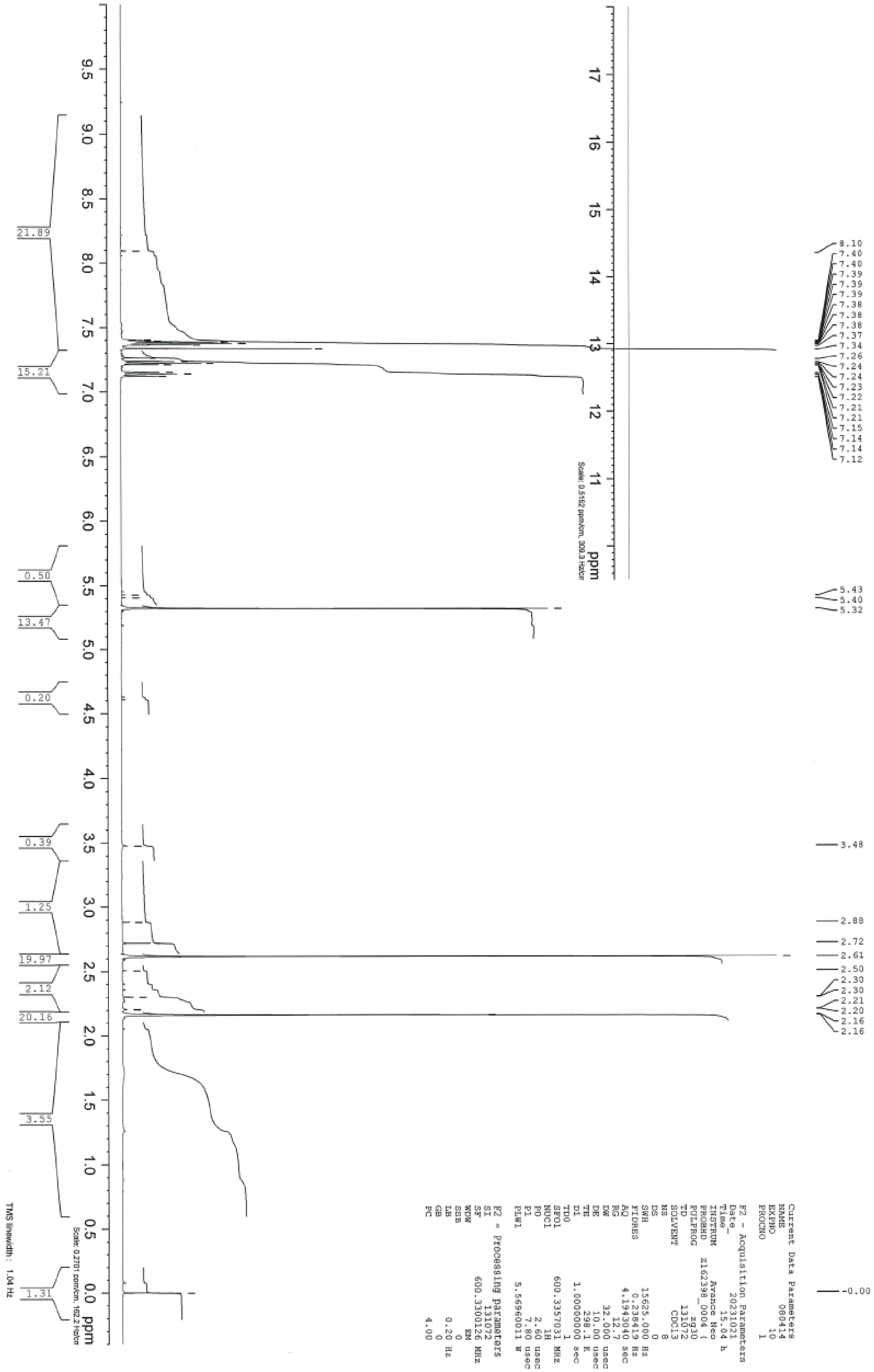
Current Data Parameters
NAME 080414
EXPNO 10
PROCNO 1
F2 - Acquisition Parameters
Date_ 20231021
Time 15.04 h
INSTRUM Avance Neo
PROBHD Z162398_0004 (
PULPROG zg30
TD 131072
SOLVENT CDCl3
NS 8
DS 0
SWH 15625.000 Hz
FIDRES 0.238419 Hz
AQ 4.1943040 sec
RG 12.7
DW 32.000 usec
DE 10.00 usec
TE 298.1 K
D1 1.00000000 sec
TD0 1
SFO1 600.3357031 MHz
NUC1 1H
P0 2.60 usec
P1 7.80 usec
PLW1 5.56960011 W
F2 - Processing parameters
SI 131072
SF 600.3300126 MHz
WDW EM
SSB 0
LB 0.20 Hz
GB 0
PC 4.00
ppm
Scale: 0.2701 ppm/cm, 162.2 Hz/cm
Scale: 0.5152 ppm/cm, 309.3 Hz/cm
TMS linewidth: 1.04 Hz

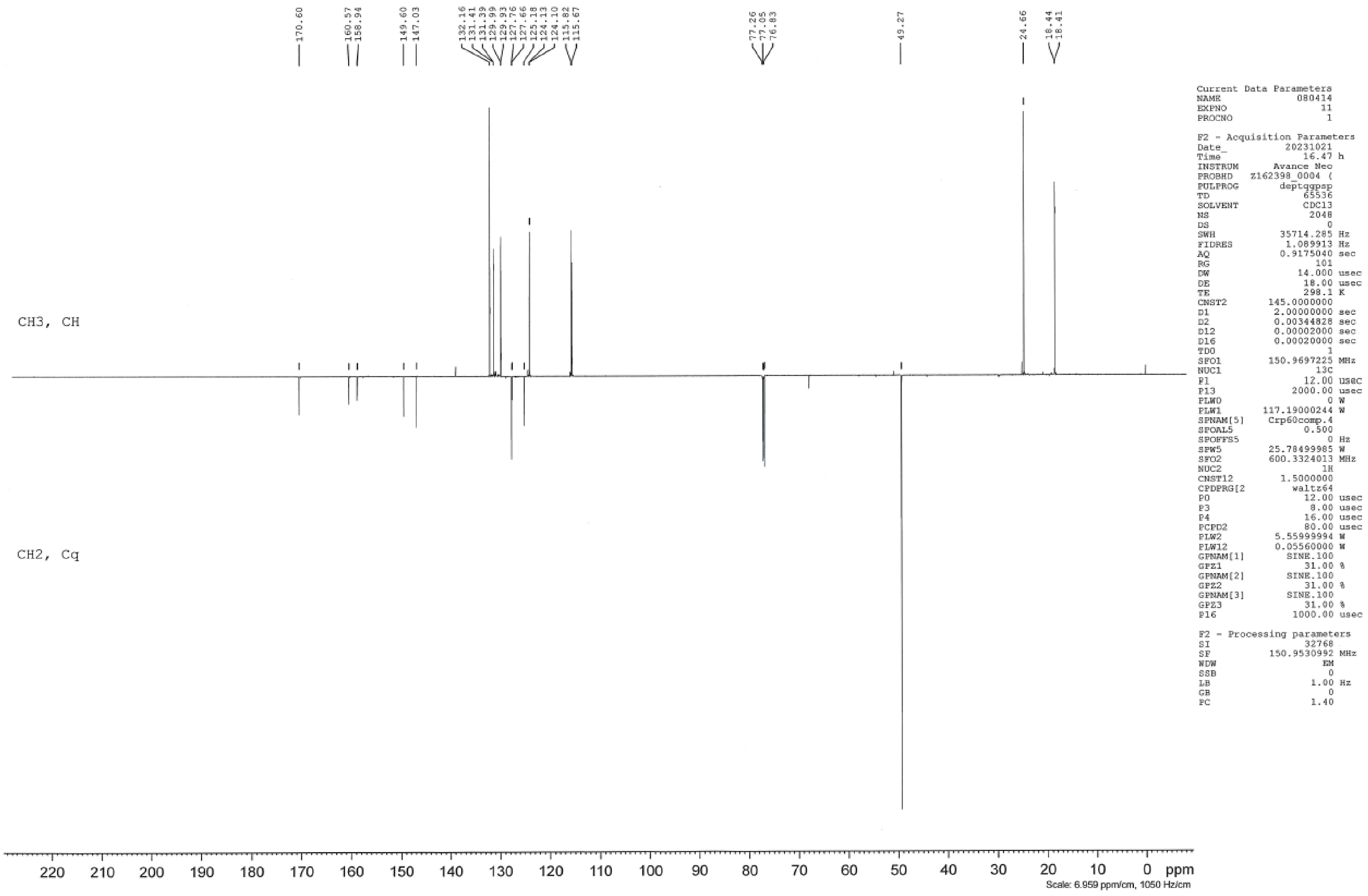
170.60
160.57
158.94
149.60
147.03
132.16
131.41
131.39
129.99
129.93
127.76
127.66
125.18
124.13
124.10
115.82
115.67
77.26
77.05
76.83
49.27
24.66
18.44
18.41
CH3, CH
CH2, Cq
Current Data Parameters
NAME 080414
EXPNO 11
PROCNO 1
F2 - Acquisition Parameters
Date_ 20231021
Time 16.47 h
INSTRUM Avance Neo
PROBHD Z162398_0004 (
PULPROG deptqgpsp
TD 65536
SOLVENT CDCl3
NS 2048
DS 0
SWH 35714.285 Hz
FIDRES 1.089913 Hz
AQ 0.9175040 sec
RG 101
DW 14.000 usec
DE 18.00 usec
TE 298.1 K
CNST2 145.0000000
D1 2.00000000 sec
D2 0.00344828 sec
D12 0.00002000 sec
D16 0.00020000 sec
TD0 1
SFO1 150.9697225 MHz
NUC1 13C
P1 12.00 usec
P13 2000.00 usec
PLW0 0 W
PLW1 117.19000244 W
SPNAM[5] Crp60comp.4
SPOAL5 0.500
SPOFFS5 0 Hz
SPW5 25.78499985 W
SFO2 600.3324013 MHz
NUC2 1H
CNST12 1.5000000
CPDPRG[2 waltz64
P0 12.00 usec
P3 8.00 usec
P4 16.00 usec
PCPD2 80.00 usec
PLW2 5.55999994 W
PLW12 0.05560000 W
GPNAM[1] SINE.100
GPZ1 31.00 %
GPNAM[2] SINE.100
GPZ2 31.00 %
GPNAM[3] SINE.100
GPZ3 31.00 %
P16 1000.00 usec
F2 - Processing parameters
SI 32768
SF 150.9530992 MHz
WDW EM
SSB 0
LB 1.00 Hz
GB 0
PC 1.40
220 210 200 190 180 170 160 150 140 130 120 110 100 90 80 70 60 50 40 30 20 10 0 ppm
Scale: 6.959 ppm/cm, 1050 Hz/cm

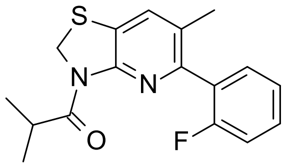

16d

1-[5-(2-Fluorophenyl)-6-methyl[1,3]thiazolo[4,5-*b*]pyridin-3(2*H*)-yl]-2-methylpropan-1-one (**16d**, 109 mg, 75%). ^{1}H NMR (600 MHz, $CDCl_3$): δ 1.15 (d, *J* = 6.7 Hz, 6H), 2.15 (d, *J* = 1.9 Hz, 3H), 4.08–4.11 (m, 1H), 5.32 (s, 2H), 7.13–7.16 (m, 1H), 7.22–7.25 (m, 1H), 7.34 (s, 1H), 7.36–7.41 (m, 2H) ppm; ^{13}C NMR ($CDCl_3$, 151 MHz): δ 18.40 (CH_3), 18.43 (CH_3), 19.1 (2 × CH_3), 32.5 (CH), 49.7 (CH_2), 115.6 (CH), 115.8 (CH), 124.08 (CH), 124.10 (CH), 125.3 (C), 127.66 (C), 127.7 (C), 127.8 (C), 129.86 (CH), 129.9 (CH), 131.38 (CH), 131.40 (CH), 132.2 (CH), 147.1 (CH), 149.4 (CH), 159.0 (C), 160.6 (C), 177.9 (C) ppm; HRMS (ESI, m/z): calcd. for $C_{17}H_{18}FN_2OS$, 317.1124 $[M+H]^+$; found 317.1109.

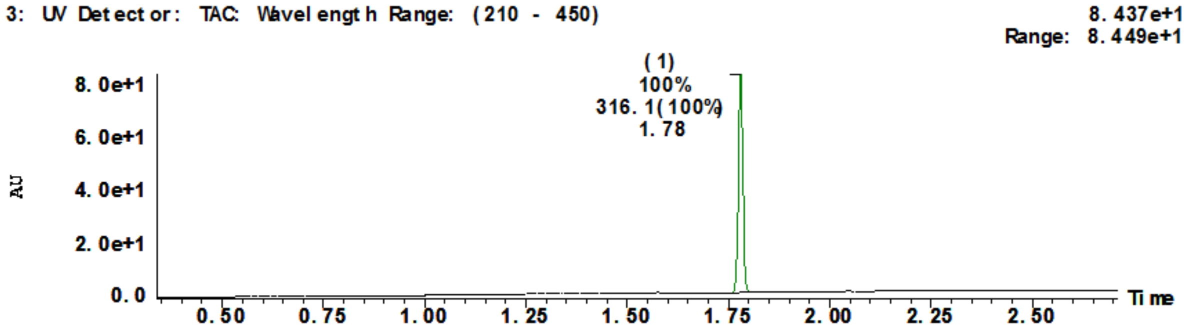

Peak Number	Time	Mass Found	Area %Total	RT LogP
1	1.78	316.10	100.00	4.37

Peak ID	Time	Mass Found
1	1.78	317

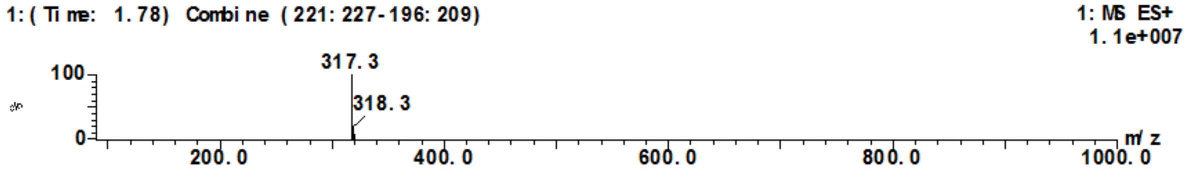

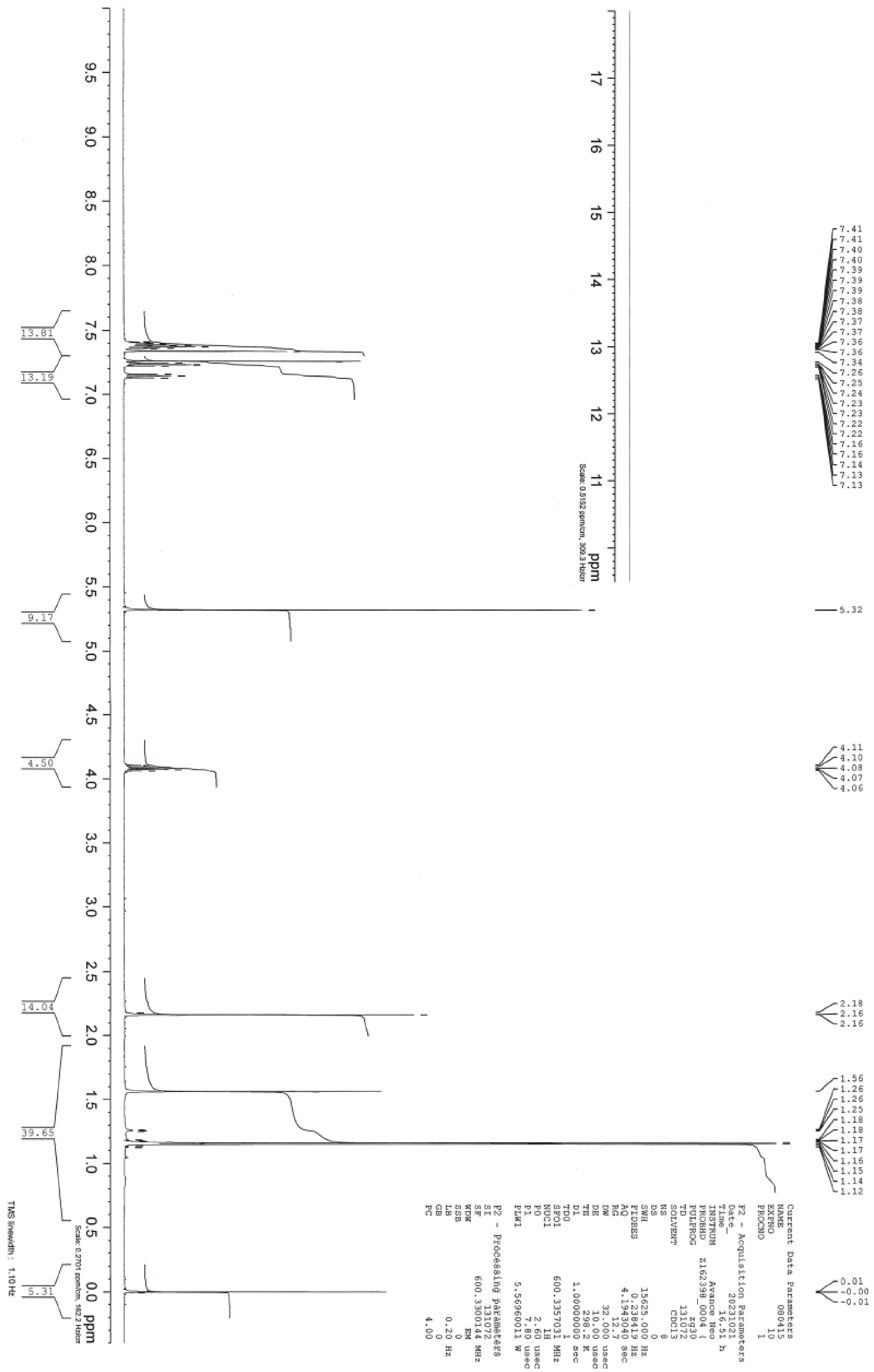

Current Data Parameters
NAME 080415
EXPNO 10
PROCNO 1
F2 - Acquisition Parameters
Date_ 20231021
Time 16.51 h
INSTRUM Avance Neo
PROBHD Z162398_0004 (
PULPROG zg30
TD 131072
SOLVENT CDCl3
NS 8
DS 0
SWH 15625.000 Hz
FIDRES 0.238419 Hz
AQ 4.1943040 sec
RG 12.7
DW 32.000 usec
DE 10.00 usec
TE 298.2 K
D1 1.00000000 sec
TD0 1
SFO1 600.3357031 MHz
NUC1 1H
P0 2.60 usec
P1 7.80 usec
PLW1 5.56960011 W
F2 - Processing parameters
SI 131072
SF 600.3300144 MHz
WDW EM
SSB 0
LB 0.20 Hz
GB 0
PC 4.00
7.41 7.41 7.40 7.40 7.39 7.39 7.39 7.38 7.38 7.37 7.37 7.36 7.36 7.34 7.26 7.25 7.24 7.23 7.23 7.22 7.22 7.16 7.16 7.14 7.13 7.13
5.32
4.11 4.10 4.08 4.07 4.06
2.18 2.16 2.16
1.56 1.26 1.26 1.25 1.18 1.18 1.17 1.17 1.16 1.15 1.14 1.12
0.01 -0.00 -0.01
13.81
13.19
9.17
4.50
14.04
39.65
5.31
ppm
Scale: 0.5152 ppm/cm, 309.3 Hz/cm
Scale: 0.2701 ppm/cm, 162.2 Hz/cm
TMS linewidth : 1.10 Hz

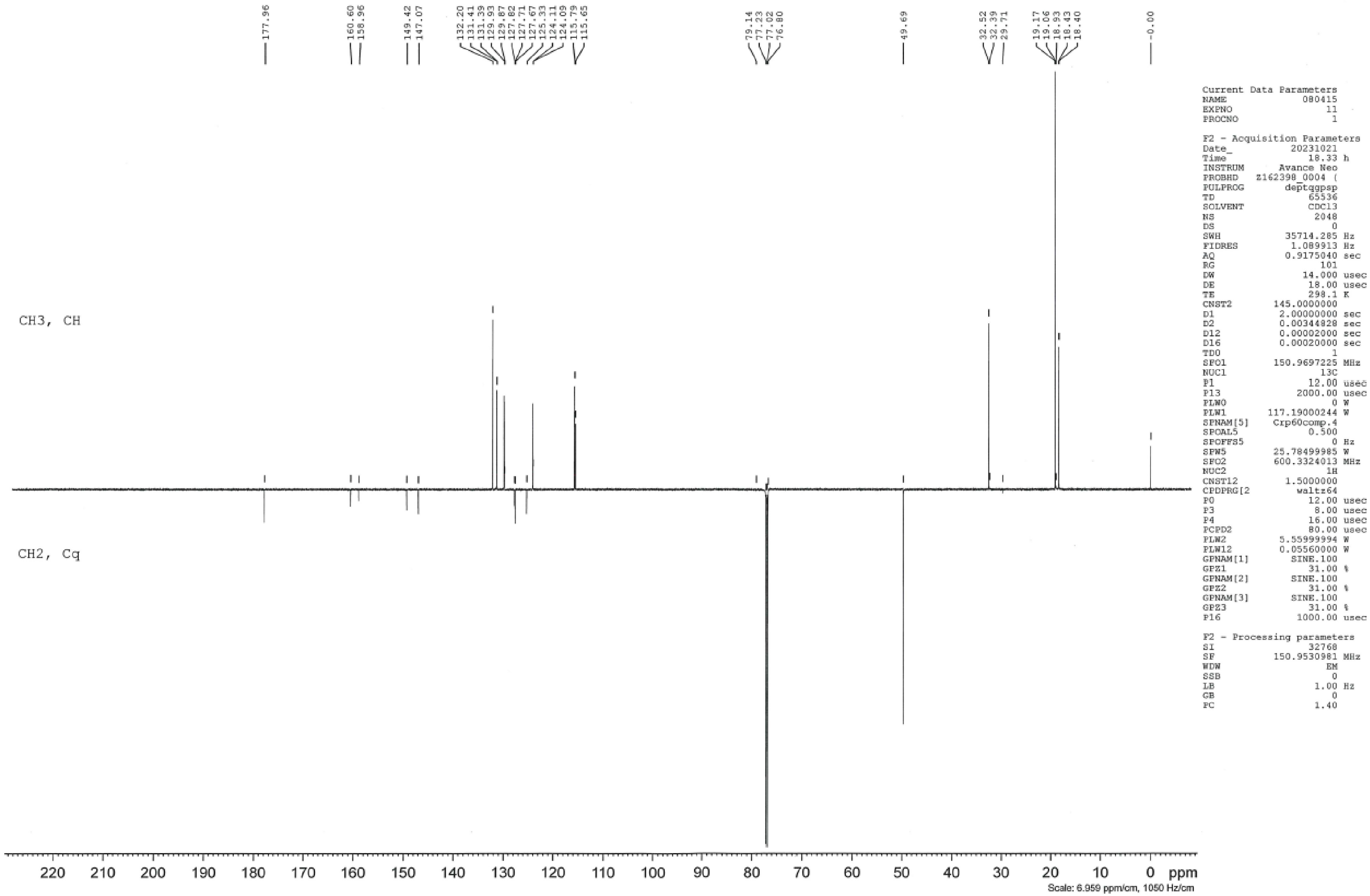
177.96
160.60
158.96
149.42
147.07
132.20
131.41
131.39
129.93
129.87
127.82
127.71
127.67
125.33
124.11
124.09
115.79
115.65
79.14
77.23
77.02
76.80
49.69
32.52
32.39
29.71
19.17
19.06
18.93
18.43
18.40
-0.00
CH3, CH
CH2, Cq
220 210 200 190 180 170 160 150 140 130 120 110 100 90 80 70 60 50 40 30 20 10 0 ppm
Scale: 6.959 ppm/cm, 1050 Hz/cm
Current Data Parameters
NAME 080415
EXPNO 11
PROCNO 1
F2 - Acquisition Parameters
Date_ 20231021
Time 18.33 h
INSTRUM Avance Neo
PROBHD Z162398_0004 (
PULPROG deptqgpsp
TD 65536
SOLVENT CDCl3
NS 2048
DS 0
SWH 35714.285 Hz
FIDRES 1.089913 Hz
AQ 0.9175040 sec
RG 101
DW 14.000 usec
DE 18.00 usec
TE 298.1 K
CNST2 145.0000000
D1 2.00000000 sec
D2 0.00344828 sec
D12 0.00002000 sec
D16 0.00020000 sec
TD0 1
SFO1 150.9697225 MHz
NUC1 13C
P1 12.00 usec
P13 2000.00 usec
PLW0 0 W
PLW1 117.19000244 W
SPNAM[5] Crp60comp.4
SPOAL5 0.500
SPOFFS5 0 Hz
SPW5 25.78499985 W
SFO2 600.3324013 MHz
NUC2 1H
CNST12 1.5000000
CPDPRG[2 waltz64
P0 12.00 usec
P3 8.00 usec
P4 16.00 usec
PCPD2 80.00 usec
PLW2 5.55999994 W
PLW12 0.05560000 W
GPNAM[1] SINE.100
GPZ1 31.00 %
GPNAM[2] SINE.100
GPZ2 31.00 %
GPNAM[3] SINE.100
GPZ3 31.00 %
P16 1000.00 usec
F2 - Processing parameters
SI 32768
SF 150.9530981 MHz
WDW EM
SSB 0
LB 1.00 Hz
GB 0
PC 1.40

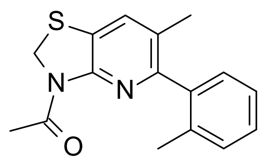

16e

1-[6-Methyl-5-(2-methylphenyl)[1,3]thiazolo[4,5-*b*]pyridin-3(2*H*)-yl]ethanone (**16e**, 150 mg, 84%). ^{1}H NMR (600 MHz, $CDCl_3$): δ 2.02 (s, 3H), 2.14 (s, 3H), 2.57 (s, 3H), 5.33 (s, 2H), 7.13 (d, *J* = 7.2 Hz, 1H), 7.22–7.31 (m, 3H), 7.34 (s, 1H) ppm; ^{13}C NMR ($CDCl_3$, 151 MHz): δ 18.5 (CH_3), 19.6 (CH_3), 24.6 (CH_3), 49.2 (CH_2), 124.2 (C), 125.6 (CH), 126.7 (C), 127.9 (CH), 129.0 (CH), 130.2 (CH), 132.2 (CH), 135.9 (C), 139.5 (C), 149.3 (C), 152.8 (C), 170.6 (C) ppm; HRMS (ESI, m/z): calcd. for $C_{16}H_{17}N_2OS$, 285.1062 $[M+H]^+$; found 285.1066.

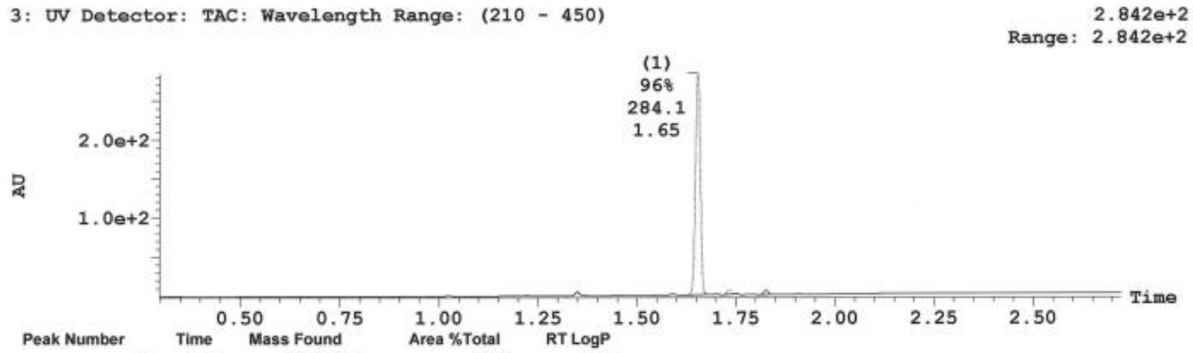

Peak Number	Time	Mass Found	Area %Total	RT LogP
1	1.65	284.0983	96.30	3.75
2	1.73	284.0983	1.88	4.15
3	1.83	Not Found	1.82	4.68

Peak ID	Time	Mass Found
1	1.65	285

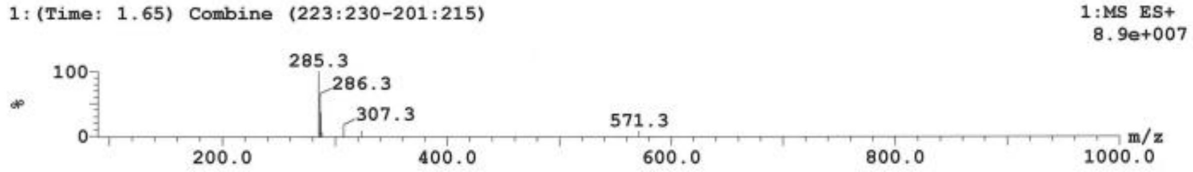

Peak ID	Time	Mass Found
1	1.65	Not Found

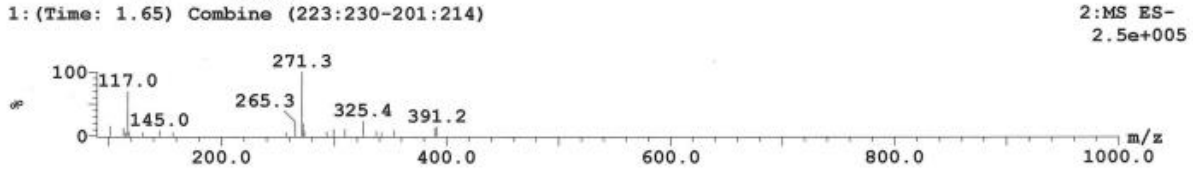

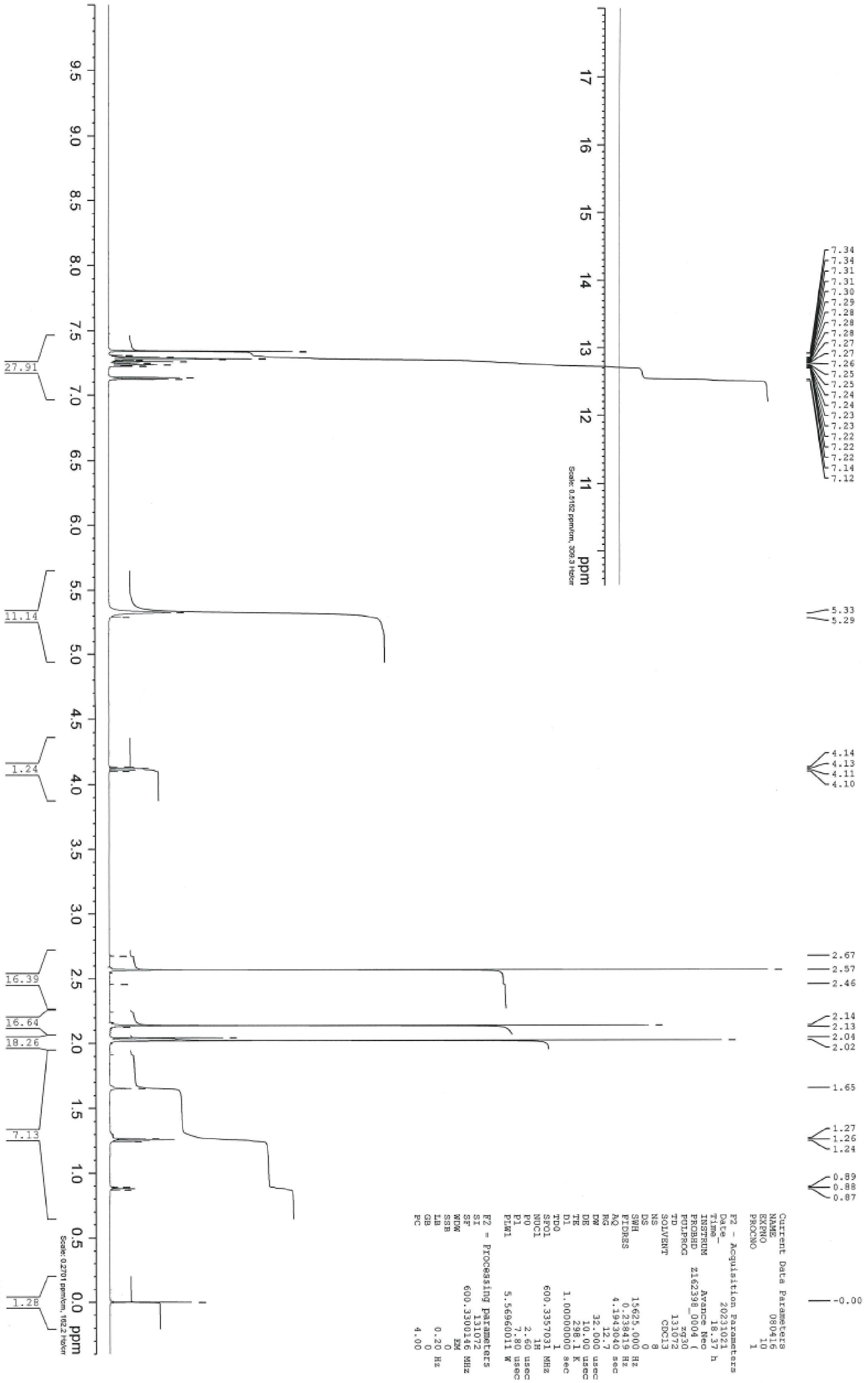
Current Data Parameters
NAME 080416
EXPNO 10
PROCNO 1
F2 - Acquisition Parameters
Date_ 20231021
Time 18.37 h
INSTRUM Avance Neo
PROBHD Z162398_0004 (
PULPROG zg30
TD 131072
SOLVENT CDCl3
NS 8
DS 0
SWH 15625.000 Hz
FIDRES 0.238419 Hz
AQ 4.1943040 sec
RG 12.7
DW 32.000 usec
DE 10.00 usec
TE 298.1 K
D1 1.00000000 sec
TD0 1
SFO1 600.3357031 MHz
NUC1 1H
P0 2.60 usec
P1 7.80 usec
PLW1 5.56960011 W
F2 - Processing parameters
SI 131072
SF 600.3300146 MHz
WDW EM
SSB 0
LB 0.20 Hz
GB 0
PC 4.00
TMS linewidth: 0.82 Hz
ppm

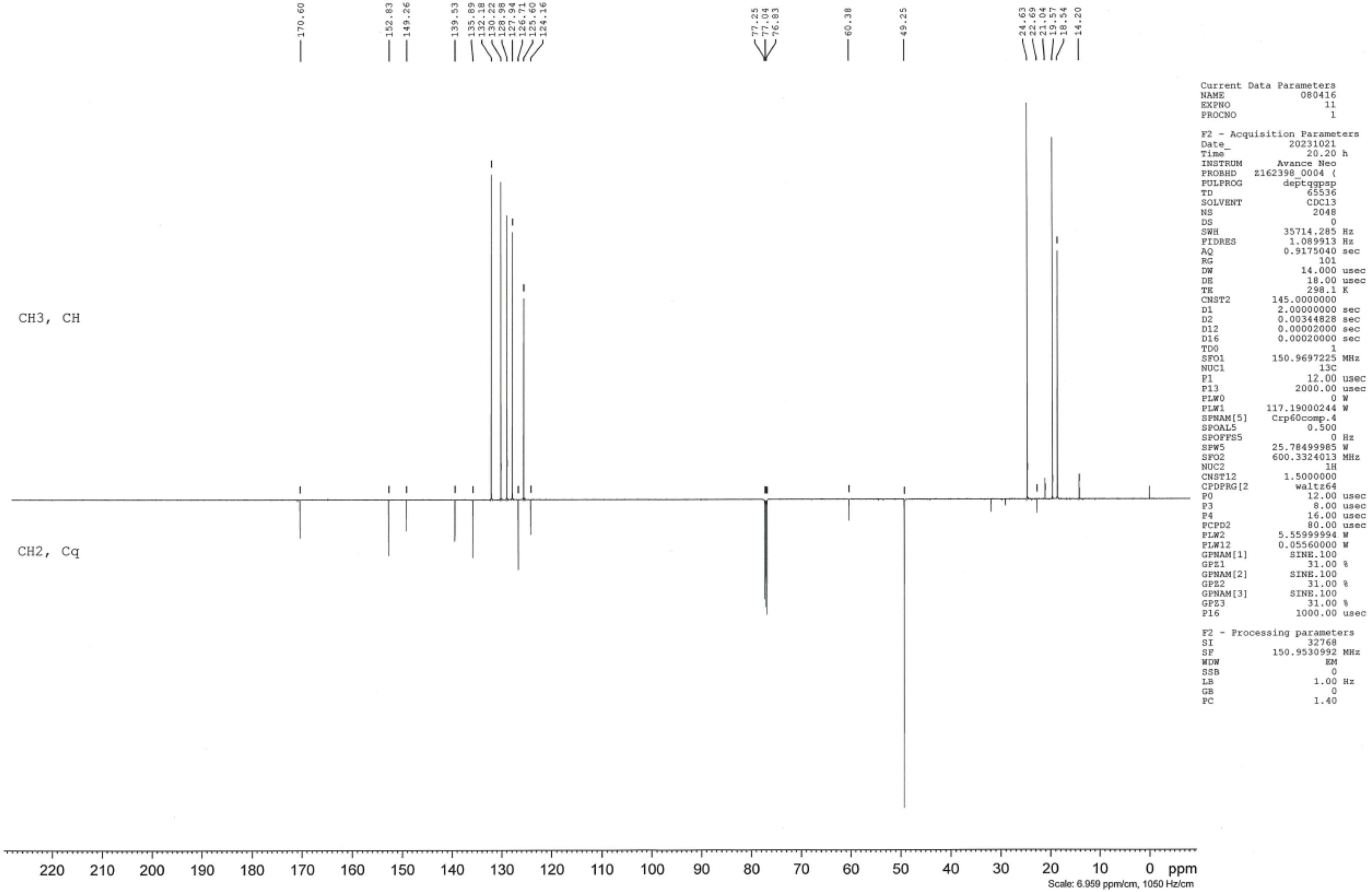
170.60
152.83
149.26
139.53
135.89
132.18
130.22
128.98
127.94
126.71
125.60
124.16
77.25
77.04
76.83
60.38
49.25
24.63
22.69
21.04
19.57
18.54
14.20
CH3, CH
CH2, Cq
220 210 200 190 180 170 160 150 140 130 120 110 100 90 80 70 60 50 40 30 20 10 0 ppm
Scale: 6.959 ppm/cm, 1050 Hz/cm
Current Data Parameters
NAME 080416
EXPNO 11
PROCNO 1
F2 - Acquisition Parameters
Date_ 20231021
Time 20.20 h
INSTRUM Avance Neo
PROBHD Z162398_0004 (
PULPROG deptqgpsp
TD 65536
SOLVENT CDCl3
NS 2048
DS 0
SWH 35714.285 Hz
FIDRES 1.089913 Hz
AQ 0.9175040 sec
RG 101
DW 14.000 usec
DE 18.00 usec
TE 298.1 K
CNST2 145.0000000
D1 2.00000000 sec
D2 0.00344828 sec
D12 0.00002000 sec
D16 0.00020000 sec
TD0 1
SFO1 150.9697225 MHz
NUC1 13C
P1 12.00 usec
P13 2000.00 usec
PLW0 0 W
PLW1 117.19000244 W
SPNAM[5] Crp60comp.4
SPOAL5 0.500
SPOFFS5 0 Hz
SPW5 25.78499985 W
SFO2 600.3324013 MHz
NUC2 1H
CNST12 1.5000000
CPDPRG[2 waltz64
P0 12.00 usec
P3 8.00 usec
P4 16.00 usec
PCPD2 80.00 usec
PLW2 5.55999994 W
PLW12 0.05560000 W
GPNAM[1] SINE.100
GPZ1 31.00 %
GPNAM[2] SINE.100
GPZ2 31.00 %
GPNAM[3] SINE.100
GPZ3 31.00 %
P16 1000.00 usec
F2 - Processing parameters
SI 32768
SF 150.9530992 MHz
WDW EM
SSB 0
LB 1.00 Hz
GB 0
PC 1.40

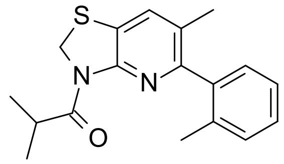

16f

2-Methyl-1-[6-methyl-5-(2-methylphenyl)[1,3]thiazolo[4,5-*b*]pyridin-3(2*H*)-yl]propan-1-one (**16f**, 145 mg, 74%). ^{1}H NMR (600 MHz, CDCl$_3$): δ 1.10 (d, *J* = 6.7 Hz, 6H), 2.01 (s, 3H), 2.13 (s, 3H), 5.33 (s, 2H), 4.08–4.12 (m, 1H),5.33 (s, 2H), 7.12 (d, *J* = 7.2 Hz, 1H), 7.23–7.31 (m, 3H), 7.34 (s, 1H) ppm; ^{13}C NMR (CDCl$_3$, 151 MHz): δ 18.5 (2 × CH$_3$), 19.5 (CH$_3$), 32.3 (CH$_3$), 49.7 (CH$_2$), 124.4 (C), 125.6 (CH), 126.6 (C), 127.9 (CH), 129.0 (CH), 130.1 (CH), 132.2 (CH), 135.8 (C), 139.6 (C), 149.1 (C), 152.9 (C), 178.0 (C) ppm; HRMS (ESI, m/z): calcd. for $C_{18}H_{21}N_2OS$, 313.1375 [M+H]$^+$; found 313.1363.

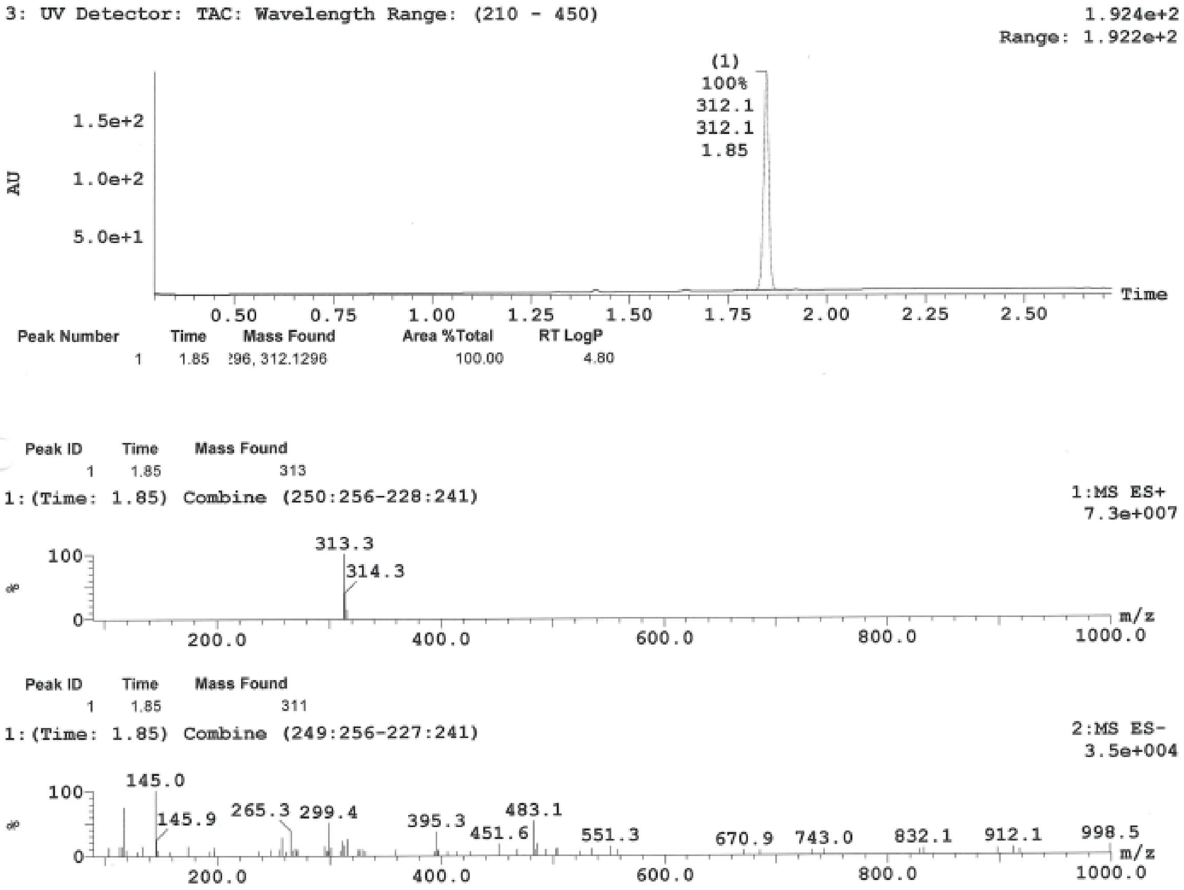

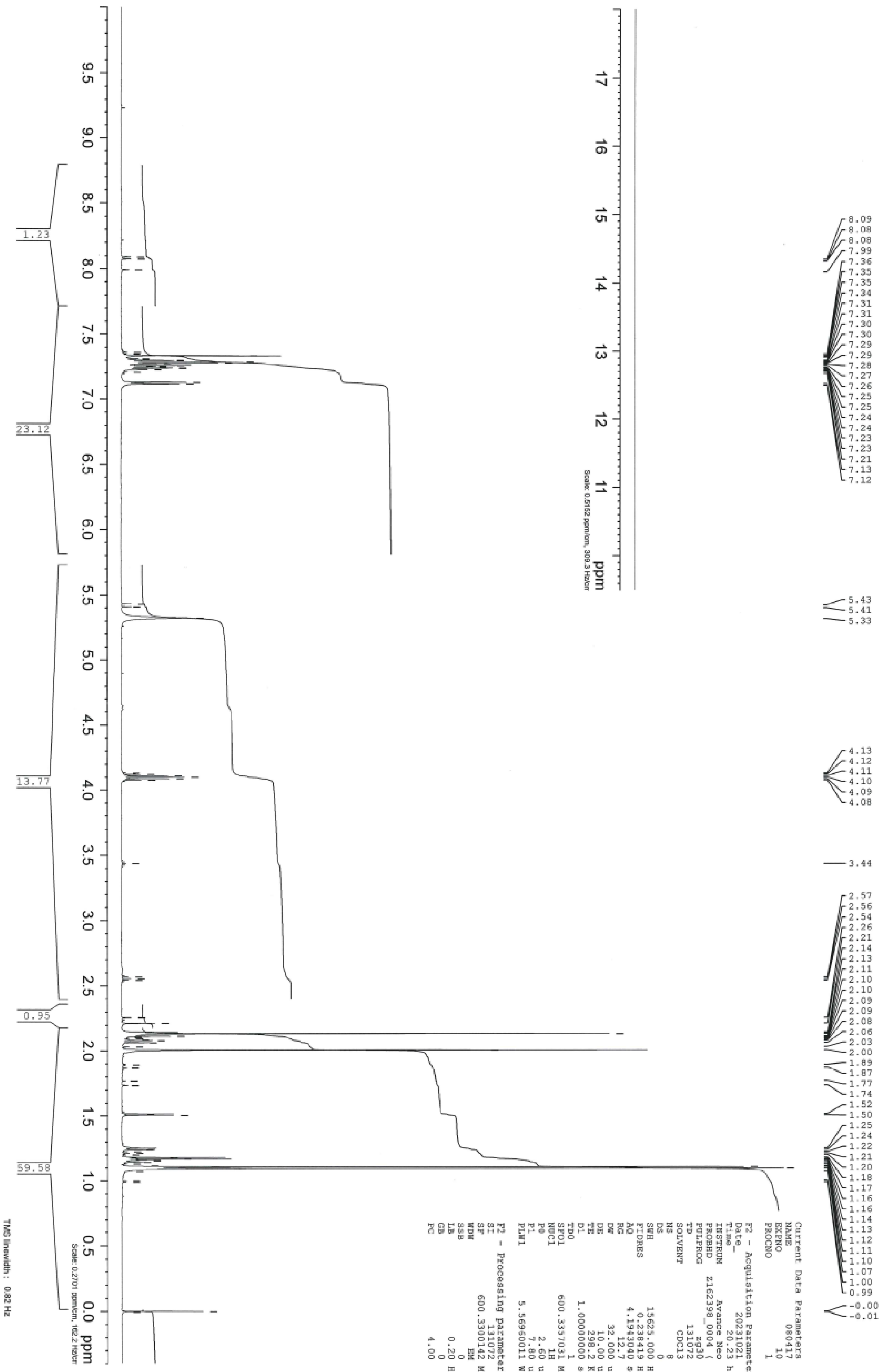
Current Data Parameters
NAME 080417
EXPNO 10
PROCNO 1
F2 - Acquisition Parameters
Date_ 20231021
Time 20.23 h
INSTRUM Avance Neo
PROBHD Z162398_0004 (
PULPROG zg30
TD 131072
SOLVENT CDCl3
NS 8
DS 0
SWH 15625.000 Hz
FIDRES 0.238419 Hz
AQ 4.1943040 sec
RG 12.7
DW 32.000 usec
DE 10.00 usec
TE 298.2 K
D1 1.00000000 sec
TD0 1
SFO1 600.3357031 MHz
NUC1 1H
P0 2.60 usec
P1 7.80 usec
PLW1 5.56960011 W
F2 - Processing parameters
SI 131072
SF 600.3300142 MHz
WDW EM
SSB 0
LB 0.20 Hz
GB 0
PC 4.00
1.23
23.12
13.77
0.95
59.58
ppm
TMS linewidth : 0.82 Hz

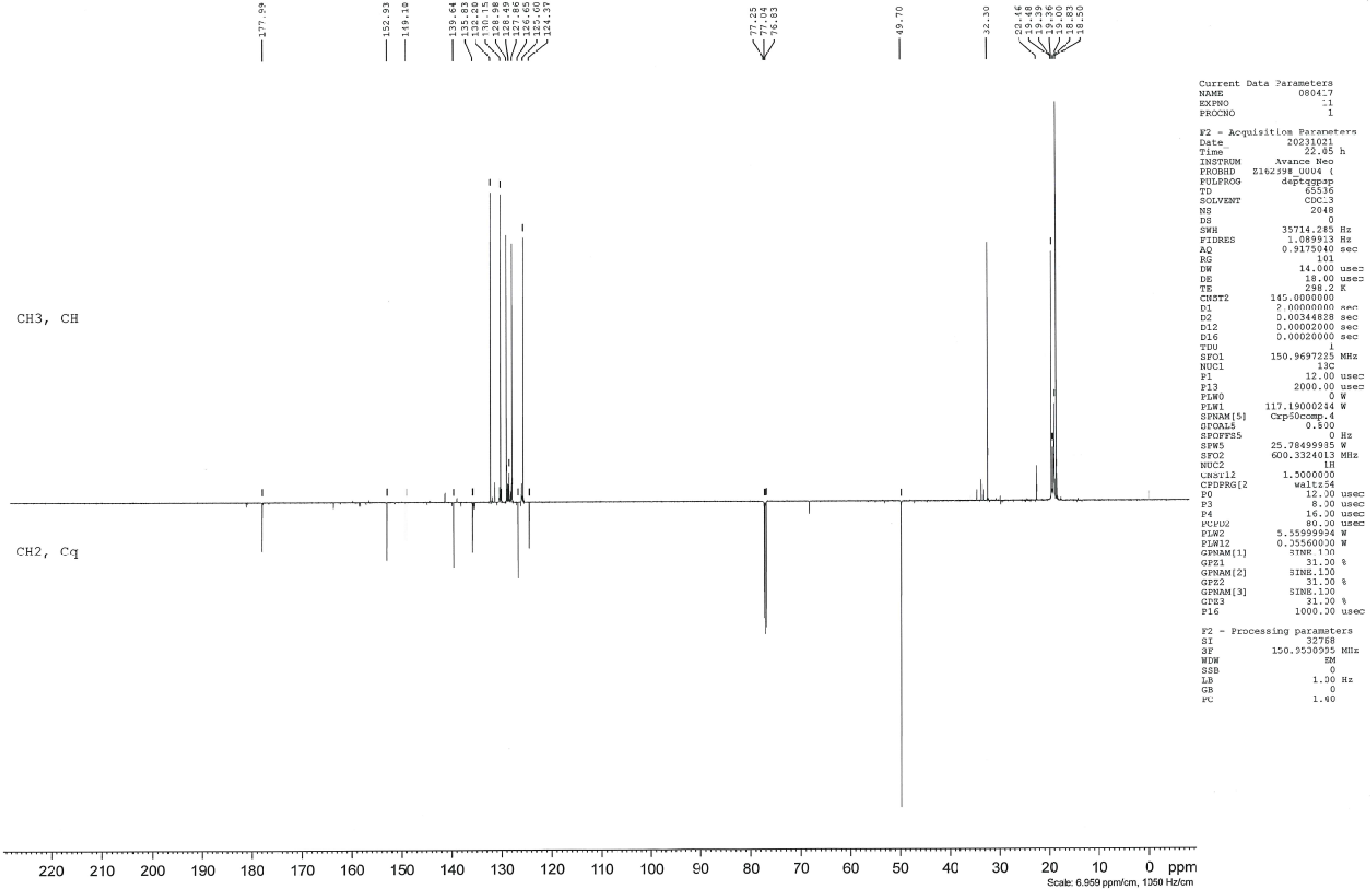

177.99
152.93
149.10
139.64
135.83
132.20
130.15
128.98
128.49
127.86
126.65
125.60
124.37
77.25
77.04
76.83
49.70
32.30
22.46
19.48
19.39
19.36
19.00
18.83
18.50
CH3, CH
CH2, Cq
Current Data Parameters
NAME 080417
EXPNO 11
PROCNO 1
F2 - Acquisition Parameters
Date_ 20231021
Time 22.05 h
INSTRUM Avance Neo
PROBHD Z162398_0004 (
PULPROG deptqgpsp
TD 65536
SOLVENT CDCl3
NS 2048
DS 0
SWH 35714.285 Hz
FIDRES 1.089913 Hz
AQ 0.9175040 sec
RG 101
DW 14.000 usec
DE 18.00 usec
TE 298.2 K
CNST2 145.0000000
D1 2.00000000 sec
D2 0.00344828 sec
D12 0.00002000 sec
D16 0.00020000 sec
TD0 1
SFO1 150.9697225 MHz
NUC1 13C
P1 12.00 usec
P13 2000.00 usec
PLW0 0 W
PLW1 117.19000244 W
SPNAM[5] Crp60comp.4
SPOAL5 0.500
SPOFFS5 0 Hz
SPW5 25.78499985 W
SFO2 600.3324013 MHz
NUC2 1H
CNST12 1.5000000
CPDPRG[2 waltz64
P0 12.00 usec
P3 8.00 usec
P4 16.00 usec
PCPD2 80.00 usec
PLW2 5.55999994 W
PLW12 0.05560000 W
GPNAM[1] SINE.100
GPZ1 31.00 %
GPNAM[2] SINE.100
GPZ2 31.00 %
GPNAM[3] SINE.100
GPZ3 31.00 %
P16 1000.00 usec
F2 - Processing parameters
SI 32768
SF 150.9530995 MHz
WDW EM
SSB 0
LB 1.00 Hz
GB 0
PC 1.40
220 210 200 190 180 170 160 150 140 130 120 110 100 90 80 70 60 50 40 30 20 10 0 ppm
Scale: 6.959 ppm/cm, 1050 Hz/cm

1.2.9. Characterization of borylated compounds 17a–e and side product 17f

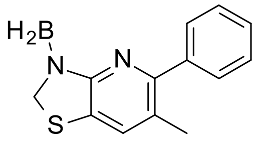

17a

In a dedicated additional synthesis complementing the optimization work outlined in Table 1 of the Main Manuscript, 6-methyl-5-phenyl[1,3]thiazolo[4,5-*b*]pyridine (**15a**, 3.07 mmol, 1.0 equiv) was dissolved in absolute toluene (10 mL) in a flame-dried round-bottom flask under argon. Ammonia borane (285 mg, 9.20 mmol, 3.0 equiv) and $B(C_6F_5)_3$ (79 mg, 0.15 mmol, 0.05 equiv) were added to the solution and the resulting reaction mixture was stirred at a temperature of 45 °C for 5 h and concentrated thereafter under reduced pressure. The remaining crude product was purified carefully via column chromatography (gradient ethyl acetate/hexane) to afford the borylated 2,3-dihydro[1,3]thiazolo[4,5-*b*]pyridine **17a**.

3-Boryl-6-methyl-5-phenyl-2,3-dihydro[1,3]thiazolo[4,5-*b*]pyridine (**17a**, 49 mg, 11%). ^{1}H NMR (400 MHz, $CDCl_3$): δ 1.63 (br s, 2H), 2.15 (s, 3H), 4.69-4.72 (m, 2H), 7.10-7.12 (m, 1H), 7.31-7.38 (m, 2H),7.39–7.48 (m, 3H), 7.21–7.24 (m, 1H) ppm; HRMS (ESI, m/z): calcd. for $C_{14}H_{15}BN_2S$ $[M]^+$ 254.1049; found 254.1042.

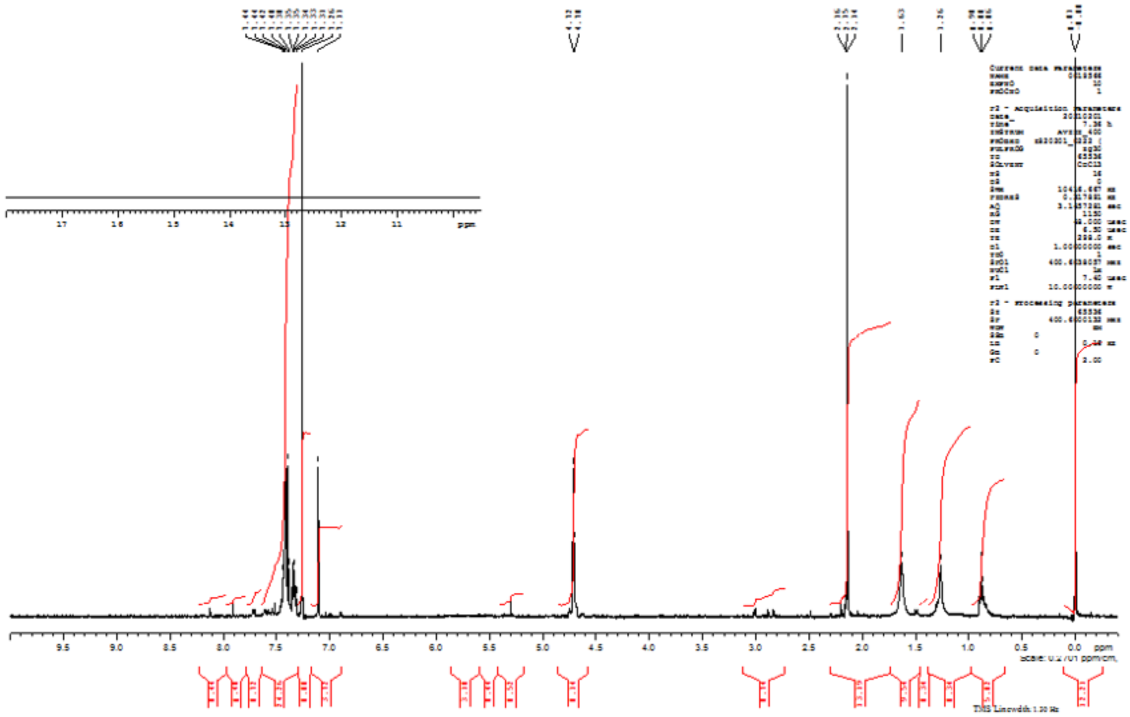

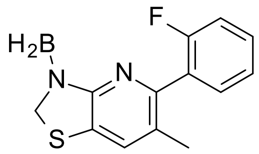

17b

In a dedicated additional synthesis complementing the optimization work outlined in Table 1 of the Main Manuscript, 6-methyl-5-(2-fluorophenyl)-[1,3]thiazolo[4,5-*b*]pyridine (**5**, 3.07 mmol, 1.0 equiv) was dissolved in absolute toluene (10 mL) in a flame-dried round-bottom flask under argon. Ammonia borane (285 mg, 9.20 mmol, 3.0 equiv) and $B(C_6F_5)_3$ (79 mg, 0.15 mmol, 0.05 equiv) were added to the solution and the resulting reaction mixture was stirred at a temperature of 45 °C for 5 h and concentrated thereafter under reduced pressure. The remaining crude product was purified carefully via column chromatography (gradient ethyl acetate/hexane) to afford the borylated 2,3-dihydro[1,3]thiazolo[4,5-*b*]pyridine **17b** together with inseparable impurities that may have emerged from further azaboretidine structures.

3-Boryl-6-methyl-5-(2-fluorophenyl)-2,3-dihydro[1,3]thiazolo[4,5-*b*]pyridine (**17b**, 78 mg, 83% purity, 18%). ^{1}H NMR (400 MHz, $CDCl_3$): δ 1.77 (s, 3H), 2.40-2.65 (br. d, 2H), 5.05-5.12 (m, 2H), 6.89-6.91 (m, 1H), 7.08-7.25 (m, 3H), 7.32–7.48 (m, 1H) ppm; HRMS (ESI, m/z): calcd. for $C_{13}H_{12}BFN_2S$ $[M]^+$ 258.0798; found 258.0805.

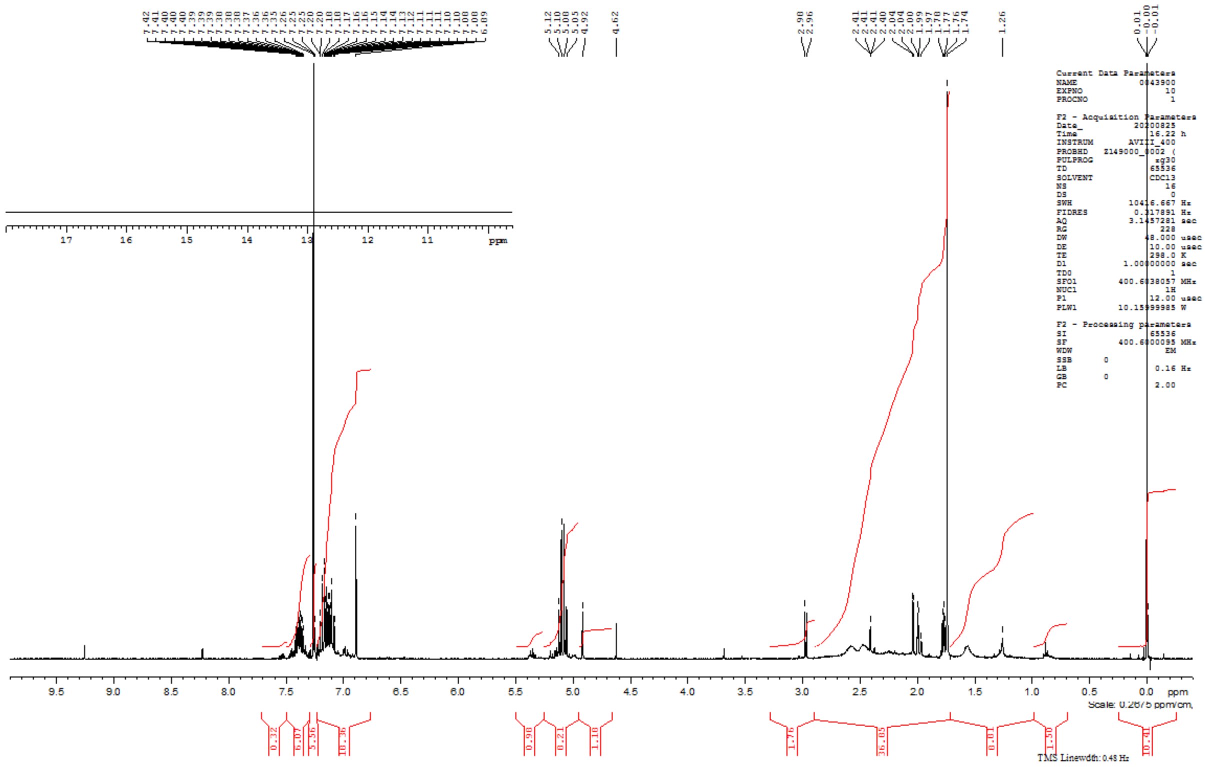

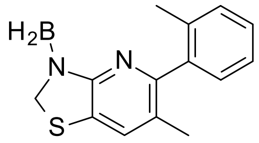

17c

In a dedicated additional synthesis complementing the optimization work outlined in Table 1 of the Main Manuscript, 6-methyl-5-(2-methylphenyl)[1,3]thiazolo[4,5-*b*]pyridine (**15c**, 3.07 mmol, 1.0 equiv) was dissolved in absolute toluene (10 mL) in a flame-dried round-bottom flask under argon. Ammonia borane (285 mg, 9.20 mmol, 3.0 equiv) and $B(C_6F_5)_3$ (79 mg, 0.15 mmol, 0.05 equiv) were added to the solution and the resulting reaction mixture was stirred at a temperature of 45 °C for 5 h and concentrated thereafter under reduced pressure. The remaining crude product was purified carefully via column chromatography (gradient ethyl acetate/hexane) to afford the borylated 2,3-dihydro[1,3]thiazolo[4,5-*b*]pyridine **17c**.

3-Boryl-6-methyl-5-(2-methylphenyl)-2,3-dihydro[1,3]thiazolo[4,5-*b*]pyridine (**17c**, 61 mg, 12%). ^{1}H NMR (400 MHz, $CDCl_3$): δ 2.03 (s, 3H), 2.31 (s, 3H), 2.51 (br s, 2H), 5.11–5.15 (m, 2H), 6.87-6.89 (m, 1H), 7.10–7.18 (m, 3H), 7.21–7.24 (m, 1H) ppm; HRMS (ESI, m/z): calcd. for $C_{14}H_{15}BN_2S$ $[M]^+$ 254.1049; found 254.1042.

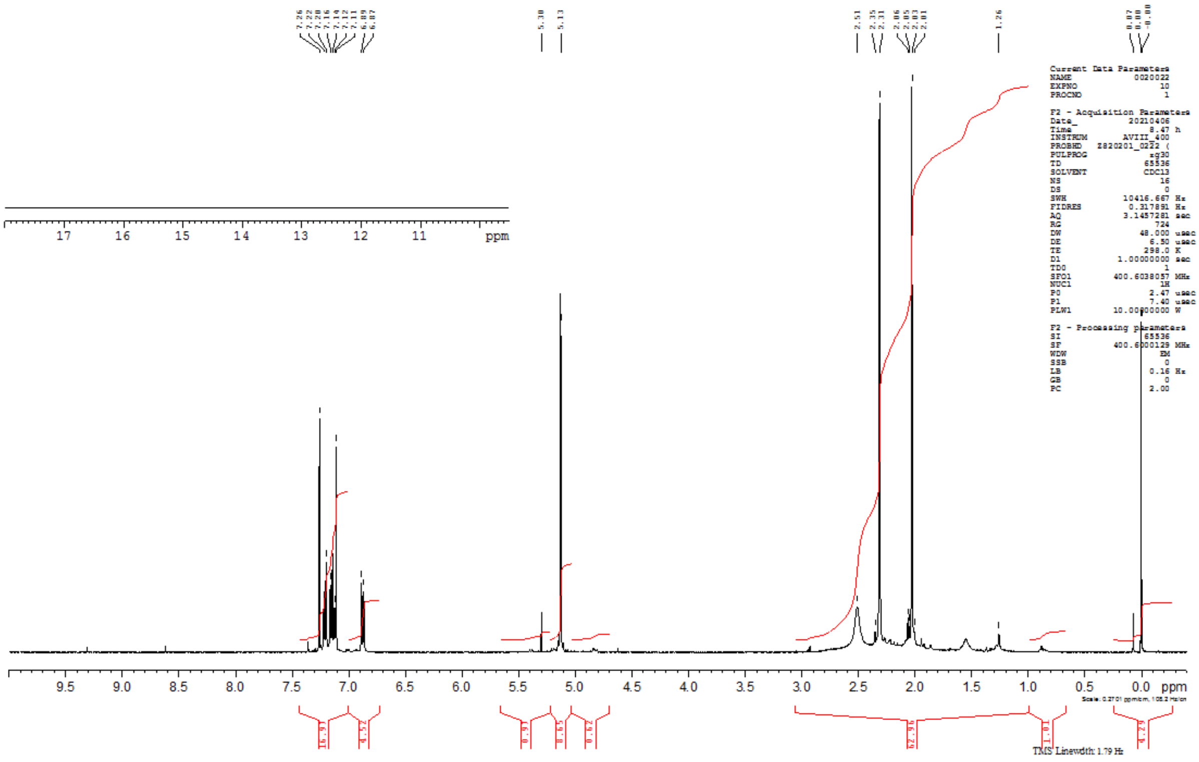

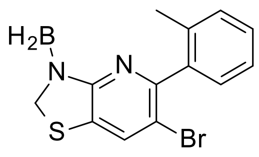

17d

6-Bromo-5-(2-methylphenyl)[1,3]thiazolo[4,5-*b*]pyridine (3.07 mmol, 1.0 equiv) was dissolved in absolute toluene (10 mL) in a flame-dried round-bottom flask under argon. Ammonia borane (285 mg, 9.20 mmol, 3.0 equiv) and $B(C_6F_5)_3$ (79 mg, 0.15 mmol, 0.05 equiv) were added to the solution and the resulting reaction mixture was stirred at a temperature of 45 °C for 5 h and concentrated thereafter under reduced pressure. The remaining residue was redissolved in acetonitrile, formic acid was added, and the reaction mixture was stirred at room temperature for 2 h. The phases were separated via phase separator and the organic layer was concentrated under reduced pressure. The remaining crude product was purified via column chromatography (gradient ethyl acetate/hexane) to afford the desired substituted 6-bromo-2,3-dihydro[1,3]thiazolo[4,5-*b*]pyridine **13c**. As this reduction was subject to a detailed optimization, borylated intermediates **17d**, **17e** and arylated side product **17f** could be isolated in several cases depending on the reaction conditions.

3-Boryl-6-bromo-5-(2-methylphenyl)-2,3-dihydro[1,3]thiazolo[4,5-*b*]pyridine (**17d**, 179 mg, 29%). ^{1}H NMR (600 MHz, $CDCl_3$): δ 2.14 (s, 3H), 2.51 (brs, 2H), 5.11–5.14 (m, 2H), 7.01–7.05 (m, 1H), 7.10–7.12 (m, 1H), 7.22–7.25 (m, 2H), 7.30–7.34 (m, 1H) ppm; ^{13}C NMR ($CDCl_3$, 151 MHz): δ 19.4 (CH_3), 56.8 (CH_2), 104.0 (C), 125.6 (CH), 128.3 (CH), 128.9 (CH), 129.0 (CH), 129.7 (CH), 131.0 (C), 136.3 (C), 136.4 (C), 144.3 (C), 157.5 (C) ppm; HRMS (ESI, m/z): calcd. for $C_{13}H_{12}BBrN_2S$ $[M]^+$ 317.9997; found 317.9982.

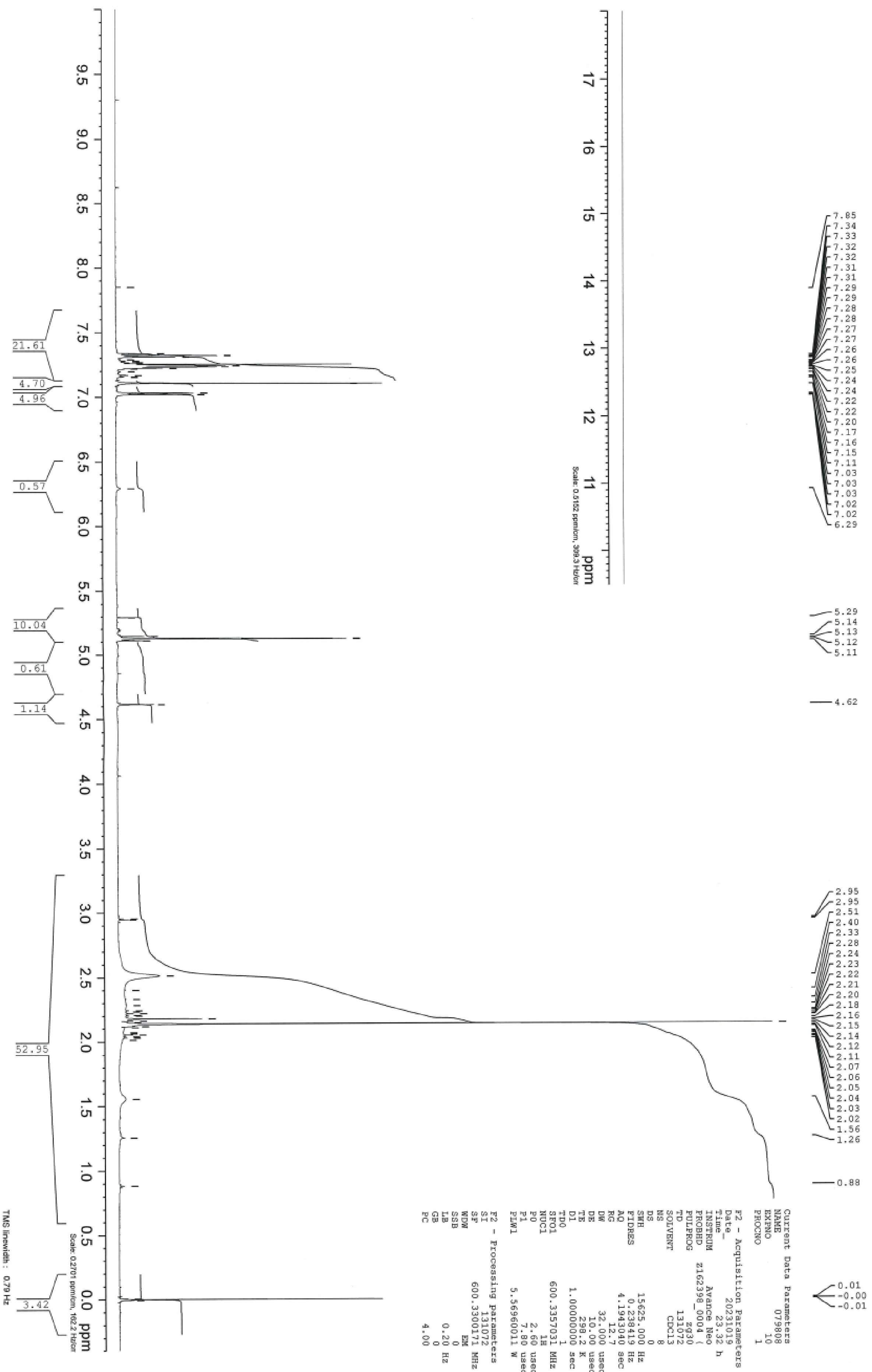

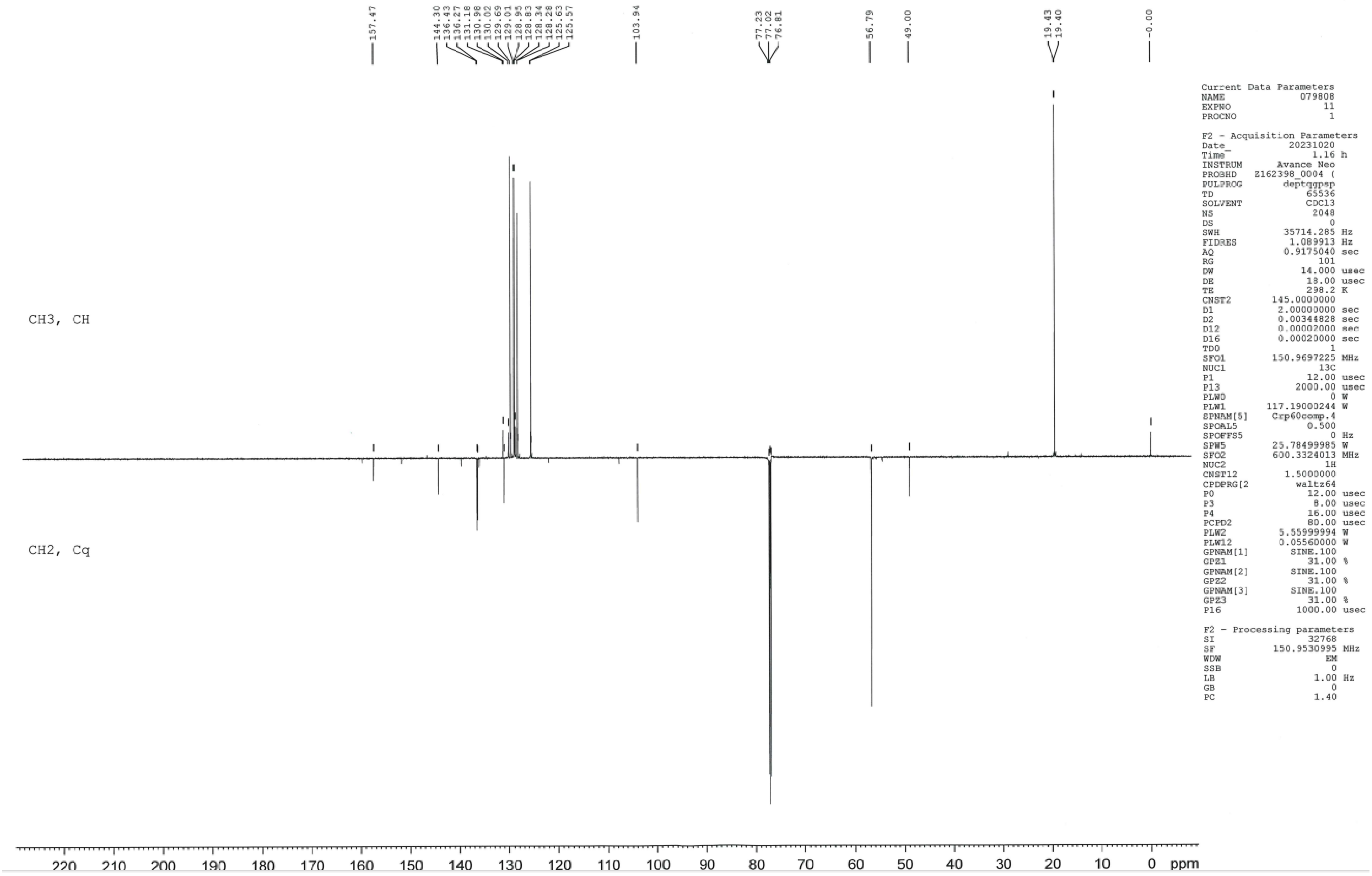
157.47
144.30
136.43
136.27
131.18
130.98
130.02
129.69
129.01
128.95
128.83
128.34
128.28
125.63
125.57
103.94
77.23
77.02
76.81
56.79
49.00
19.43
19.40
-0.00
CH3, CH
CH2, Cq
Current Data Parameters
NAME 079808
EXPNO 11
PROCNO 1
F2 - Acquisition Parameters
Date_ 20231020
Time_ 1.16 h
INSTRUM Avance Neo
PROBHD Z162398_0004 (
PULPROG deptqgpsp
TD 65536
SOLVENT CDCl3
NS 2048
DS 0
SWH 35714.285 Hz
FIDRES 1.089913 Hz
AQ 0.9175040 sec
RG 101
DW 14.000 usec
DE 18.00 usec
TE 298.2 K
CNST2 145.0000000
D1 2.00000000 sec
D2 0.00344828 sec
D12 0.00002000 sec
D16 0.00020000 sec
TD0 1
SFO1 150.9697225 MHz
NUC1 13C
P1 12.00 usec
P13 2000.00 usec
PLW0 0 W
PLW1 117.19000244 W
SPNAM[5] Crp60comp.4
SPOAL5 0.500
SPOFFS5 0 Hz
SPW5 25.78499985 W
SFO2 600.3324013 MHz
NUC2 1H
CNST12 1.5000000
CPDPRG[2 waltz64
P0 12.00 usec
P3 8.00 usec
P4 16.00 usec
PCPD2 80.00 usec
PLW2 5.55999994 W
PLW12 0.05560000 W
GPNAM[1] SINE.100
GPZ1 31.00 %
GPNAM[2] SINE.100
GPZ2 31.00 %
GPNAM[3] SINE.100
GPZ3 31.00 %
P16 1000.00 usec
F2 - Processing parameters
SI 32768
SF 150.9530995 MHz
WDW EM
SSB 0
LB 1.00 Hz
GB 0
PC 1.40
220 210 200 190 180 170 160 150 140 130 120 110 100 90 80 70 60 50 40 30 20 10 0 ppm

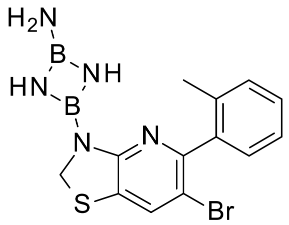

17e

4-[6-Bromo-5-(2-methylphenyl)[1,3]thiazolo[4,5-*b*]pyridin-3(2*H*)-yl]-1,3,2,4-diazadiboretidin-2-amine (**17e**, 106 mg, 9%). ^{1}H NMR (600 MHz, $CDCl_3$): δ 2.12 (s, 3H), 1.90-2.40 (br m, 2H), 2.62-2.78 (br d, 1H), 3.24-3.41 (br d, 1H), 5.17–5.19 (m, 1H), 5.31–5.35 (m, 1H), 6.99–7.03 (m, 1H), 7.22–7.28 (m, 2H), 7.31–7.36 (m, 1H), 8.16–8.17 (m, 1H) ppm; ^{13}C NMR ($CDCl_3$, 151 MHz): δ 19.4 (CH_3), 55.5 (CH_2), 104.3 (C), 125.8 (CH), 128.4 (CH), 128.9 (CH), 129.2 (CH), 129.7 (CH), 130.0 (C), 135.8 (C), 136.4 (C), 144.3 (C), 157.3 (C) ppm; HRMS (ESI, m/z): calcd. for $C_{13}H_{14}B_2BrN_5S$ $[M]^+$ 373.0339; found 373.0352.

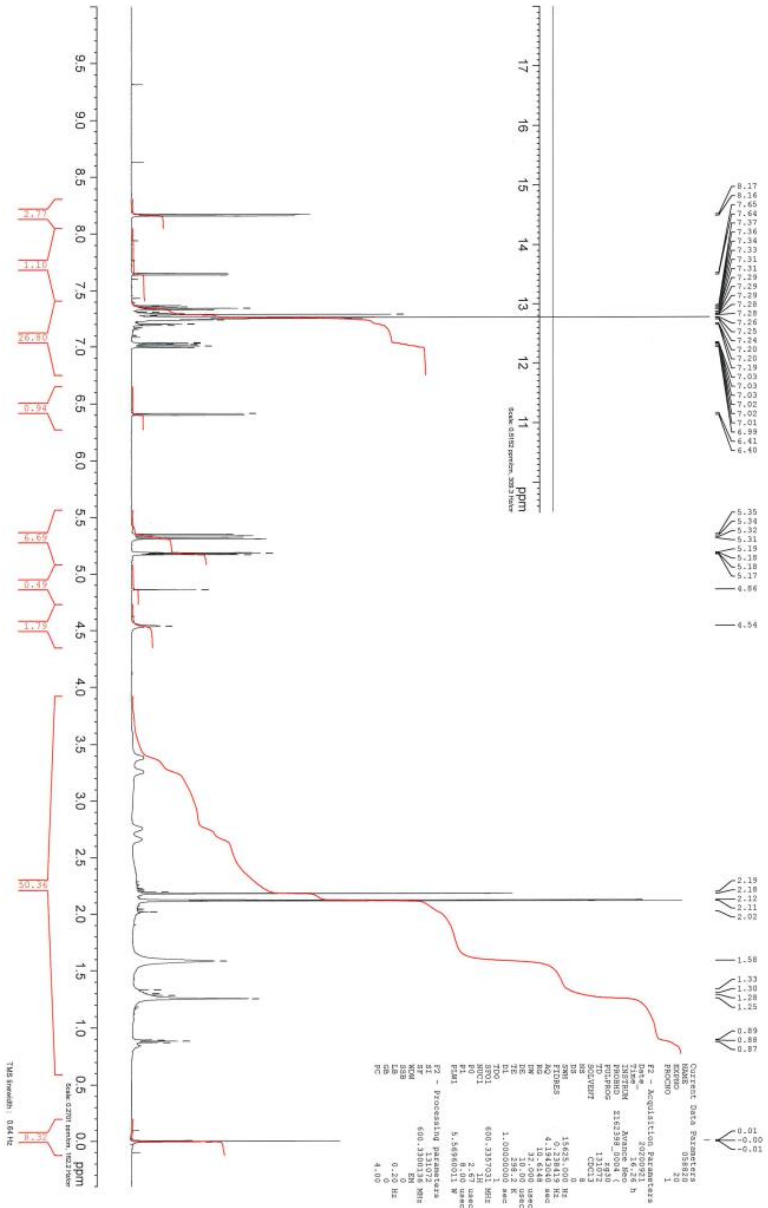

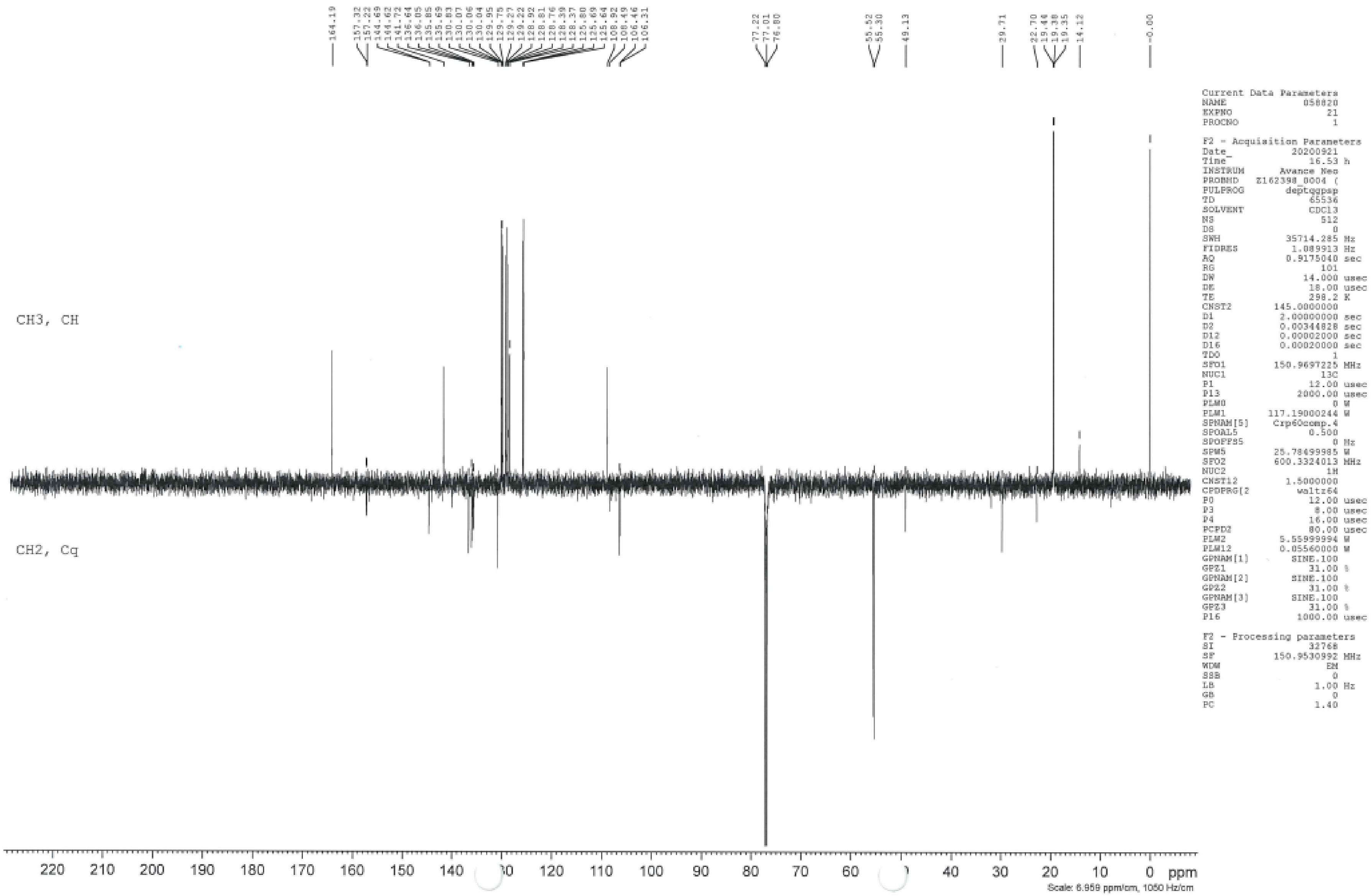
164.19
157.32
157.22
144.69
144.62
141.72
136.64
136.05
135.85
135.69
130.83
130.07
130.06
130.04
129.95
129.75
129.27
129.22
128.92
128.81
128.76
128.39
128.37
125.80
125.69
125.64
108.92
108.49
106.46
106.31
77.22
77.01
76.80
55.52
55.30
49.13
29.71
22.70
19.44
19.38
19.35
14.12
-0.00
CH3, CH
CH2, Cq
Current Data Parameters
NAME 058820
EXPNO 21
PROCNO 1
F2 - Acquisition Parameters
Date_ 20200921
Time 16.53 h
INSTRUM Avance Neo
PROBHD Z162398_0004 (
PULPROG deptqgpsp
TD 65536
SOLVENT CDCl3
NS 512
DS 0
SWH 35714.285 Hz
FIDRES 1.089913 Hz
AQ 0.9175040 sec
RG 101
DW 14.000 usec
DE 18.00 usec
TE 298.2 K
CNST2 145.0000000
D1 2.00000000 sec
D2 0.00344828 sec
D12 0.00002000 sec
D16 0.00020000 sec
TD0 1
SFO1 150.9697225 MHz
NUC1 13C
P1 12.00 usec
P13 2000.00 usec
PLW0 0 W
PLW1 117.19000244 W
SPNAM[5] Crp60comp.4
SPOAL5 0.500
SPOFFS5 0 Hz
SPW5 25.78499985 W
SFO2 600.3324013 MHz
NUC2 1H
CNST12 1.5000000
CPDPRG[2 waltz64
P0 12.00 usec
P3 8.00 usec
P4 16.00 usec
PCPD2 80.00 usec
PLW2 5.55999994 W
PLW12 0.05560000 W
GPNAM[1] SINE.100
GPZ1 31.00 %
GPNAM[2] SINE.100
GPZ2 31.00 %
GPNAM[3] SINE.100
GPZ3 31.00 %
P16 1000.00 usec
F2 - Processing parameters
SI 32768
SF 150.9530992 MHz
WDW EM
SSB 0
LB 1.00 Hz
GB 0
PC 1.40
220 210 200 190 180 170 160 150 140 130 120 110 100 90 80 70 60 50 40 30 20 10 0 ppm
Scale: 6.959 ppm/cm, 1050 Hz/cm

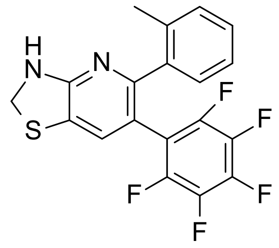

17f

5-(2-Fluorophenyl)-6-pentafluorophenyl-2,3-dihydro[1,3]thiazolo[4,5-*b*]pyridine (**17**f, 24 mg, 2%). ^{1}H NMR (400 MHz, $CDCl_3$): δ 2.20 (s, 3H), 4.55 (s, 2H), 6.58 (br. s, 1H, NH), 7.16-7.18 (m, 1H), 7.21–7.31 (m, 4H) ppm; HRMS (ESI, m/z): calcd. for $C_{19}H_{11}F_5N_2S$ $[M]^+$ 394.0563; found 394.0552.

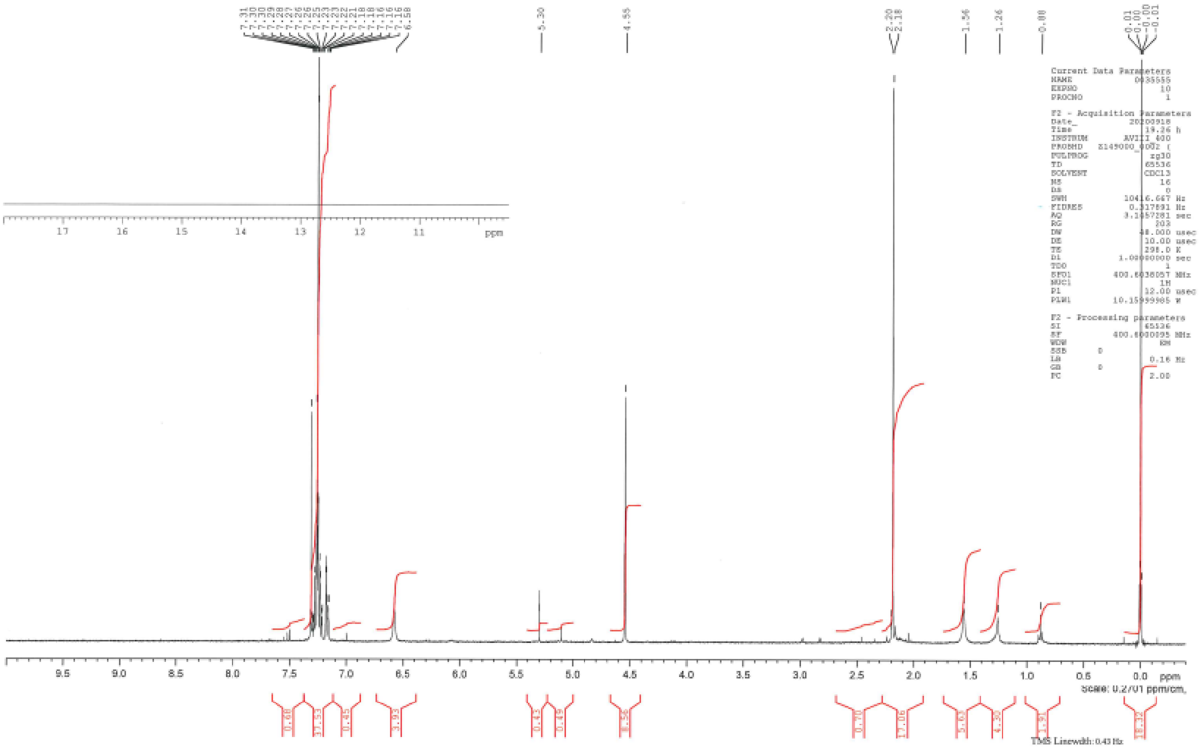

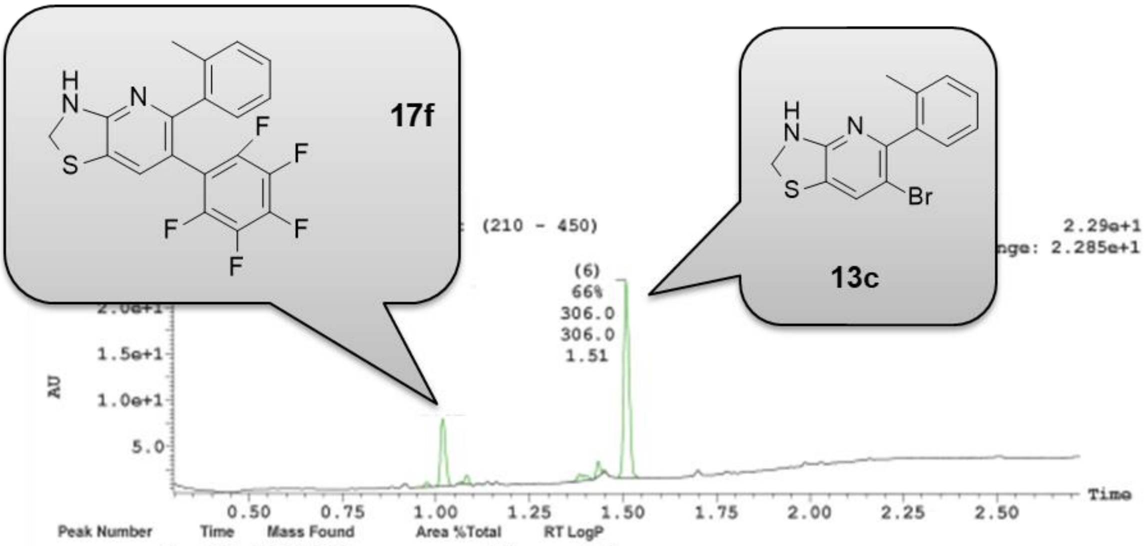

Peak Number	Time	Mass Found	Area %Total	RT LogP
1	0.98	326, 305.9826	1.42	1.40
2	1.02	326, 305.9826	21.95	1.52
3	1.08	326, 305.9826	2.73	1.66
4	1.38	305.9826	3.83	2.62
5	1.43	326, 305.9826	3.89	2.80
6	1.51	326, 305.9826	66.19	3.13

Peak ID	Time	Mass Found
1	0.98	348,307

1:(Time: 0.98) Combine (131:138-109:123) 1:MS ES+ 5.6e+006

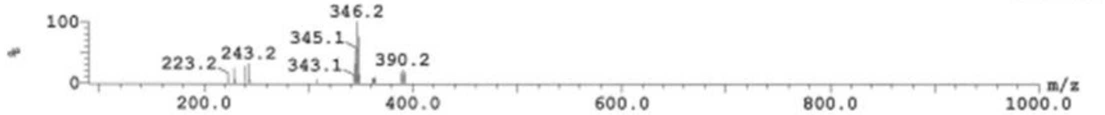

Peak ID	Time	Mass Found
1	0.98	305

1:(Time: 0.98) Combine (130:137-108:122) 2:MS ES- 6.3e+004

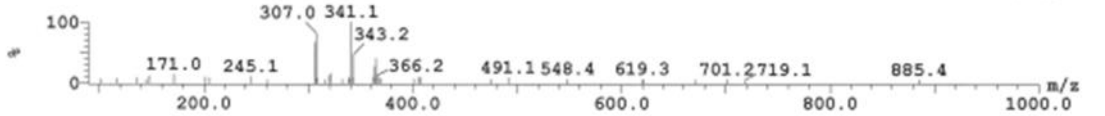

Peak ID	Time	Mass Found
2	1.02	348

2:(Time: 1.02) Combine (137:143-116:130) 1:MS ES+ 2.1e+007

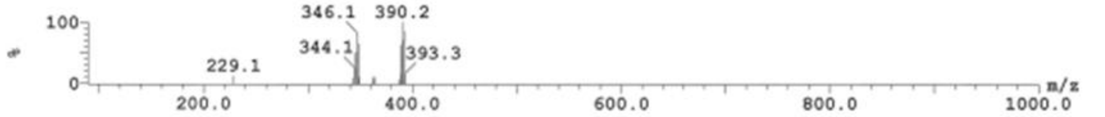

2. Biology and biochemistry

2.1. In vivo greenhouse screening

Seeds of mono- and dicotyledonous weed plants and crop plants were sown in plastic or organic planting pots in sandy loam and covered with soil (replicates: $n = 10$, i.e., 10 monocotyledonous weed seeds were grown per pot or $n = 5$, i.e., 5 dicotyledonous weed seeds were grown per pot). The 2,3-dihydro[1,3]thiazolo[4,5-*b*]pyridines described above (e.g., **7a**–**c**, **13a**–**c**, **14a**–**c**, **16a**–**f**, and **17a**–**b**), formulated in the form of wettable powder (WP) , were applied to the surface of the covering soil as aqueous suspension or emulsion, with the addition of 0.5% of an additive at an application rate of 600 L of water/ha (converted). Following treatment, the pots were placed in a greenhouse and kept under optimum growth conditions for the test plants. The test plants were placed in the greenhouse for ca. three weeks under optimum growth conditions, and then the effect of the preparations was assessed visually in comparison to untreated control plants (herbicidal effect: 100% = plants died off, 0% = as untreated control plants). Efficacy values were given based on a rating scale by final visual experts' assessment of green mass, i.e., 5 ≥ 90% inhibition, 4 = 70–89% inhibition, 3 = 50–69% inhibition, 2 = 40–49% inhibition, 1 = 21–39% inhibition, and — < 20% inhibition. Advanced screening was carried out with or as emulsifiable concentrate formulations, three replicate pots, and a standardized number of seeds per pot depending on the plant species (10 seeds for corn and wheat spectrum). The harmful plants and crops used in greenhouse tests were the following species: *Alopecurus myosuroides* (ALOMY), resistant *Alopecurus myosuroides* (ALOMY_R, origin: Germany), *Apera spica-venti* (APESV), *Brachiaria platyphylla* (BRAPP), *Bromus tectorum* (BROTE), *Echinochloa crus-galli* (ECHCG), *Digitaria sanguinalis* (DIGSA), *Eleusine indica* (ELEIN), *Glycine max* (GLXMA), *Lolium rigidum* (LOLRI), *Lolium sp.* (LOLSS), resistant *Lolium sp.* (LOLSS_R, origin: France), *Poa annua* (POAAN), *Setaria viridis* (SETVI), *Sorghum halepense* (SORHA), *Triticum aestivum* (TRZAS), and *Zea mays* (ZEAMX).

2.2. Biochemistry

2.2.1. *Lp*FAT A expression and purification

The *fat a03* gene from *Lemna paucicostata*, in which the N-terminal amino acids representing the chloroplast transit peptide were replaced by an N-terminal 6xHis-tag, was cloned into a pET24 vector [1]. The *Lp*FAT A protein was expressed in *E. coli* BL21Star(DE3) cells. 5 mL of an overnight culture of *E. coli* cells grown in LB medium with 100 µg/mL carbenicillin were used to inoculate 0.5 L of autoinduction medium containing 100 µg/mL carbenicillin [2]. The bacteria were grown at 37 °C and 120 rpm for about 4.5 h to reach OD_{600} = 0.6 and then further cultivated at 21 °C overnight. The bacteria were harvested by centrifugation (20 min, 6,000 g) and stored frozen at −80 °C. *Lp*FAT A protein was purified using the Ni-NTA Fast Start Kit (Qiagen GmbH, Germany) according to the instructions of the manufacturer. Active fractions were pooled together, and the buffer was exchanged into 25 mM potassium phosphate buffer pH 7.3 containing 10% glycerol with PD10 columns (GE Healthcare). Aliquots of the protein solution were frozen in liquid nitrogen and stored at −80 °C.

2.2.2. *Lp*FAT A fluorescence polarization assay

Fluorescence polarization (FP) competition assays were performed at room temperature in black 96-well microtiter plates (Greiner, Catalog No. 655900). The assay mixture contained 25 mM potassium phosphate buffer pH 7.3, 200 mM NaCl, 0.01% Triton X-100, 2 nM fluorescent tracer, 0.4 µg of purified *Lp*FAT A protein and different amounts of the test compound in a total volume of 100 µL. FP was measured with a BMG CLARIOstar microtiter plate reader using the FP filter set for fluorescein (Ex 482-16, Em 530-40, LP504). FP is the difference between wells containing *Lp*FAT A and wells containing only tracer. The pI_{50} values were calculated from plots of inhibition values vs test compound concentration using Model 205 of the ID Business Solutions Ltd Xlfit software suite. The FAT A binding fluorescent tracer was synthesized from (2*S*,4*S*)-4-[(2,6-difluorophenyl)methoxymethyl]-4-ethyl-2-methyl-*N*-(prop-2-ynylcarbamoyl)-1,3-dioxolane-2-carboxamide [1] and FAM azide, 5-isomer (Broadpharm BP-22544, San Diego, CA) by click chemistry [3] and was purified by flash column chromatography on silica gel.

3. References

[1] Campe R, Hollenbach E, Kämmerer L, Hendriks J, Höffken H W, Kraus H, Lerchl, J, Mietzner, T, Tresch, S, Witschel, M, Hutzler, J. A new herbicidal site of action: Cinmethylin binds to acyl-ACP thioesterase and inhibits plant fatty acid biosynthesis. *Pesticide Biochemistry and Physiology* **148**: 116–125 (2018).

[2] Studier F W. Protein production by auto-induction in high-density shaking cultures. *Protein Expr. Purif.* ***41***: 207-234 (2005).

[3] Punna S, Kaltgrad E, Finn M G. "Clickable" Agarose for Affinity Chromatography. *Bioconjugate Chem.* **16**: 1536-1541 (2005).
